# Design and Synthesis of Water-Soluble and Potent MMP-13 Inhibitors with Activity in Human Osteosarcoma Cells

**DOI:** 10.3390/ijms22189976

**Published:** 2021-09-15

**Authors:** Jose Maria Zapico, Lourdes Acosta, Miryam Pastor, Loganathan Rangasamy, Laura Marquez-Cantudo, Claire Coderch, Irene Ortin, Maria Nicolau-Sanus, Leonor Puchades-Carrasco, Antonio Pineda-Lucena, Alejandro Majali-Martinez, Pilar Ramos, Beatriz de Pascual-Teresa, Ana Ramos

**Affiliations:** 1 Departamento de Química y Bioquímica, Facultad de Farmacia, Universidad San Pablo-CEU, CEU Universities, Urbanización Montepríncipe, 28925 Alcorcón, Spain; josemaria.zapicorodriguez@ceu.es (J.M.Z.); l.acosta1@usp.ceu.es (L.A.); myriampastor@ucm.es (M.P.); loganathan.r@vit.ac.in (L.R.); laura.marquezcantudo@ceu.es (L.M.-C.); claire.coderchboue@ceu.es (C.C.); irene.ortinremon@ceu.es (I.O.); a.majali-martinez@medunigraz.at (A.M.-M.); pramos@ceu.es (P.R.); 2 Centre for Biomaterials, Cellular and Molecular Theranostics (CBCMT), Vellore Institute of Technology (VIT), Vellore 632014, India; 3 Drug Discovery Unit, Instituto de Investigación Sanitaria La Fe, 46026 Valencia, Spain; manisa@ibmcp.upv.es (M.N.-S.); leonor_puchades@iislafe.es (L.P.-C.); 4 Centro de Investigación Médica Aplicada, Molecular Therapeutics Program, Universidad de Navarra, 31008 Pamplona, Spain; apinedal@unav.es; 5 Department of Obstetrics and Gynecology, Medical University of Graz, 8036 Graz, Austria

**Keywords:** *MMP-13* inhibitors, metalloproteinases, osteoarthritis, molecular modeling, RMN, organic synthesis

## Abstract

Osteoarthritis is a degenerative disease, often resulting in chronic joint pain and commonly affecting elderly people. Current treatments with anti-inflammatory drugs are palliative, making the discovery of new treatments necessary. The inhibition of matrix metalloproteinase *MMP-13* is a validated strategy to prevent the progression of this common joint disorder. We recently described polybrominated benzotriazole derivatives with nanomolar inhibitory activity and a promising selectivity profile against this collagenase. In this work, we have extended the study in order to explore the influence of bromine atoms and the nature of the S1′ heterocyclic interacting moiety on the solubility/selectivity balance of this type of compound. Drug target interactions have been assessed through a combination of molecular modeling studies and NMR experiments. Compound **9a** has been identified as a water-soluble and highly potent inhibitor with activity in MG-63 human osteosarcoma cells.

## 1. Introduction

Matrix metalloproteinases (MMPs) are zinc-dependent endopeptidases responsible for the degradation and remodeling of the extracellular matrix. Currently, there are 23 different MMPs known in humans, which are classified into different groups based on their specificity of substrate [1,2]. *MMP-13* is a member of the collagenase group, together with *MMP-1* and *MMP-8* [3], and is deregulated in different disorders, such as osteoarthritis (OA) [4,5,6,7], rheumatoid arthritis [6,8], obesity [9], and cancer [10,11,12].

OA is characterized by the progressive degeneration of the joint cartilage and constitutes the most common joint disorder that affects the middle-aged and elderly population [13,14]. The main symptoms of the disease are pain and loss of function [15,16]. Current treatments for OA with steroidal and nonsteroidal anti-inflammatory drugs are palliative, and surgical approaches are required when the joints become dysfunctional [17,18,19]. *MMP-13* is involved in OA through the degradation of type II collagen, the main structural protein of articular cartilage [20]. It has been reported that *MMP-13* levels in synovial fluid are correlated to human OA severity [21]. *MMP-13* is expressed in the cartilage of human OA patients while it is not present in normal adult cartilage [5,22]. Altogether, this makes the inhibition of *MMP-13* an effective strategy to prevent the progression of OA [21,23,24]. The fact that OA brings patients great pain and high healthcare costs [25], plus the fact that there are currently no FDA-approved *MMP-13* inhibitors to cease the development of the disease, clearly supports the need for research in this field.

**Figure 1:**
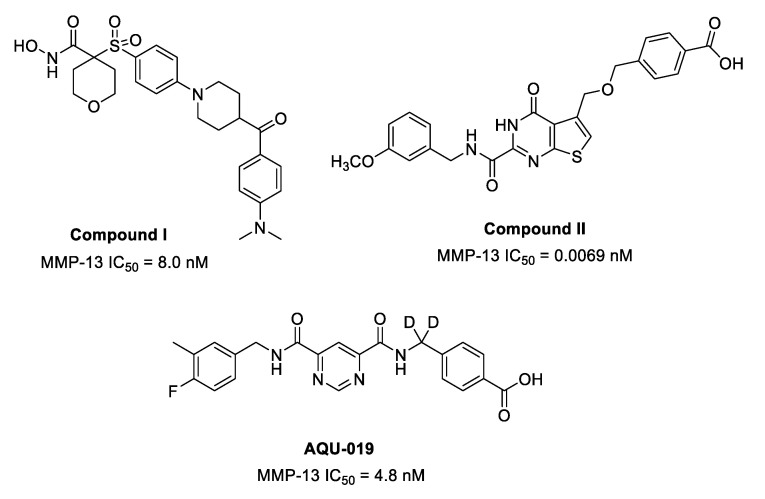
Structure of three published MMP-13 inhibitors.

All MMPs have a Zn atom in the active site, coordinated by three histidines and surrounded by several subsites. Among them, the S1’subsite is known as the specificity subsite because it differs in length and amino acid sequence in the different MMPs. *MMP-13* possesses a large S1´pocket, bigger than in other MMPs, which can accommodate large substituents, giving the opportunity of gaining selectivity. One strategy in the design of selective *MMP-13* inhibitors consists of using a Zinc Binding Group (ZBG) to chelate the Zn atom present in the active site of the enzyme (very frequently a hydroxamate, as in compound I [26]) (Figure 1), connected with large groups selected to interact with the S1´pocket. This characteristic S1´pocket has also allowed the development of non-zinc-binding *MMP-13* inhibitors using different structural scaffolds, as in compound II [27] (Figure 1). More examples of *MMP-13* inhibitors of the above-mentioned strategies are compiled in X.-W Xie et al. [13] and Y. Wan et al. [17] reviews. The most recent publications in this field describe new selective *MMP-13* allosteric inhibitors based on pyrimidines and triazolopyrimidines scaffolds [28,29]. Interestingly, A. M. Bendele et al. demonstrated a chondroprotective effect of the AQU-019 inhibitor in a rat model of [28] OA (Figure 1).

A novel approach in the search for selective *MMP-13* inhibitors is the use of docking techniques to carry out the screening of FDA-approved drugs against optimal human *MMP-13* crystal structures. Thus, K. S. Kumar et al. have found three potential *MMP-13* inhibitors that can be repurposed as drugs for OA therapy [30], saving time and costs in the drug development process. Other recent investigations are focused on the use of *MMP-13* inhibitors for the development of probes as non-invasive techniques that allow an early diagnosis and a better prognosis of OA. Significant examples that follow this line of research are described in the works by S. Lee et al. [31] and V. Hugenberg et al. [32].

**Figure 2:**
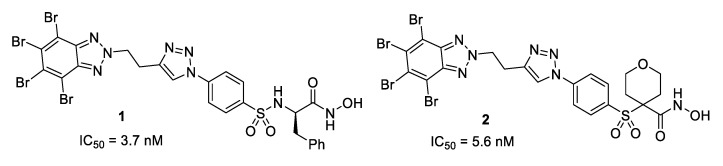
Chemical structure and IC_50_ values for compounds **1** and **2**.

In previous work, we described polybrominated triazole derivatives **1** and **2** (Figure 2) with nanomolar inhibitory activity against *MMP-13* (3.7 and 5.6 nM, respectively) and promising selectivity profiles against other MMPs [33]. In this work, we have extended the study to inhibitors based on phthalimide scaffolds and explored the influence of the bromine atoms with the aim of improving the potency/selectivity/solubility balance of this type of compound.

## 2. Results and Discussion

### 2.1. Synthesis

**Figure 3:**
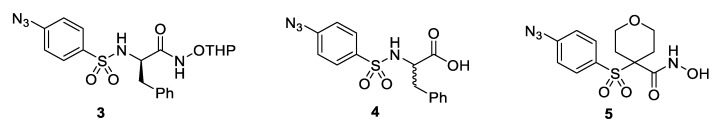
Chemical structure of compounds **3**–**5**.

Azides **3, 4,** and **5,** necessary for the click chemistry reactions, were synthesized as described in previous works [34,35] (Figure 3).

**Scheme 1:**
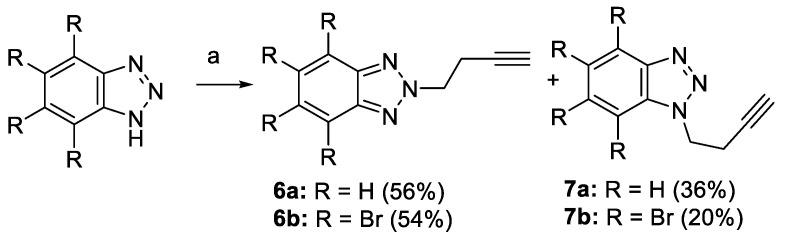
Synthesis of the benzotriazole-derived alkynes. *Reagents and conditions*: (a) ACN, K_2_CO_3_, 4-bromobut-1-yne, 24 h, RT.

Triazole-derived alkynes **6a,b** were obtained by alkylation of benzotriazole or tetrabromobenzotriazole with 4-bromobut-1-yne in the presence of potassium carbonate. Together with the desired *N*-2 alkylated compounds **6a,b**, the corresponding *N*-1 alkylation products **7a,b** were obtained (Scheme 1) [36].

**Scheme 2:**
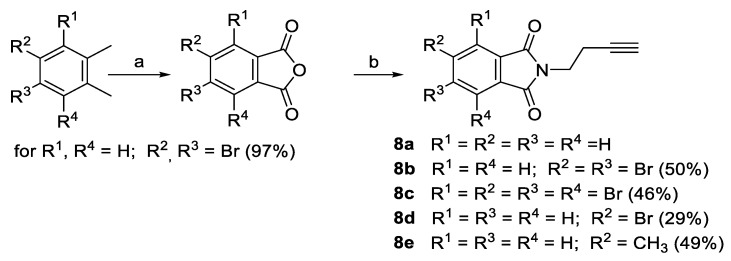
Synthesis of the phthalimide-derived alkynes. *Reagents and conditions*: (**a**) (i) KMnO_4_, KOH, H_2_O, 120 °C, MW (ii) Ac_2_O, 80 °C, MW (**b**) 3-butyn-1-amine, EtOH, 100 °C, MW.

The alkynes derived from phthalimides used in the synthesis of the final inhibitors are collected in Scheme 2. *N*-(3-butynyl) phthalimide (**8a**) is commercially available, and the rest of the alkynes (**8b–e**) were synthesized by reaction of the corresponding anhydride with 3-butyn-1-amine. All the anhydrides were commercially available except the dibromosubstituted (R^2^ = R^3^ = Br), which was obtained by oxidation followed by dehydration of 1,2-dibromo-4,5-dimethylbenzene.

**Scheme 3:**
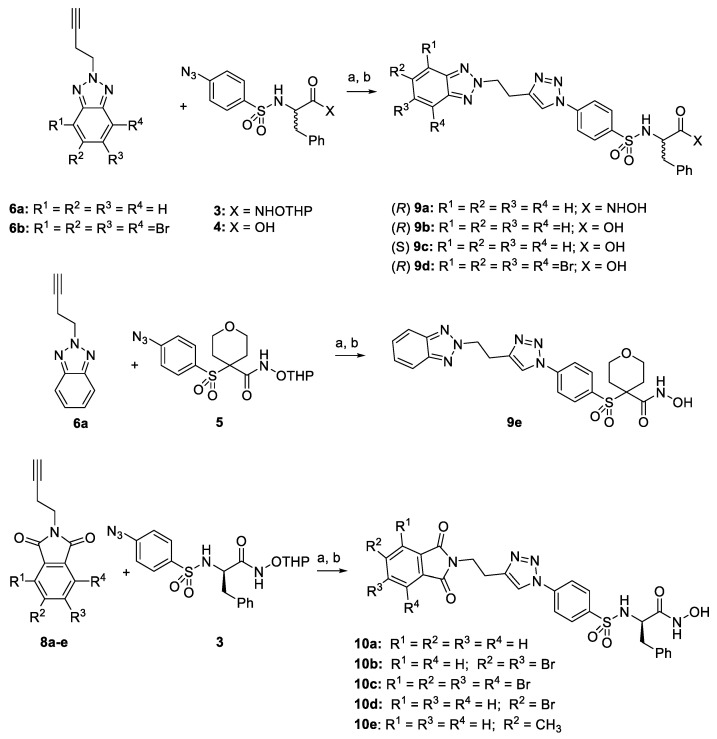
Synthesis of clicked acids and hydroxamates*^.^ Reagents and conditions*: (**a**) CuSO_4_, sodium ascorbate, DMF (and TBTA for the synthesis of (**9b**–**d**) (**b**) HCl in MeOH, 0 °C for (**9a**–**e**), (**10a**–**e**); AcOH, THF, H_2_O, 45 °C MW radiation for (**10b**–**d)**.

The click chemistry reaction between azides and alkynes was performed using CuSO_4_ and ascorbate as a catalytic system. When the click reaction was performed with azides containing a carboxylic group, 0.01 equivalents of TBTA (Tris [(1-benzyl-1*H*-1,2,3-triazol-4-yl) methyl] amine) was added to the reaction mixture yielding acids **9b–d** with good yields. In the case of hydroxamates **9a** and **10a–e**, a final THP-deprotection step was performed in acidic conditions (Scheme 3).

### 2.2. Activity Results

In a previous publication, we reported the discovery of a new class of compounds that showed highly potent activity against *MMP-13*, and a promising selectivity profile against a panel of MMPs. Thus, hydroxamates **1** and **2** presented activity in the low nanomolar range with IC_50_ = 3.7 and 5.6 nM, respectively (Table 1). The activity of hydroxamate **1** against *MMP-13* was the highest among all the tested MMPs (for selectivity of **1** see Table 2) [33].

Both **1** and **2** present the calculated logD (clogD) values of 6.01 and 4.44, respectively, and moderate solubility in water at physiological pH (Table 1), predicting bad absorption and difficult administration. In our studies on *MMP-2* inhibitors, we could improve solubility by modifying the structure of the initial hits by introducing a polar piperidine ring [37].

To improve the solubility of **1** and **2**, we designed and synthesized compounds **9a** and **9e**, where we removed all bromine atoms present in these molecules. Fortunately, we observed an increase in *MMP-13* inhibitory activity, with IC_50_ values of 0.65 and 3.3 nM, respectively. Furthermore, the predicted solubility classifies **9e** as water-soluble (Calibration curves of all active compouds are collected in Supplementary Material (Figure S1).

The removal of the bromine atoms in **9a** caused a loss of selectivity, showing nanomolar activity against *MMP-2* and *MMP-12*. We expected this result because *MMP-13* has a larger and slightly more flexible Ω-loop compared to that of other MMPs [38]. Therefore, the bulky tetrabromo benzotriazole group present in **1** and **2** is better stabilized by *MMP-13* [33], while non-brominated compounds **9a** and **9e** bind to the active site of other isoforms with similar affinity. The results of the activity of **9a** against a panel of MMPs are collected in Table 2. This inhibitor shows selectivity against *MMP-1*, an enzyme reported to be involved in the musculoskeletal disorders described in patients treated with broad-spectrum MMP inhibitors [39]. Inhibitor **9a** is also selective against *MMP-14* and moderately selective against *MMP-3*,7,8, and 10.

To analyze whether the selectivity could improve using a weaker Zinc Binding Group (ZBG), we replaced the bidentate hydroxamate ZBG with a carboxylic acid. Thus, we synthesized and tested acids **9b–d**, but we found a complete lack of inhibitory activity against *MMP-13*.

The next step in this work was the modification of the aromatic core. We selected compounds **10a–e** due to the accessibility to a variety of phthalimides with different bromine substitution patterns. Furthermore, we considered that the presence of hydrogen bond acceptors (carbonyl groups) in these compounds could increase the affinity for *MMP-13* by establishing interactions with some residues in the active site of the enzyme (see molecular modeling studies).

As can be seen in Table 1, all phthalimide derivatives were active in *MMP-13*. The lowest activity was obtained for tetrabromo derivative 10c, which also had the worst predicted pharmacokinetic properties. The progressive removal of bromine atoms in **10b,d** and **10a** gave an improvement in activity, clogD, and solubility. Inhibitor **10a** was the best compound of this series with IC_50_ of 6.9 nM, clogD = 2.37, and moderate calculated solubility. Finally, compound **10e**, which bears a methyl group at the 5-position of the phthalimide ring, was also active in *MMP-13* (IC_50_ = 9.2 nM), demonstrating that it is possible to introduce larger substituents in this part of the molecule.

All the above-mentioned solubility values predicted with Chemicalize showed a good correlation with the experimental values obtained in PBS pH 7.4 (Table 1). Although solubility values are slightly lower than expected, the solubility gradation maintains the same trend. The most soluble compound was **9e,** followed by **9a** and **10a–d**.

### 2.3. Molecular Modeling

The catalytic domain of all MMPs shares the same common features, the catalytic Zn^2+^ ion at the entrance of the catalytic cleft and, delimiting the lower lip of that cleft, the flexible Ω-loop, also known as the specificity loop. This loop is the least conserved fragment within the catalytic domain and defines the size of the ligand-binding site, the S1′ pocket; thus, it has become the center of attention of studies looking for ligand specificity [40]. In our experience with selective *MMP-2* inhibitors, we found that straight and rather rigid molecules protrude out of the Ω-loop, establishing mainly hydrophobic interactions with the side chains of the amino acids that line it [35]. However, we obtained *MMP-13* binding when the ligand presented flexibility that enabled it to adapt to the S1′ pocket, as the increased length and flexibility of the Ω-loop allowed a certain expansion of the binding site. Consequently, in contrast to *MMP-2*, *MMP-13* will be able to accommodate within the S1′ pocket larger and bulkier ligands, such as the polybrominated **1** and **2**, thus leading to lower IC_50_ values (Table 1) [33].

Working with MMPs is always a challenge, as the conformation of the Ω-loop is determined by the nature and size of the bound ligand and the induced fit that it generates upon binding. Most molecular modeling methods are deterministic, as the outcome greatly depends on the initial structure provided. Therefore, in this work, we have used the ensemble docking approach to model as many interactions with the Ω-loop as possible. In this way, we recreate what happens in a flexible system accounting for different binding interactions of the highly modified moieties of the synthesized compounds and the Ω-loops of *MMP-13* and *MMP-2*. To account for the Ω-loop flexibility, we analyzed the PDB to find deposited structures of *MMP-13* and *MMP-2* bound to different ligands that would have produced different induced fits, and therefore, different conformations of the Ω-loop. We selected four *MMP-13* structures, as well as the only NMR-solved *MMP-2* structure available that presented eleven different conformations. The selected *MMP-13* structures were bound to three different types of inhibitors that produced different conformations of the *n*-terminal end of the Ω-loop: (i) a short chelating inhibitor (PDB code 456C), (ii) a long chelating rigid inhibitor (PDB code 3TVC), and (iii) two large non-chelating inhibitors with a carboxylic moiety that explored the back of the S1′ pocket and interacted with the side chain of Lys140 (PDB codes 3KEK and 3WV1).

Previous studies in *MMP-13* showed how some inhibitors are able to follow the α-helix that makes up the inner wall of the S1′ site and to establish hydrophobic interactions with the binding site due to the increased length and flexibility of the Ω-loop [33]. In this line, the reason for the *MMP-13* selectivity of the polybrominated **1** and **2** is the flexibility of the Ω-loop and its inherent ability to accommodate the bulky TBB moiety within the S1′ pocket where it can establish hydrophobic interactions [33]. On the other hand, when binding to *MMP-2*, ligands tend to protrude out of the S1′ pocket and interact with the amino acids lining the loop [34,41].

**Figure 4:**
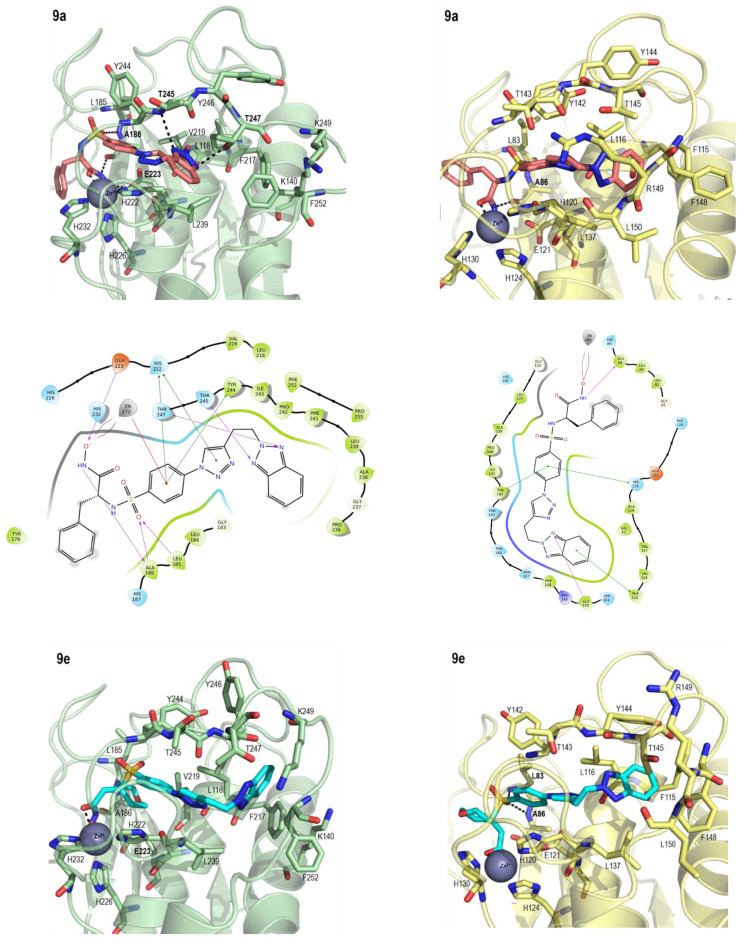

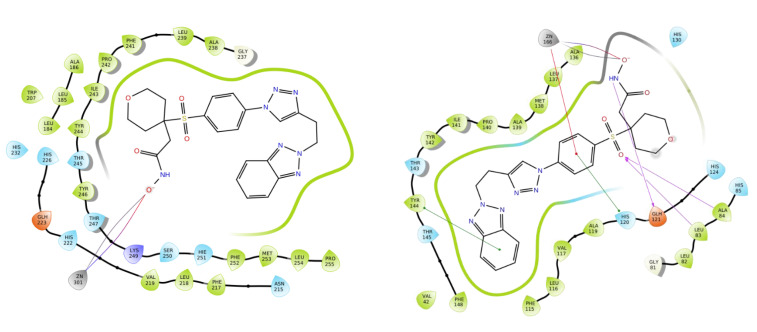
PyMOL stick and cartoon representation of the interactions established with the Ω-loop and S1′ pocket of the energetically most favorable binding pose of compounds **9a**,**e** bound to *MMP-13* (left) *MMP-2* (right). Hydrogen bonds are shown as dashed lines, and the catalytic zinc ion is shown as a grey sphere. For the sake of clarity, hydrogens are not shown.

**Figure 5:**
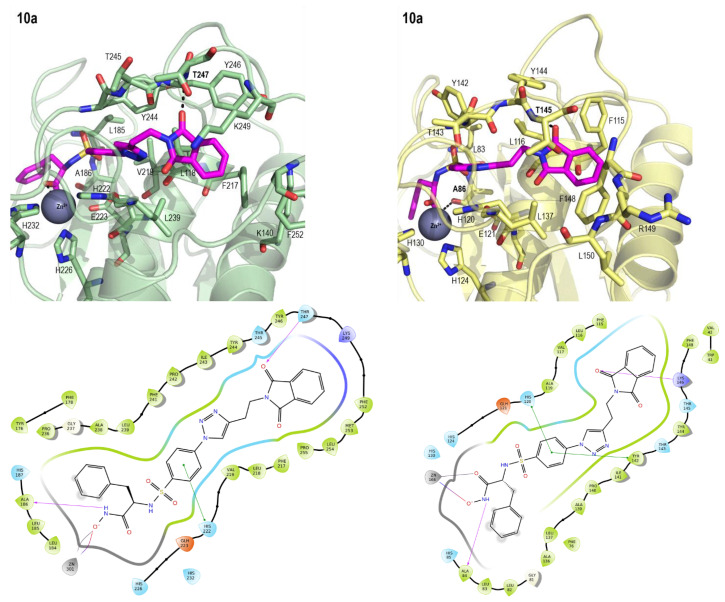

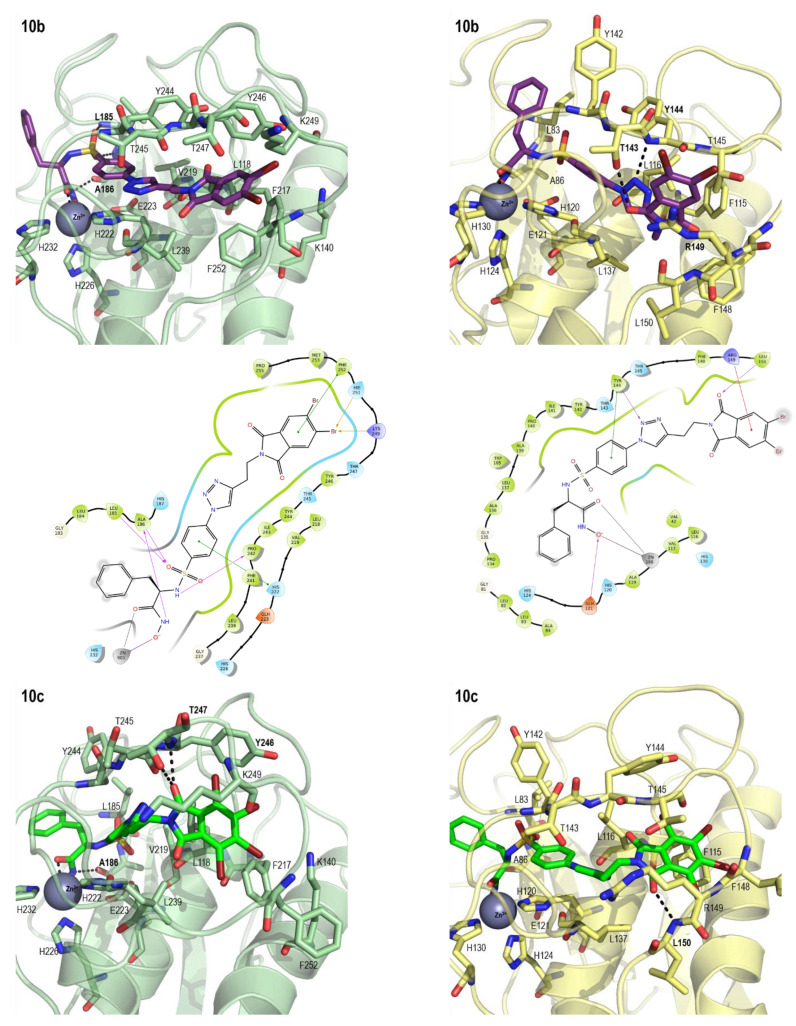

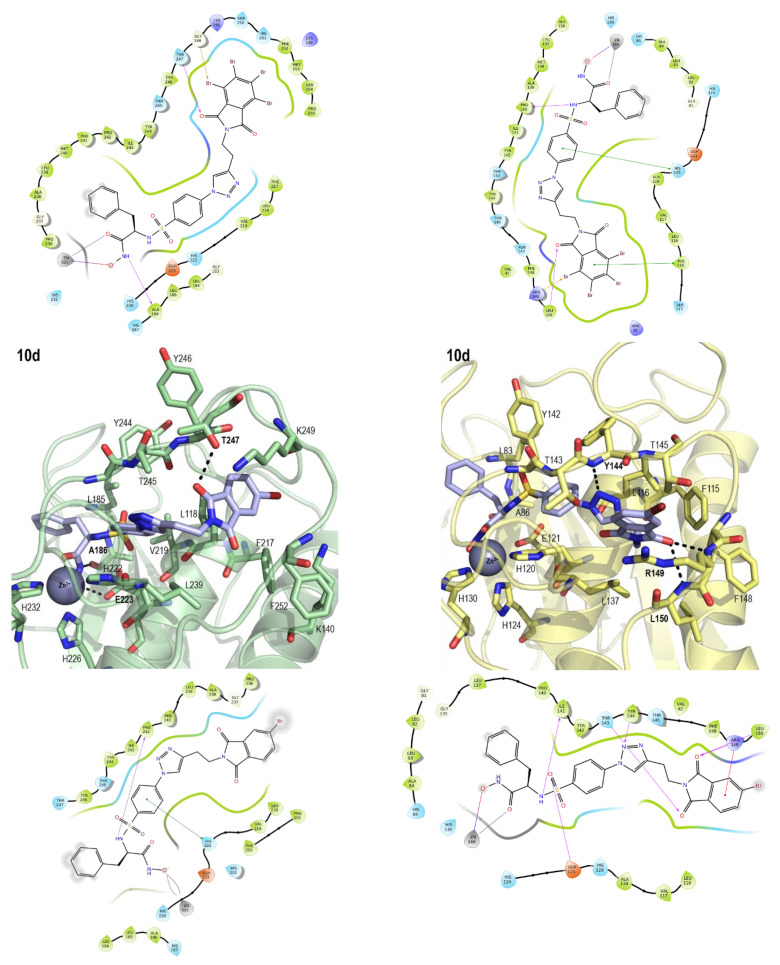

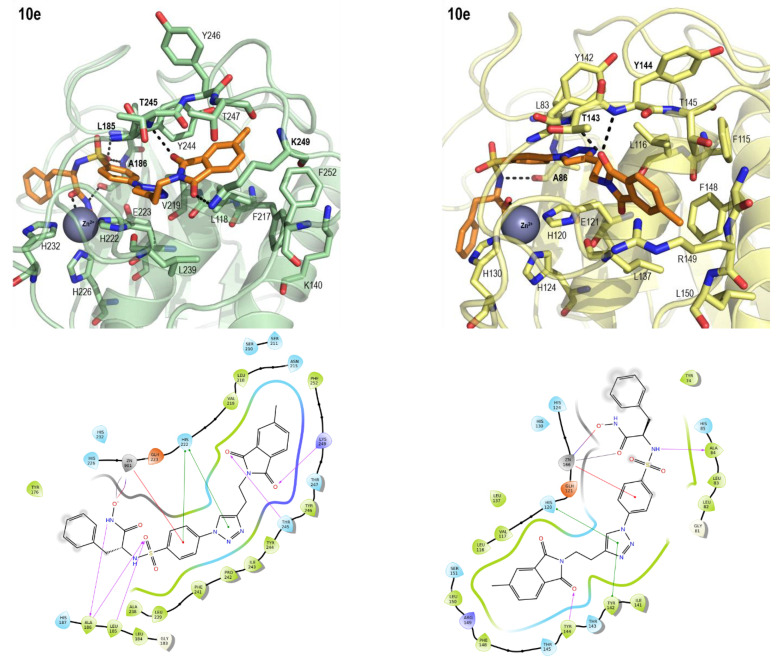
PyMOL stick and cartoon representation and 2D representation of the interactions established with the Ω-loop and S1′ pocket of the energetically most favorable binding poses of compounds **10a–e** bound to *MMP-13* (**left**) and *MMP-2* (**right**). Hydrogen bonds are shown as dashed lines, and the catalytic zinc ion is shown as a grey sphere. For the sake of clarity, hydrogens are not shown.

All the ligands were docked in all the selected protein conformations of both *MMP-13* and *MMP-2* to account for all the possible interactions established with the flexible Ω-loop (Figures S3 and S4). All docked compounds established a bidentate chelation with the catalytic zinc ion (Figure S5) and oriented the rest of the molecule towards the interior of the S1′ pocket. The best binding poses were selected to account for the ligand-receptor interactions; their docking scores are summarized in Supplementary Table S1. Following previous results [33,34], the smaller size of the benzotriazole compared to that of the TBB moiety increases the binding affinity of both **9a,e** compared to that of **1** and **2** (Table 1). However, the selectivity for *MMP-13* over *MMP-2* decreases as the smaller-sized **9a**–**e** can fit in both *MMP-13* and *MMP-2* (Figure 4). The modification of the aromatic core led to compounds 10**,** where the presence of carbonyl groups in the phthalimide moiety allowed better interactions with the S1′ pocket through the establishment of hydrogen bonds with the amino acids lining the Ω-loop of *MMP-2* (Figure 5). Unfortunately, we observed a higher affinity for *MMP-2* compared to *MMP-13* for this series of compounds (Table 1). As shown in Figure 5, compound 10a binds to *MMP-2*, positioning the phthalimide moiety closer to the hydrophilic moieties than in *MMP-13*.

Further modifications of the phthalimide by the addition of bromine atoms in **10b**, **10c,d**, or a methyl group in **10e** (Figure 5) did not improve binding in the hydrophobic S1´pocket and, as a result, the binding affinity towards *MMP-13* is reduced compared to that of **10a** (Table 1). The addition of the hydrophobic substituents to the phthalimide reduced the affinity, for *MMP-2*, as the bromine atoms make the fitting inside the S1′ pocket difficult, and only one binding pose was obtained for **10c** in the eleven NMR models of *MMP-2* (Figure S4) [33].

### 2.4. NMR Studies

To experimentally assess if the observed inhibition of *MMP-13* was due to a physical interaction with the enzyme, the most active and soluble inhibitor **9a** was selected for performing a 1D NMR experiment, WaterLOGSY, commonly used to evaluate potential drug-target interactions.

To this end, a recombinantly expressed catalytic domain of *MMP-13* was purified under denaturing conditions (Figure S6); the process lead to a very pure sample preparation. After protein refolding, the enzymatic activity of the recombinant protein was evaluated (Figure S7), and the results showed that the protein maintains its activity in these conditions.

**Figure 6:**
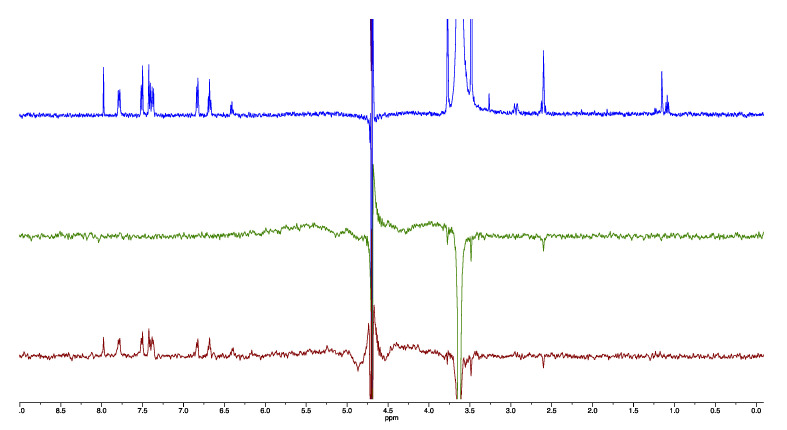
NMR studies corresponding to the interaction between the *MMP-13* catalytic domain and compound **9a**. Blue: 1D ^1^H spectrum of compound **9a** and the *MMP-13* catalytic domain; green: WaterLOGSY experiment of **9a**; red: WaterLOGSY experiment of **9a** in the presence of the *MMP-13* catalytic domain.

In WaterLOGSY experiments, the large bulk water magnetization is partially transferred via the protein-ligand complex to the free ligand in a selective manner. A non-interacting compound results in negative resonances, whereas productive protein-ligand interactions are characterized by positive signals or by a reduction in the negative signals obtained in the absence of the protein. In these experiments, (*2R*)-[(4-biphenylylsulfonyl)amino]-*N*-hydroxy-3-phenylpropionamide (BiPS), a commercially available MMP inhibitor, was used as a positive control. The results of the WaterLOGSY experiments revealed a pattern of positive signals, corresponding to specific chemical shifts of BiPS when the experiment was performed in the presence of the *MMP-13* catalytic domain, while no signals were detected in the absence of the protein (Figure S8). These results also corroborated the correct refolding of the recombinant protein and its interaction with a well-known inhibitor. More importantly, compound **9a**, the selected inhibitor, also showed a positive interaction with the *MMP-13* catalytic domain (Figure 6).

Overall, these results confirmed, using an orthogonal method, that a proper procedure was employed for the preparation of a functional *MMP-13* catalytic domain and that **9a** interacts with this domain leading to the inhibition of its biological activity.

### 2.5. MMP-13 Inhibition in MG-63 Cells

Next, we studied the inhibitory activity of **9a** in MG-63 human osteosarcoma cells because this is one of the most widely used cell lines in the study of the effect of drugs on osteoblasts [42].

**Figure 7:**
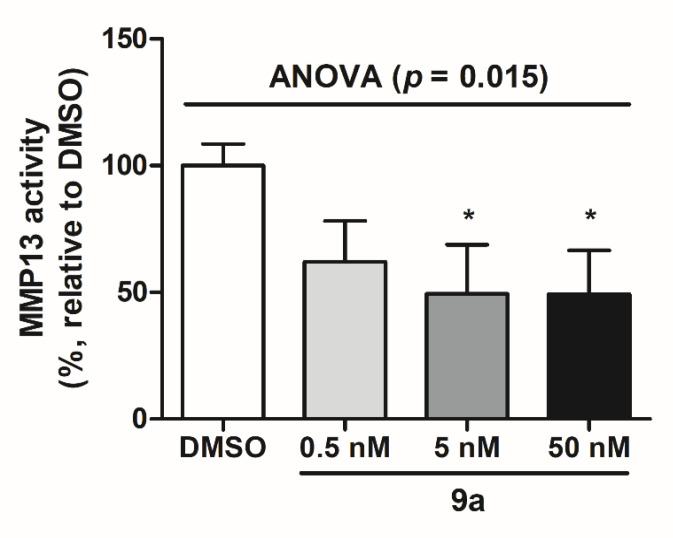
Effect of **9a** on *MMP-13* activity in MG-63 cells. MG-63 cells were incubated in the absence (DMSO) or the presence of **9a** (0.5, 5, and 50 nM). *MMP*-13 activity was determined in the cell supernatants using the SensoLyte^®^ Plus 520 *MMP-13* assay kit. Results were normalized to total protein content in the supernatants and calculated relative to the treatment with DMSO, considered as 100% in terms of *MMP-13* activity. Data are expressed as the mean ± SEM and are representative of three experiments. * *p* < 0.05 vs. DMSO.

*MMP-13* activity was determined on the conditioned medium from MG-63 cells treated with DMSO (i.e., control) or **9a**. When compared to the treatment with DMSO, **9a** showed a dose-dependent effect on *MMP-13* activity, with 0.5 nM **9a** decreasing *MMP-13* activity by 38% (non-significant), and 5 and 50 nM **9a** reducing *MMP-13* activity by 50% and 51%, respectively (*p* < 0.05) (Figure 7). No differences in *MMP-13* activity were observed between untreated MG-63 cells and MG-63 cells incubated with DMSO (data not shown).

This experiment demonstrates that compound **9a** is effectively decreasing the *MMP-13* activity in MG-63 human osteosarcoma cells and gives further evidence for the interest of this class of compounds in the treatment of osteoarthritis.

## 3. Conclusions

Based on previously described polybrominated benzotriazoles **1** and **2**, we have designed and synthesized a series of differently substituted benzotriazole and phthalimide derivatives. The removal of the bromine atoms present in polybrominated benzotriazoles **1** and **2** (compounds **9a,e**) significantly improved water solubility, as predicted by cheminformatics tools and subsequently determined by the shake flask method. Substitution of the aromatic core by a phthalimide scaffold (compounds **10**), containing carbonyl groups able to establish hydrogen bonds with the Ω loop of the enzyme, did not improve their solubility compared to **9a,e**, even in those compounds where all bromine atoms were removed (10a).

As computationally predicted, both compounds, **9a,e**, showed a loss of selectivity compared to the parent compound **1**. This fact is associated with the smaller size of the aromatic core that allows the accommodation of the inhibitors in the less flexible S1´selectivity pocket present in other metalloproteinases [38].

Substitution of the hydroxamate by a weaker ZBG not only brought about a loss of selectivity but also a complete loss of solubility.

Compound **9a**, in addition to its water solubility at physiological pH, showed a remarkable potency with IC_50_ = 0.65 nM in enzymatic assays. For this reason, we selected **9a** to carry out biophysical and cell-based assays.

WaterLOGSY NMR experiments corroborated the docking studies by showing a positive interaction of **9a** with the *MMP-13* catalytic domain.

MG-63 human osteosarcoma cells, one of the most widely used cell lines in the study of the effect of drugs on osteoblasts, were used to biologically evaluate the effect of **9a**, showing an effective decrease in the *MMP-13* activity

In conclusion, we have identified **9a** as a water-soluble and highly potent *MMP-13* inhibitor, which constitutes a new promising candidate for the treatment of osteoarthritis and related diseases.

## 4. Materials and Methods

### 4.1. Synthetic General Procedures

#### 4.1.1. Materials

All reagents and solvents were obtained from different commercial sources:Merck (Darmstadt, Germany), Fluorochem (Derbyshire, UK), Acros Organics (Geel, Belgium), Alfa Aesar (Candel, Germany), Scharlab (Barcelona, Spain) and Avantor (Radnor, PA, USA)and used without further purification. Tetrahydrofuran and dichloromethane were dried by the solvent purification system Technical Bulletin AL-258.

#### 4.1.2. General Methods

Reaction progress was monitored using analytical thin-layer chromatography (TLC) on Merck silica gel 60 F-254 plate. Visualization was achieved by UV light (254 nm). Flash chromatography was performed with Scharlau silica gel 60 (0.04–0.06 mm) packing. ^1^H NMR and ^13^C NMR spectra were recorded on a Bruker Advance III 400 MHz instrument. Signals are quoted as s (singlet), bs (broad singlet), d (doublet), t (triplet), q (quartet), dd (doublet of doublets), td (triplet of doublets), and m (multiplet). Chemical shifts (δ) are expressed in parts per million, relative to solvent resonance as the internal standard. Coupling constants (*J*) are in hertz (Hz). Original ^1^H NMR and ^13^C NMR spectra for final compounds are shown in Supplementary Materials (Figure S2) Melting points were determined on a Stuart Scientific (BIBBY) melting point apparatus. Final products were analyzed for purity by reverse-phase high-performance liquid chromatography (HPLC) on Agilent Technologies 1260 Infinity II apparatus (Figure S10). Mass spectrometry was performed by the CEMBIO Analytical Service Laboratory of the Universidad CEU San Pablo on an MS/IT Esquire 3000 Bruker Daltonics apparatus. Mass spectra reports for final compounds are shown in Supplementary Materials (Figure S11).

#### 4.1.3. General Synthetic Methods

1. Preparation of triazoles by Copper-Catalyzed Azide-Alkyne Cycloadditions (CuAAC)

Method A**:** To a suspension of azide **3** [34], **4** [34], or **5** [35] (1 eq) in DMF (10 mL/mmol), a freshly made aqueous 0.5 M sodium ascorbate solution (0.6 eq), aqueous 0.1 M CuSO_4_⋅5H_2_O solution (0.3 eq) (and TBTA (0.01 eq) for **9b–d**)**,** the corresponding alkyne (1.1–1.3 eq) was added and the reaction was stirred at RT overnight. The mixture was then concentrated under vacuum, and H_2_O was added. The precipitate formed was filtered and purified by column chromatography on silica gel.

Method B**:** To a suspension of azide **3** (1 eq) in THF (10 mL/mmol), sodium ascorbate (2 eq), CuSO_4_⋅5H_2_O (0.5 eq), and the corresponding alkyne (1.1–1.3 eq) were added, and the mixture was irradiated under microwave conditions at 70 °C for 30 h. The solid was removed by filtration, and the solvent was evaporated. The residue was purified by column chromatography on silica gel.

2. O-THP deprotection

Method A**:** HCl was prepared by adding AcCl (20 eq) to a solution of dry MeOH (1.5 mL) at 0 °C. The solution was stirred for 2 min, and then it was added to a flask containing the compound to be deprotected (1 eq) dissolved in MeOH (1 mL) at 0 °C. The mixture was kept at 0 °C till the completion of the reaction (C). The mixture was neutralized with saturated NaHCO_3_ aqueous solution. The precipitate formed was filtered and washed with water. The solid was purified when necessary.

Method B**:** A suspension of the THP-protected compound (1 eq) in THF (5 mL/mmol), H_2_O (2.5 mL/mmol), and AcOH (20 mL/mmol) was irradiated under microwave conditions at 45 °C for 4 h. The solvent was removed under vacuum, and the residue was purified by column chromatography on silica gel.

**5,6-dibromoisobenzofuran-1,3-dione** [43]. A suspension of 1,2-dibromo-4,5-dimethylbenzene (500 mg, 1.89 mmol), KMnO_4_ (1.8 g, 11.4 mmol), and KOH (180 mg, 3.2 mmol) in 20 mL of H_2_O was irradiated under microwave conditions at 120 °C for 15 h. After cooling down, EtOH was added, the mixture was filtered over silica gel, washed with water, and the solvent was evaporated. The residue was acidified, obtaining 4,5-dibromophthalic acid as a white solid isolated by filtration (252.7 mg, 41%). ^1^H RMN (400 MHz, D_2_O) δ 8.09 (s, 2H, Ar*H*); EM (ESI) *m/z* 322.4 [M-H]^−^. The solid obtained was suspended in Ac_2_O (5 mL) and heated under microwave conditions for 4 h at 80 °C. The solvent was evaporated under vacuum, and 5,6-dibromoisobenzofuran-1,3-dione was obtained as a white solid (231.5 mg, 97%). ^1^H RMN (400 MHz, CDCl_3_) δ 8.26 (s, 2H, Ar*H*)).

**5,6-dibromo-2- (but-3-yn-1-yl)isoindoline-1,3-dione (8b):** A solution of 5,6-dibromoisobenzofuran-1,3-dione (100 mg, 0.33 mmol) and 3-butyn-1-amine (27 µL, 0.33 mmol) in EtOH (5 mL) was irradiated under microwave conditions at 100 °C for 4 h. Compound **8b** was obtained as a white solid isolated by filtration (57.8 mg, 50%). m.p. 190–192 °C ^1^H RMN (400 MHz, CDCl_3_) δ 8.10 (s, 2H, Ar*H*), 3.87 (t, *J* = 6.9 Hz, 2H, NC*H_2_*), 2.61 (td, *J* = 6.9, 2.5 Hz, 2H, CC*H_2_*), 1.95 (t, *J* = 2.4 Hz, 1H, C*H*).

**4,5,6,7-tetrabromo-2- (but-3-yn-1-yl)isoindoline-1,3-dione (8c):** Et_3_N (450 µL, 3.24 mmol) was added to a solution of tetrabromophthalic anhydride (600 mg, 1.3 mmol) and 3-butyn-1-amine hydrochloride (172 mg, 1.62 mmol) in EtOH (5 mL). The mixture was irradiated under microwave conditions at 100 °C for 2 h. Compound **8c** was obtained as a white solid isolated by filtration (320 mg, 46%). m.p. 245–247 °C ^1^H RMN (400 MHz, CDCl_3_) δ 3.90 (t, *J* = 6.9 Hz, 2H, NC*H_2_*), 2.63 (td, *J* = 6.9, 2.4 Hz, 2H, CC*H_2_*), 1.97 (t, *J* = 2.3 Hz, 1H, C*H*).

**5-bromo-2-(but-3-yn-1-yl)isoindoline-1,3-dione (8d):** Et_3_N (821 µL, 5 mmol) was added to a solution of 4-bromophthalic anhydride (454 mg, 2 mmol) and 3-butyn-1-amine hydrochloride (253 mg, 2.4 mmol) in EtOH (5 mL). The mixture was irradiated under microwave conditions at 100 °C for 4 h. Compound **8d** was obtained as a white solid, which was isolated by filtration (160 mg, 29%). m.p. 127–129 °C ^1^H RMN (400 MHz, CDCl_3_) δ 8.00 (s, 1H, Ar*H*), 7.87 (d, *J* = 7.9 Hz, 1H, Ar*H*), 7.73 (d, *J* = 7.9 Hz, 1H, Ar*H*), 3.88 (t, *J* = 7.0 Hz, 2H, NC*H_2_*), 2.61 (td, *J* = 6.9, 2.5 Hz, 2H, CC*H_2_*), 1.96 (t, *J* = 2.4 Hz, 1H, C*H*). ^13^C RMN (101 MHz, CDCl_3_) δ 167.2, 166.7, 137.1, 133.6, 130.5, 129.0, 126.8, 124.8, 80.1, 70.4, 36.7, 18.3. **IR** (KBr) 1709 cm^−1^. MS (ESI) m/z 278.0 [M + H]^+^.

**2-(but-3-yn-1-yl)-5-methylisoindoline-1,3-dione (8e):** A solution of 4-methylphthalic anhydride (100 mg, 0.62 mmol) and 4-bromo-1-butyne (44 μL, 0.54 mmol) in EtOH (5 mL) was irradiated under microwave conditions at 100 °C for 3 h. The solvent was removed under vacuum, and the crude was solved up in EtOAc, washed with brine, dried over MgSO_4_, filtered, and evaporated. The crude was purified by column chromatography on silica gel using hexane-EtOAc (9.5:0.5) as eluent to give **8e** (64 mg, 49%) as a white solid. m.p. 114–115 °C ^1^H RMN (400 MHz, DMSO) δ 7.77 (d, *J* = 7.6 Hz, 1H, ArH), 7.71 (s, 1H, ArH), 7.65 (d*, J* = 7.8 Hz, 1H, ArH), 3.70 (t, *J* = 7.0 Hz, 2H, *n*-CH_2_), 2.81 (t, *J* = 2.5 Hz, 1H, CH), 2.54 (td, *J* = 6.9, 2.6 Hz, 2H, *n*-CH_2_-C*H_2_*), 2.48 (s, 3H, CH_3_). ^13^C NMR (101 MHz, DMSO-d_6_) δ 167.7, 167.6, 145.5, 134.9, 131.9, 128.9, 123.6, 123.1, 80.9, 72.8, 36.1, 21.4, 17.4. IR (KBr): 3284.2, 1758.1, 1704.6 cm^−1^.

**2-(but-3-yn-1-yl)-2H-benzo[d][1–3]triazole (6a) and 1-(but-3-yn-1-yl)-3a,7a-dihydro-1H-benzo[d][1–3]triazole (7a):** A suspension of benzotriazole (500 mg, 4.2 mmol) and K_2_CO_3_ (5.2 g, 38 mmol) in ACN (30 mL) was stirred for 5 min. Then, 4-bromobut-1-yne (1.23 mL, 12.6 mmol) was added and the reaction was left under argon at RT for 24 h. The solvent was removed under vacuum, and the crude was solved up in DCM, washed with brine and H_2_O, dried over MgSO_4_, filtered, and evaporated. The crude was purified by column chromatography on silica gel using hexane-EtOAc (9:1) as eluent to give **6a** (400 mg, 56%) as a light-yellow solid and **7a** (260 mg, 36%) as a beige solid. For **6a**: m.p. 73–75 °C ^1^H NMR (400 MHz, CDCl_3_) δ 7.96–7.66 (m, 2H, ArH), 7.47–7.28 (m, 2H, ArH), 4.86 (t, *J* = 7.3 Hz, 2H, NC*H_2_*), 3.01 (td, *J* = 7.3, 2.7 Hz, 2H, C*H_2_*C), 2.03 (t, *J*= 2.7 Hz, 1H, CC*H*). ^13^C NMR (100 MHz, CDCl_3_) δ 144.5, 126.5, 118.1, 79.4, 71.1, 54.8, 20.1. For **7a:** m.p. 74–76 °C. ^1^H NMR (400 MHz, CDCl_3_) δ 7.90 (d, *J* = 8.4 Hz, 1H, ArH), 7.46 (d, *J* = 8.3 Hz 1H, ArH), 7.34 (t, *J*= 7.6 Hz, 1H, ArH), 7.21 (t, *J* = 7.6 Hz, 1H, ArH), 4.65 (t, *J* = 7.0 Hz, 2H, NC*H_2_*), 2.76 (td, *J* = 7.0, 2.6 Hz, 2H, C*H_2_*C), 1.88 (t, *J* = 2.6 Hz, 1H, CC*H*). ^13^C NMR (100 MHz, CDCl_3_) δ 145.9, 133.1, 127.4, 123.9, 119.9, 109.5, 79.7, 71.5, 46.6, 20.2.

**(2*(R)-*2-(4-(4-(2-(2*H*-benzo[*d*][1–3]triazol-2-yl)ethyl)-1*H*-1,2,3-triazol-1-yl)phenylsulfonamido)-*N*-hydroxy-3-phenylpropanamide (9a)**

Method 1A was followed for the click reaction: From **3** (201 mg, 0.45 mmol) and **6a** (100 mg, 0.58 mmol), and after purification by column chromatography (hexane:EtOAc 7:3) followed by recrystallization from hexane-EtOAc, **OTHP-9a** (64 mg, 23%) was obtained as a white solid and as a mixture of diastereomers a:b (45:55). m.p. 170–171 °C. ^1^H NMR (400 MHz, CDCl_3_) δ 9.38 (brs, diastereomer a, 0.45H, NH), 9.23 (brs, diastereomer b, 0.55H, NH), 7.92–7.79 (m, 2H, ArH), 7.71–7.60 (m, 5H, CH-triazole), 7.41–7.32 (m, 2H, ArH), 7.17–6.99 (m, 5H, ArH), 5.84–5.79 (m, isomers a + b, 1H, N*H*CH), 5.15 (t, *J* = 6.9 Hz, 2H, NC*H_2_*), 4.75–4.71 (2x brs, isomers a + b, 1H, OCH), 3.97–3.92 (m, 1H), 3.86–3.80 (m, 1H), 3.70 (t, *J* = 6.8 Hz, 2H, NCH_2_C*H_2_*), 3.52–3,48 (m, 1H), 3.07–3.01 (m, 1H, C*H*HPh), 2.96–2.86 (m, 1H, CH*H*Ph), 1.75–1.48 (m, 6H, 3CH_2_).

This compound was deprotected following Method 2A. From **OTHP-9a** (68 mg, 0.11 mmol), compound **9a** (30 mg, 52%) was obtained as a white solid. m.p. 189 °C (decomp.). ^1^H NMR (400 MHz, DMSO) δ 10.66 (d, *J* = 1.5 Hz, 1H, N*H*OH), 8.86 (d, *J* = 1.6 Hz, 1H, NHO*H*), 8.70 (s, 1H, C*H*-triazole), 8.42 (d, *J* = 8.9 Hz, 1H, N*H*CH), 7.96–7.88 (m, 2H, ArH), 7.83 (d, *J* = 8.8 Hz, 2H, ArH), 7.65 (d, *J* = 8.7 Hz, 2H, ArH), 7.45–7.37 (m, 2H, ArH), 7.11–7.03 (m, 4H, ArH), 7.00–6.95 (m, 1H, ArH), 5.14 (t, *J* = 7.1 Hz, 2H, NC*H_2_*CH_2_), 3.84–3.78 (m, 1H, NHC*H*), 3.57 (t, *J* = 7.0 Hz, 2H, NCH_2_C*H_2_*), 2.77 (dd, *J* = 13.6, 5.5 Hz, 1H, C*H*HPh), 2.63 (dd, *J* = 13.7, 9.3 Hz, 1H, CH*H*Ph). ^13^C NMR (101 MHz, DMSO) δ 166.8, 144.5, 143.6, 140.7, 138.5, 136.8, 129.1, 128.0, 127.9, 126.4, 126.2, 121.2, 119.7, 117.8, 55.6, 55.1, 38.4, 25.8. HPLC purity 95%. MS (ESI) m/z 531.0 [M-H]^−^.

***(R*)-2-(4-(4-(2-(2*H*-benzo[*d*][1–3]triazol-2-yl)ethyl)-1*H*-1,2,3-triazol-1-yl)phenylsulfonamido)-3-phenylpropanoic acid (9b)**

Method 1A was followed for the click reaction: From (*R*)-**4** [34] (110 mg, 0.31 mmol) and **6a** (70.7 mg, 0.41 mmol), and after column chromatography (from pure DCM to DCM-MeOH 95:5), compound **9b** (85 mg 53%) was obtained as a white solid. m.p. 246 °C (decomp.). ^1^H NMR (400 MHz, DMSO) δ 12.80 (s, 1H, COO*H*), 8.72 (s, 1H, C*H* triazole), 8.35 (brs, 1H, N*H*CH), 7.98–7.89 (m, 2H, Ar*H*), 7.87 (d, *J* = 8.7 Hz, 2H, Ar*H*), 7.68 (d, *J* = 8.7 Hz, 2H, Ar*H*), 7.47–7.35 (m, 2H, Ar*H*), 7.17–7.07 (m, 4H, Ar*H*), 7.07–6.94 (m, 1H, ArH), 5.14 (t, *J* = 7.1 Hz, 2H, NC*H_2_*CH_2_), 3.86 (brs, 1H, NHCH), 3.55 (t, *J* = 7.0 Hz, 2H, NCH_2_C*H_2_*), 2.94 (dd, *J* = 13.7, 5.1 Hz, 1H, 1/2CH_2_Ph), 2.74 (dd, *J* = 13.6, 9.0 Hz, 1H, 1/2CH_2_Ph). 13C NMR (101 MHz, DMSO) δ 172.7, 145.0, 144.2, 141.0, 139.1, 137.4, 129.7, 128.6, 128.5, 126.9, 126.8, 121.7, 120.2, 118.3, 58.1, 55.6, 38.2, 26.3. HPLC purity 94%. MS (ESI) m/z 518.1 [M + H]^+^.

**(*S*)-2-(4-(4-(2-(2*H*-benzo[*d*][1–3]triazol-2-yl)ethyl)-1*H*-1,2,3-triazol-1-yl)phenylsulfonamido)-3-phenylpropanoic acid (9c)**

Method 1A was followed for the click reaction: From (S)-4 (75 mg, 0221 mmol) and **6a** (44.5 mg, 0.26 mmol), and after purification by column chromatography (from pure DCM to DCM-MeOH 95:5), compound **9c** (94 mg, 77%) was obtained as a white solid. m.p. 227–228 °C. ^1^H NMR (400 MHz, DMSO) δ 8.72 (s, 1H, CH triazole), 7.94–7.89 (m, 2H, ArH), 7.88 (d, *J* = 8.7 Hz, 2H, ArH), 7.72 (d, *J* = 8.7 Hz, 2H, ArH), 7.46–7.37 (m, 2H, ArH), 7.16–7.08 (m, 4H, ArH), 7.06–7.00 (m, 1H, ArH), 5.14 (t, *J* = 7.1 Hz, 2H, NC*H_2_*CH_2_), 3.78–3.73 (m, 1H, NHCH), 3.56 (t, *J* = 7.1 Hz, 2H, NCH_2_C*H_2_*), 2.95 (dd, *J* = 13.5, 5.0 Hz, 1H, 1/2CH_2_Ph), 2.78 (dd, *J* = 13.6, 8.3 Hz, 1H, 1/2CH_2_Ph). ^13^C NMR (101 MHz, DMSO) δ 172.3, 144.6, 143.7, 140.5, 138.6, 137.0, 129.3, 128.1, 128.0, 126.4, 126.3, 121.2, 119.7, 117.9, 57.8, 55.2, 37.8, 25.8. HPLC purity 95%. MS (ESI) m/z 518.2 [M + H]^+^.

**(*R*)-2-(4-(4-(2-(perbromo-2*H*-benzo[*d*][1–3]triazol-2-yl)ethyl)-1*H*-1,2,3-triazol-1-yl)phenylsulfonamido)-3-phenylpropanoic acid (9d)**

Method 1A was followed for the click reaction: from (*R*)-4 (110 mg, 0.31 mmol) and alkyne **6b** (199 mg, 0.41 mmol), and after purification by column chromatography (from pure DCM to DCM-MeOH 95:5), compound **9d** (64 mg, 27%) was obtained as a white solid. m.p. 127–130 °C ^1^H NMR (400 MHz, DMSO) δ 12.83 (s, 1H, COOH), 8.69 (s, 1H, CH triazole), 8.45 (brs, 1H, NHCH), 7.85 (d, *J* = 8.8 Hz, 2H, ArH), 7.69 (d, *J* = 8.7 Hz, 2H, ArH), 7.15–7.08 (m, 4H, ArH), 7.06–6.99 (m, 1H, ArH), 5.18 (t, *J* = 7.0 Hz, 2H, NC*H_2_*CH_2_), 3.93–3.88 (m, NHC*H*), 3.57 (t, *J* = 7.0 Hz, 2H, NCH_2_C*H_2_*), 2.96 (dd, *J* = 13.7, 5.1 Hz, 1H, 1/2C*H_2_*Ph), 2.72 (dd, *J* = 13.7, 9.5 Hz, 1H, 1/2C*H_2_*Ph). HPLC purity 94%. MS (ESI) m/z 831.8 [M-H]^−^.

**4-((4-(4-(2-(2*H*-benzo[d][1–3]triazol-2-yl)ethyl)-1*H*-1,2,3-triazol-1-yl)phenyl)sulfonyl)-*N*-hydroxytetrahydro-2*H*-pyran-4-carboxamide (9e)**

Method 1A was followed for the click reaction: From **5** [35] (200 mg, 0.49 mmol) and **6a** (100 mg, 0.59 mmol), and after purification by column chromatography (DCM-MeOH (1.5% MeOH)) OTHP-9e (190 mg, 67%) was obtained as a white solid. m.p. 199.7–201.4 °C. ^1^H NMR (400 MHz, CDCl_3_) δ 9.37 (s, 1H, NH), 8.01–7.98 (m, 2H, ArH), 7.88–7.85 (m, 4H, ArH), 7.68 (s, 1H, C*H*-triazole), 7.42–7.37 (m, 2H, ArH), 5.15 (t, *J* = 6.9 Hz, 2H, NC*H_2_*), 5.01 (t, *J* = 2.5 Hz, 1H, OCH), 4.03–3.96 (m, 3H), 3.74–3.69 (m, 3H), 3.49 (td, 2H, *J* = 12.0, 4,6 Hz, 2H, OC*H*H), 2.31 (td, 2H, *J* = 12.3, 4,7 Hz, 2H, C*H*H), 2.07 (d, 2H, *J* = 13.2 Hz, 2H, C*H*H) 1.89–1.61 (m, 6H, 3CH_2_). ^13^C NMR (100 MHz, CDCl_3_ + acetone-d_6_) δ 163.1, 145.1, 144.2, 133.6, 132.1, 126.4, 120.0, 117.9, 101.8, 70.1, 64.2, 62.2, 55.2, 30.2, 28.2, 27.8, 26.2, 24.9, 18.1. IR (KBr): 3462, 3168, 2952, 1703, 1683, 1597, 1507 cm^−1^.

This compound was deprotected following Method 2A. From **OTHP**-**9e** (176 mg, 0.30 mmol) and after column chromatography (from DCM:MeOH 100:0 to 97.5:2.5) **9e** (84 mg, 56%) was obtained as a white solid. m.p. 219.4–220.8 °C. ^1^H NMR (400 MHz, DMSO) δ 11.04 (brs, 1H), 9.24 (brs, 1H), 8.83 (s, 1H), 8.11 (d, *J* = 8.8 Hz, 2H), 7.92–7.90 (m, 4H), 7.45–7.40 (m, 2H), 5.14 (t, *J* = 7.1 Hz, 2H), 3.89 (dd, *J* = 11.6, 3.7 Hz, 2H), 3.57 (t, *J* = 7.1 Hz, 2H), 3.18–3.13 (m, 2H), 2.23 (d, *J* = 13.0 Hz, 2H), 1.96 (td, *J* = 13.0, 4.6 Hz, 2H). ^13^C NMR (101 MHz, DMSO) δ 160.3, 144.7, 143.6, 140.4, 133.8, 132.1, 126.4, 121.4, 119.7, 117.8, 69.5, 63.8, 55.1, 27.2, 25.7. IR (KBr): 3305, 3140, 2924, 1668, 1589, 1507 cm^−1^ HPLC purity >98%. MS (ESI) m/z 498.1 [M + H]^+^.

**(*R*)-2-((4-(4-(2-(1,3-dioxoisoindolin-2-yl)ethyl)-1*H*-1,2,3-triazol-1-yl)phenyl)sulfonamido)-*N*-hydroxy-3-phenylpropanamide (10a)**

Method 1A was followed for the click reaction: From **3** (150 mg, 0.34 mmol) and *N*- (3-butynyl)phthalimide (80.5 mg, 0.4 mmol), and after column chromatography (from DCM:MeOH 100:0 to 98:2), **OTHP-10a** (146.3 mg, 68%) was obtained as a white solid and as a mixture of diastereomers a:b (45:55). m.p. 177 °C (decomp.). ^1^H NMR (400 MHz, CDCl_3_) δ 8.85 (s, 0.45H, NH diastereomer a), 8.67 (s, 0.55H, NH diastereomer b), 7.98 (s, 1H, C*H*-triazole), 7.85–7.71 (m, 8H, ArH), 7.19–7.16 (m, 3H, ArH), 7.00 (brs, 2H, ArH), 5.23–5.20 (m, 1H, SO_2_NH), 4.80 (s, 0.5H, O-C*H-O* diastereomer a or b), 4.72 (s, 0.5H, O-C*H-*O diastereomer a or b), 4.11 (t, *J* = 7.04, 2H, NCH_2_) 3.88–3.77 (m, 2H, OC*H*H, SO_2_NHC*H*), 3.58–3.54 (m, 1H, OCH*H*), 3.27 (t, *J* = 6.8 Hz, 2H, CH_2_), 3.06 (dd, *J* = 14.3 Hz, 6.4 Hz, 1H, CH*H*-Ph), 2.97–2.91 (m, 1H, CH*H*-Ph), 1.75–1.55 (m, 6H, 3CH_2_ THP). IR (KBr): 3270.3, 2950.6, 1712.6 cm^−1^.

This compound was deprotected following Method 2A: From **OTHP**-**10a** (121.5 mg, 0.19 mmol) and after column chromatography (from DCM:MeOH 100:0 to 97:3), **10a** (63 mg, 60%) was obtained as a white solid. m.p. 217–219 °C. ^1^H NMR (400 MHz, DMSO) δ 10.68 (brs, 1H, NH), 8.88 (s, 1H, OH), 8.80 (s, 1H, CH-triazole), 8.43 (brs, 1H, SO_2_NH), 7.88–7.82 (m, 6H, ArH), 7.66 (d, *J* = 8.6 Hz, 2H, ArH), 7.12–7.01 (m, 5H, ArH), 3.92 (t, *J* = 7.1 Hz, 2H, NCH_2_), 3.82 (brs, 1H, NH-C*H*), 3.10 (t, *J* = 7.1 Hz, 2H, CH_2_), 2.78 (dd, *J* = 13.7, 5.4 Hz, 1H, C*H*H-Ph), 2.64 (dd, *J* = 13.6, 9.4 Hz, 1H, CH*H*-Ph). ^13^C NMR (101 MHz, DMSO) δ 167.8, 166.8, 145.1, 140.6, 138.7, 136.9, 134.4, 131.7, 128.04, 127.9, 126.3, 123.1, 121.1, 119.6, 55.7, 38.4, 37.3, 24.1. IR (KBr): 3271, 2931, 1772, 1715 cm^−1^. HPLC purity 98%. MS (ESI) m/z 561 [M + H]^+^.

**(*R*)-2-((4-(4-(2-(5,6-dibromo-1,3-dioxoisoindolin-2-yl)ethyl)-1*H-*1,2,3-triazol-1-yl)phenyl)sulfonamido)-*N*-hydroxy-3-phenylpropanamide (10b)**

Method 1B was followed for the click reaction: From **3** (56,3 mg, 0.13 mmol) and 8b (49,6 mg, 0.14 mmol), and after column chromatography (from DCM:MeOH 100:0 to 98.5:1.5) **OTHP-10b** (30 mg, 29%) was obtained as a white solid and as a mixture of diastereomers a:b (57:43). m.p. 187–189 °C. ^1^H RMN (400 MHz, DMSO) δ 11.30 (s, 0.57H, diastereomer a, CON*H*), 11.22 (s, 0.43H, diastereomer b, CON*H*), 8.79 (s, 1H, C*H*-triazole), 8.51 (d, *J* = 9.2 Hz, 0.57H, diastereomer a, SO_2_N*H*), 8.46 (d, *J* = 8.8 Hz, 0.43H, diastereomer b, SO_2_N*H*), 8.26 (s, 2H, Ar*H*), 7.91–7.87 (m, 2H, Ar*H*), 7.72 (d, *J* = 8.6 Hz, 2H, Ar*H*), 7.22–7.03 (m, 5H, Ar*H*), 4.56 (brs, 0.43H, diastereomer b, OC*H*), 4.34 (brs, 0.57H, diastereomer a, OC*H*), 3.90 (t, *J* = 7.0 Hz, 2H, NC*H*_2_), 3.83–3.74 (m, 2H, COC*H* y OCH*H*), 3.48–3.36 (m, 1H, OC*H*H), 3.08 (t, *J* = 7.1 Hz, 2H, triazole-C*H_2_*), 2.79 (“dd”, 1H, Ph-C*H*H), 2.71–2.62 (m, 1H, Ph-CH*H*), 1.63–1.30 (m, 6H, 3C*H_2_*). IR (KBr) 3260, 1717 cm^−1^.

This compound was deprotected following Method 2B: From **OTHP**-**10b** (30 mg, 0.04 mmol) and after column chromatography (from DCM:MeOH 98:2 to 90:10) followed by recrystallization from EtOH:H_2_O, **10b** (19.5 mg, 69%) was obtained as a white solid. m.p. 154–156 °C. ^1^H NMR (400 MHz, DMSO) δ 10.68 (brs, 1H, CON*H*), 8.88 (brs, 1H, O*H*), 8.78 (s, 1H, C*H* triazole), 8.43 (brs, 1H, SO_2_N*H*), 8.26 (s, 2H, Ar*H*), 7.86 (d, *J* = 8.2 Hz, 2H, Ar*H*), 7.66 (d, *J* = 8.2 Hz, 2H, Ar*H*), 7.15–6.98 (m, 5H, Ar*H*), 3.90 (t, *J* = 7.1 Hz, 2H, NC*H_2_*), 3.85–3.78 (m, 1H, C*H*), 3.08 (t, *J* = 6.9 Hz, 2H, triazol-C*H_2_*), 2.78 (dd, *J* = 13.7, 5.8 Hz, 1H, Ph-C*H*H), 2.70–2.58 (m, 1H, Ph-CH*H*). ^13^C RMN (101 MHz, DMSO) δ 167.3, 166.6, 145.4, 141.1, 139.1, 137.3, 132.5, 131.0, 129.6, 128.6, 128.5, 128.4, 126.8, 121.6, 120.1, 56.1, 38.9, 38.1, 24.3. IR (KBr) 1717 cm^−1^ HPLC purity 99%. MS (ESI) m/z 719.0 [M + H]^+^.

**(*R*)-*N*-hydroxy-3-phenyl-2-((4-(4-(2-(4,5,6,7-tetrabromo-1,3-dioxoisoindolin-2-yl)ethyl)-1*H*-1,2,3-triazol-1-yl)phenyl)sulfonamido)propenamide 10c**

Method 1B was followed for the click reaction: From **3** (200 mg, 0.45 mmol) and **8c** (300 mg, 0.58 mmol), after column chromatography (from DCM:MeOH 100:0 to 98.5:1.5), followed by recrystallization from hexane-EtOAc, **OTHP**-**10c** (185 mg, 43%) was obtained as a white solid and as a mixture of diastereomers a:b (50:50). m.p. 195–197 °C. ^1^H NMR (400 MHz, DMSO) δ 11.27 (s, 0.5H, diastereomer a, CON*H*), 11.19 (s, 0.5H, diastereomer *b*, CON*H*), 8.79 (s, 1H, C*H* triazole), 8.48 (d, *J* = 9.1 Hz, 0.5H, diastereomer a or b SO_2_N*H*), 8.43 (d, *J* = 8.7 Hz, 0.5H, diastereomer a or b, SO_2_N*H*), 7.90 (d, *J* = 8.6 Hz, 2H, Ar*H*), 7.73 (d, *J* = 8.2 Hz, 2H, Ar*H*), 7.18–7.08 (m, 5H, Ar*H*), 4.57 (s, 0.5H, diastereomer a or b, OC*H*), 4.37 (s, 0.5H, diastereomer a or b, OC*H*), 3.91 (t, *J* = 7.0 Hz, 2H, NC*H_2_*), 3.85–3.75 (m, 2H, COC*H* y OCH*H*), 3.49–3.34 (m, 1H, OC*H*H), 3.09 (“t”, 2H, C*H_2_*), 2.84–2.76 (m, 1H, Ph-C*H*H), 2.72–2.63 (m, 1H, Ph-CH*H*), 1.37–1.62 (m, 6H, 3C*H_2_*). IR (KBr) 2951, 1713 cm^−1^.

This compound was deprotected following Method 2B: From **OTHP-10c** (160 mg, 0.17 mmol) and after column chromatography (from DCM:MeOH 98:2 to 90:10) followed by recrystallization from EtOH:H_2_O, **10c** (45 mg, 31%) was obtained as a white solid. m.p. 165 °C (decomp.). ^1^H NMR (400 MHz, DMSO) δ 10.67 (s, 1H, CON*H*), 8.88 (s, 1H, O*H*), 8.79 (s, 1H, C*H* triazole), 8.43 (d, *J* = 7.4 Hz, 1H, SO_2_N*H*), 7.86 (d, *J* = 8.4 Hz, 2H, Ar*H*), 7.67 (d, *J* = 8.5 Hz, 2H, Ar*H*), 7.15–7.05 (m, 5H, Ar*H*), 3.91 (t, *J* = 7.0 Hz, 2H, NC*H_2_*), 3.86–3.78 (m, 1H, C*H*), 3.08 (t, *J* = 7.1 Hz, 2H, triazole-C*H_2_*), 2.78 (dd, *J* = 13.7, 5.5 Hz, 1H, Ph-C*H*H), 2.69–2.59 (m, 1H, Ph-CH*H*). ^13^C RMN (101 MHz, DMSO) δ 167.3, 164.1, 145.3, 141.2, 139.1, 137.3, 136.9, 131.6, 129.6, 128.5, 128.4, 126.8, 121.7, 121.0, 120.2, 56.1, 38.9, 38.5, 24.2. IR (KBr) 3287, 1717 cm^−1^ HPLC purity 95%. MS (ESI) m/z 876.9 [M + H]^+^.

**(*R*)-2-((4-(4-(2-(5-bromo-1,3-dioxoisoindolin-2-yl)ethyl)-1*H*-1,2,3-triazol-1-yl)phenyl)sulfonamido)-*N*-hydroxy-3-phenylpropanamide (10d)**

Method 1B was followed for the click reaction: From **3** (100 mg, 0.22 mmol) and **8d** (75 mg, 0.27 mmol), and after column chromatography (from DCM:MeOH 100:0 to 97.5:2.5), **OTHP**-**10d** (160.3 mg, quantitative yield) was obtained as a white solid and as a mixture of diastereomers a:b (50:50). m.p. 104 °C (decomp.). ^1^H RMN (400 MHz, DMSO) δ 11.29 (s, diastereomer a, *0*.5H, CON*H*), 11.21 (s, diastereomer b, 0.5H, CON*H*), 8.80 (s, 1H, diastereomer *a or b,* C*H* triazole), 8.79 (s, 1H, diastereomer *a or b,* C*H* triazole), 8.50 (d, *J* = 9.2 Hz, 0.5H, diastereomer a or b,SO_2_N*H*), 8.45 (d, *J* = 8.9 Hz, 0.5H, diastereomer a or b SO_2_N*H*), 8.07 (d, *J* = 0.8 Hz, 1H, Ar*H*), 8.04 (dd, *J* = 7.9, 1.1 Hz, 0.5H, diastereomer a or b, Ar*H*), 8.03 (dd, *J* = 7.9, 1.6 Hz, 0.5H, diastereomer a or b, Ar*H*), 7.91–7.88 (m, 2H, Ar*H*), 7.80 (d, *J* = 7.9 Hz, 1H, Ar*H*), 7.71 (d, *J* = 8.7 Hz, 2H, Ar*H*), 7.21–7.02 (m, 5H, Ar*H*), 4.56 (“t”, 0.5H, diastereomer a or b, OC*H*), 4.34 (“t”, 0.5H, diastereomer a or b*,* OC*H*), 3.90 (t, *J* = 7.1 Hz, 2H, NC*H_2_*), 3.90–3.75 (m, 2H, COC*H* + OC*H*H), 3.50–3.37 (m, 1H, OCH*H*), 3.09 (t, *J* = 7.1 Hz, 2H, triazole-C*H_2_*), 2.85–2.75 (m, 1H, Ph-C*H*H), 2.71–2.62 (m, 1H, Ph-CH*H*), 1.62–1.34 (m, 6H, 3C*H_2_*). IR (KBr) 1713 cm^−1^.

This compound was deprotected following Method 2B: From **OTHP**-**10d** (160 mg, 0.22 mmol) and after column chromatography (from DCM:MeOH 98:2 to 90:10) followed by recrystallization from EtOH:H_2_O, **10d** (78 mg, 55%) was obtained as a white solid. m.p. 149–152 °C. ^1^H NMR (400 MHz, DMSO) ^1^H RMN (400 MHz, DMSO) δ 10.68 (brs, 1H, CON*H*), 8.88 (brs, 1H, O*H*), 8.79 (s, 1H, C*H* triazole), 8.43 (brs, 1H, SO_2_N*H*), 8.08 (s, 1H, Ar*H*), 8.04 (d, *J* = 8.0 Hz, 1H, Ar*H*), 7.86 (d, *J* = 8.5 Hz, 2H, Ar*H*), 7.80 (d, *J* = 7.9 Hz, 1H, Ar*H*), 7.66 (d, *J* = 8.6 Hz, 2H, Ar*H*), 7.15–7.00 (m, 5H, Ar*H*), 3.91 (t, *J* = 7.0 Hz, 2H, NC*H_2_*), 3.83–3.80 (m, 1H, CO-C*H*), 3.09 (t, *J* = 6.9 Hz, 2H, triazole-C*H_2_*), 2.78 (dd, *J* = 13.5, 5.2 Hz, 1H, Ph-C*H*H), 2.70–2.60 (m, 1H, Ph-CH*H*). ^13^C NMR (101 MHz, DMSO) δ 167.7, 167.4, 167.1, 145.6, 140.8, 139.1, 137.6, 137.1, 134.0, 130.9, 130.0, 128.5, 128.5, 128.4, 126.8, 126.4, 125.4, 121.5, 120.2, 56.1, 38.7, 38.0, 24.2. IR (KBr) 1709 cm^−1^ HPLC purity 97%. MS (ESI) m/z 639.1 [M + H]^+^.

**(*R*)-*N*-hydroxy-2-((4-(4-(2-(5-methyl-1,3-dioxoisoindolin-2-yl)ethyl)-1*H*-1,2,3-triazol-1-yl)phenyl)sulfonamido)-3-phenylpropanamide 10e**

Method 1A was followed for the click reaction: From azide **3** (104.5 mg, 0.23 mmol) and alkyne **8e** (60 mg, 0.28 mmol), and after column chromatography (from DCM:MeOH 100:0 to 98:2), **OTHP-10e** (159 mg, 97%) was obtained as a white solid and as a mixture of diastereomers a:b (45:55) m.p. 143–145 °C. ^1^H NMR (400 MHz, DMSO) δ 11.29 (s, 0.45H, NH diastereomer a), 11.20 (s, 0.55H, NH diastereomer b), 8.79 (s, 1H, CH-triazole), 8.51–8.44 (m, 1H, SO_2_NH diastereomer a + b) 7.91–7.88 (m, 2H, ArH), 7.75–7.62 (m, 5H, ArH), 7.15–7.08 (m, 5H, ArH), 4.56 (s, 0.5H, O-CH*-*O diastereomer a or b), 4.34 (s, 0.5H, O-CH-O diastereomer a or b), 3.91–3.76 (m, 4H), 3.46–3.38 (m, 1H partially under H_2_O signal), 3.08 (“t”, *J* = 6.2 Hz, 2H, CH_2_), 2.82–2.76 (m, 1H, CH*H*-Ph), 2.70–2.64 (m, 1H, C*H*H-Ph), 2.47 (s, 3H, CH_3_ partially under DMSO peak), 1.57–1.35 (m, 6H, 3CH_2_ THP). IR (KBr): 3272, 2947, 1770, 1705 cm^−1^.

This compound was deprotected following Method 2A: From **OTHP**-**10e** (159 mg, 0.24 mmol) and after column chromatography (from DCM:MeOH 100:0 to 90:10), **10e** (31 mg, 23%) was obtained as a white solid. m.p. 170–172 °C. ^1^H NMR (400 MHz, DMSO) δ 10.69 (brs, 1H, NH), 8.89 (s, 1H), 8.79 (s, 1H), 8.44 (brs, 1H, SO_2_NH), 7.86 (d, *J* = 8.5 Hz, 2H, ArH), 7.75 (d, *J* = 7.6 Hz, 1H, ArH), 7.70 (s, 1H, ArH), 7.67–7.62 (m, 3H, ArH), 7.11–7.02 (m, 5H, ArH), 3.89 (t, *J* = 7.1 Hz, 2H, NCH_2_), 3.81 (brs, 1H, NH-C*H*), 3.08 (t, *J* = 7.1 Hz, 2H, CH_2_), 2.78 (dd, *J* = 13.5, 5.5 Hz, 1H, C*H*H-Ph), 2.64 (dd, *J* = 13.6, 9.4 Hz, 1H, CH*H*-Ph), 2.54 (s, 3H, CH_3_). ^13^C NMR (101 MHz, DMSO) δ 168.3, 168.2, 167.3, 145.8, 145.5, 141.1, 139.1, 137.3, 135.2, 132.5, 129.6, 129.5, 128.5, 128.4, 126.7, 124.0, 123.5, 121.6, 120.1, 56.1, 38.9, 37.7, 24.5, 21.8. IR (KBr): 3259, 1701, 1655 cm^−1^. HPLC purity 96% MS (ESI) m/z 575.1 [M+H]^+^.

### 4.2. MMP Inhibition Assays

MMP activity measurements were performed using an MMP Inhibitor Profiling Kit purchased from Enzo Life Science International, Inc. (Farmingdale, NY, USA), and following the manufacturer’s protocol with slight modifications. Proteolytic activity was measured using a thiopeptide substrate (Ac-PLG-[2-mercapto-4-methylpentanoyl]-LG-OC_2_H_5_) where the MMP cleavage site peptide bond has been replaced by a thioester bond. Hydrolysis of this bond by MMPs produces a sulfhydryl group that reacts with DTNB to form 2-nitro-5-thiobenzoic acid, which was detected by its absorbance at 414 nm (microplate photometer 90 Thermo Scientific Multiscan FC).

Enzyme reactions were carried out at 37 °C in a 100 μL final volume of solutions, where the catalytic domains of the corresponding MMP were incubated in triplicate with at least seven concentrations of inhibitors. The assay buffer contained the following components: 50 mM HEPES, 10 mM CaCl_2_ 95, 0.05% Brij-35, and 1 mM DTNB at pH 7.5. After addition of the substrate, the increase of absorbance was recorded in 1 min time intervals for 30 min. Data were plotted as OD versus time for each sample to obtain the reaction velocity (V) in OD per min. The percentage of residual activity for each compound was calculated using the following formula: % of remaining activity = (V in the presence of inhibitor/Vcontrol) × 100. An inhibitor, NNGH, was included as a prototypic control inhibitor. IC_50_ calculations in *MMP-13* and *MMP-2* for active compounds are shown in Supplementary Materials (Figure S9)

The concentration of the compound that provided 50% inhibition of enzymatic activity (IC_50_) was determined by using semi-logarithmic dose–response plots (Graph Pad Prism 5.0 for Windows, Graph Pad Software Inc., San Diego, CA, USA, 2007).

### 4.3. Cell Culture

The human osteosarcoma cell line MG-63 was kindly provided by Dr. A. Rodríguez de Gortazar (Universidad San Pablo-CEU, Madrid, Spain). Briefly, MG-63 cells were seeded in gelatin-coated plates in Dulbecco’s modified Eagle’s medium (DMEM, Biowest, Nuaillé, FR), supplemented with 10% (*v*/*v*) fetal bovine serum (Biowest) and penicillin/streptomycin (Sigma Aldrich, St. Louis, MO, USA), and maintained in a humidified incubator (37 °C and 5% CO_2_) for 24 h. The cells were then washed with phosphate-buffered saline (PBS) and serum-starved for 24 h. Thereafter, the cells were incubated in the presence of 0.5, 5, or 50 nM MY399 for 24 h. As **9a** was dissolved in dimethylsulfoxide (DMSO, Sigma, St. Louis, MO, USA), cells treated with DMSO were used as a control. After 24 h, supernatants were collected, centrifuged at 1500 rpm for 15 min, and stored at −80 °C until further use.

*MMP-13* activity was determined in MG-63 supernatants using the SensoLyte^®^ Plus 520 *MMP-13* assay kit (Anaspec, Fremont, CA, USA) as per the manufacturer’s guidelines. The results obtained were normalized to total protein content in the supernatants. Statistical analyses were performed using Graphpad (version 6.5). Normal distribution of the data was confirmed using the Kolmogorov–Smirnov test. Statistical significance was determined using one-way ANOVA with Bonferroni post-test, with *p* < 0.05 considered as statistically significant.

### 4.4. Thermodynamic Solubility (Shake Flask Method)

Compounds that showed activity in the enzymatic assay, **1**, **2**, **9a**, **9e**, **10a–e**, were added into 1.5 mL Eppendorf tubes prior to the addition of pH 7.4 phosphate buffer to obtain a nominal concentration of 1 mg/mL. The samples were sonicated for 10 min and shaken overnight at 800 rpm (FB15013 TopMix, Fischer Scientific, Waltham, MA, USA) at room temperature. A first phase separation of supernatant and undissolved solid was performed by centrifugation at 5000 rpm for 20 min (Biofuge primo R). The supernatant was then transferred into Eppendorf tubes for a second centrifugation. An adequate dilution was made using the particle-free supernatant and the concentration quantified by LC-HRMS using a 4-point calibration curve (Agilent 1260 Infinity II).

### 4.5. Recombinant Protein Production

A recombinant *MMP-13* construct containing the catalytic site (amino acid residues −104 to 274) was expressed from a modified version of the pET-22b (Novagen, Merck, (Darmstadt, Germany) expression vector containing a TEV cleavage site and a C-terminal 6xHis-tag in *Escherichia coli* BL21-DE3 pLys S (Stratagene, La Jolla, CA, USA) cells. The cells were grown at 37 °C until an absorbance of 0.6 at 600 nm was reached and then induced with 1 mM of isopropyl ß-D-1-thiogalactopyranoside (IPTG) for 4 h at 37 °C. The cells were harvested by centrifugation at 3500 rpm for 15 min at 4 °C. The drained pellet was either stored at -80 °C or subjected to cell disruption for immediate use. Cell pellets were harvested and resuspended in 100 mL of lysis buffer (20 mM Tris-HCl, pH 8.0, 300 mM NaCl and 6M urea). The suspension was incubated and shaken for 30 min at room temperature and subsequently sonicated (Microson Ultrasonic cell disruptor XL, Misonix) on ice 12 times, 15 s each, to break the cell wall and centrifuged at 9000 rpm for 20 min. The solubilized *MMP-13* recombinant protein was purified using metal affinity chromatography under denaturing conditions. The supernatant was mixed with 2.5 mL of Ni-NTA His Bind resin (Novagen) previously equilibrated with buffer (20 mM Tris-HCl, pH 8.0, 300 mM NaCl and 6 M urea), leaving the mixture supernatant-resin incubating and shaking for 1 h at room temperature. The column was washed with 50 mL of buffer and afterward with 50 mL of buffer supplemented with 10 and 30 mM imidazole, respectively. The protein bound to the resin was eluted with 3 fractions of 5 mL of buffer supplemented with 250, 500 mM, and 1 M imidazole, respectively. The purity of all fractions was assessed by SDS-PAGE, and those fractions free of contaminants were pooled and subjected to buffer exchange using a desalting PD MidiTrap G-25 column (GE Healthcare, Chicago, IL, USA), in order to remove imidazole and refold the protein. Desalting columns were equilibrated with 20 mM Tris-HCl, pH 8.0, 200 mM NaCl buffer. Finally, eluted fractions were dialyzed against 10 mM Tris-HCl, pH 7.4, 50 mM NaCl buffer. Protein aliquots at 5 uM were lyophilized and stored at −20 °C before their use in WaterLOGSY experiments.

### 4.6. NMR Experiments

All NMR experiments were performed using a Bruker UltrashieldTM Plus 500 MHz spectrometer equipped with a 5 mm TXI probe at 25 °C. For WaterLOGSY experiments, to 473 μL of 5 μM *MMP-13* catalytic domain with 100 μM CaCl_2_ and 100 μM ZnCl_2_, 50 μL of D_2_O, and 0.15 mM of ligand were added (from a 100 mM stock in DMSO-d6), in a protein/ligand ratio of 1:30, optimal for the WaterLOGSY experiments. For each sample, 1D ^1^H and WaterLOGSY experiments were conducted. A total of 8 K-points were used for a sweep width of 16 ppm in both experiments. For the 1D ^1^H experiments, 64 scans were accumulated. A total of 360 scans were accumulated for the WaterLOGSY experiment. Spectra were acquired and processed with Topspin 3.5 (Bruker Biospin), and MNova software (MestReNova, v8.1.2).

### 4.7. Molecular Modeling

To account for the flexibility of the Ω-loop, we selected several X-ray structures of the catalytic domain of *MMP-13* bound to various inhibitors (PDB codes 3KEK, 3WV1, 456C and 3TVC) [44,45,46,47] and the eleven conformations issued from the NMR-solved catalytic domain of *MMP-*2 (PDB code 1HOV) also bound to an inhibitor [48]. All fifteen proteins were prepared with the Protein Preparation Wizard module of the Schrödinger software, where the inhibitors and water molecules were eliminated [49,50]. The catalytic glutamic acid (Glu223 in *MMP-13* and Glu121 in *MMP-2*) is known to deprotonate different types of inhibitors depending on the type of Zinc Binding Group (ZBG), such as hydroxamic acid. Given the nature of the tested inhibitors, two sets of proteins with the two types of protonation of the catalytic glutamic acid were prepared. The induced-fit docking protocol of the Glide software was applied as indicated in the literature [51,52,53,54]. All compounds were docked in a 25Å sized box, with the center of the box located at a centroid between the Zn^2+^ and Tyr244 *MMP-13*/Tyr142 *MMP-2* [54]. Ligands were prepared using the LigPrep module of the Schrödinger suite, where metal coordination options were added for the calculation [54]. The hydroxamate form was used for the docking calculations using the protonated forms of Glu223/Glu121, whereas the carboxylate-based inhibitors were docked in the deprotonated forms of Glu223/Glu121.

## Figures and Tables

**Table 1:** IC_50_ inhibitory values, calculated logD and calculated and experimental logS for compounds **1, 2, 9,** and **10**.

Compound	*MMP*-13 IC_50_ (nM)	*MMP*-2 IC_50_ (nM)	clogD ^1^	Water Solubility LogS ^1,2^	Experimental Water Solubility LogS ^2,3^
**9a**	0.65 ± 0.30	3.42 ± 1.15	2.94	−3.71	−5.01
**9b**	>100	>100	0.32	0.00	n.d.
**9c**	>100	>100	0.32	0.00	n.d.
**9d**	>100	>100	3.39	−1.93	n.d.
**9e**	3.3 ± 2.1	10.3 ± 2.8	1.37	−2.72	−4.47
**10a**	6.9 ± 1.1	3.1 ± 0.9	2.37	−5.17	−5.57
**10b**	46 ± 21	15 ± 5	3.90	−6.36	−5.44
**10c**	61 ± 18	17 ± 4	5.44	−7.16	−6.85
**10d**	15 ± 4	14 ± 7	3.14	−5.81	−5.64
**10e**	9.2 ± 3.0	2.9 ± 1.1	2.88	−5.61	−5.08
**1**	3.70 ± 1.85	70	6.01	−5.85	−5.54
**2**	5.60 ± 2.66	100	4.44	−5.03	−5.62

^1^ Calculated with Chemicalize—Instant Cheminformatics Solutions. ^2^ logS scale: insoluble < −10 < poorly < −6 < moderately < −4 < soluble <−2 < very < 0 < highly. ^3^ Water solubility in PBS pH 7.4.

**Table 2:** IC_50_ inhibitory values for compounds **1** and **9a** in a panel of MMPs.

Enzyme	Compound 9a	Compound 1
*MMP-1*	>1000	>1000
*MMP-2*	3.42 ± 1.15	70
*MMP-3*	31 ± 8	500
*MMP-7*	47 ± 21	>1000
*MMP-8*	100 ± 19	>500
*MMP-9*	500 ± 74	>500
*MMP-10*	280 ± 22	434
*MMP-12*	3.7 ± 2.0	17.8
*MMP-13*	0.65 ± 0.30	3.7
*MMP-14*	>1000	>1000

## Data Availability

All data used in this study are already provided in the manuscript at the required section.

## Supplementary Materials

The following are available online at https://www.mdpi.com/article/10.3390/ijms22189976/s1.

## Abbreviations

ACN	acrylonitrile
DCM	dichloromethane
DMF	dimethylformamide
DMSO	dimethylsulfoxide
MW	microwave
PDB	protein data bank
PK	pharmacokinetic
RT	room temperature
THF	tetrahydrofuran
THP	tetrahydropyrane
TBTA	(Tris[(1-benzyl-1*H*-1,2,3-triazol-4-yl)methyl]amine)
TLC	thin layer chromatography
