# Supplementary material for: Design and Synthesis of Water-Soluble and Potent MMP-13 Inhibitors with Activity in Human Osteosarcoma Cells
Source: Int J Mol Sci. 2021 Sep 15;22(18):9976. doi: 10.3390/ijms22189976 (PMC8467962; doi:10.3390/ijms22189976)
Supplement: Supplementary file 1 [file ijms-22-09976-s001.zip › ijms-1311407-SI.pdf]

Supplementary Materials

Design and synthesis of water-soluble and potent MMP-13 inhibitors with activity in human osteosarcoma cells

Jose María Zapico,[1] Lourdes Acosta,[1] Myriam Pastor,[1] Loganathan Rangasamy,[1] Laura Márquez-Cantudo,[1] Claire Coderch,[1] Irene Ortín,[1] Maria Nicolau-Sanus,[2] Leonor Puchades-Carrasco,[2] Antonio Pineda-Lucena,[3] Alejandro Majali-Martinez,[1] Pilar Ramos,[1] Beatriz de Pascual-Teresa,[1]* Ana Ramos.[1]*

[1] Departamento de Química y Bioquímica, Facultad de Farmacia, Universidad San Pablo-CEU, CEU Universities, Urbanización Montepríncipe, 28925, Alcorcón, Madrid, Spain.

[2] Drug Discovery Unit, Instituto de Investigación Sanitaria La Fe, Valencia, Spain.

[3] Molecular Therapeutics Program, Centro de Investigación Médica Aplicada, Universidad de Navarra, Pamplona, Spain.

Table of contents

PART A. Calibration curves of compounds for solubility studies. 2

PART B. ^{1}H and ^{13}C NMR spectra. 3

PART C. Modeling 13

PART D. NMR studies 24

PART E. IC_{50} calculations 25

PART F. Purity by HPLC for the final compounds. 27

PART A. Calibration curves of compounds for solubility studies.

To calculate the solubility of all compounds that showed activity in the enzymatic assay (**1**, **2**, **9a**, **9e**, **10a-e**), 4-points calibration curves were made for each compound. Calibrations curves were obtained using four different concentrations of the corresponding compound (0.1 mg/mL, 0.01 mg/mL, 0.001 mg/mL and 0.0001 mg/mL). All samples were prepared in DMSO and measured in a LC-HRMS (Agilent 1260 Infinity II).

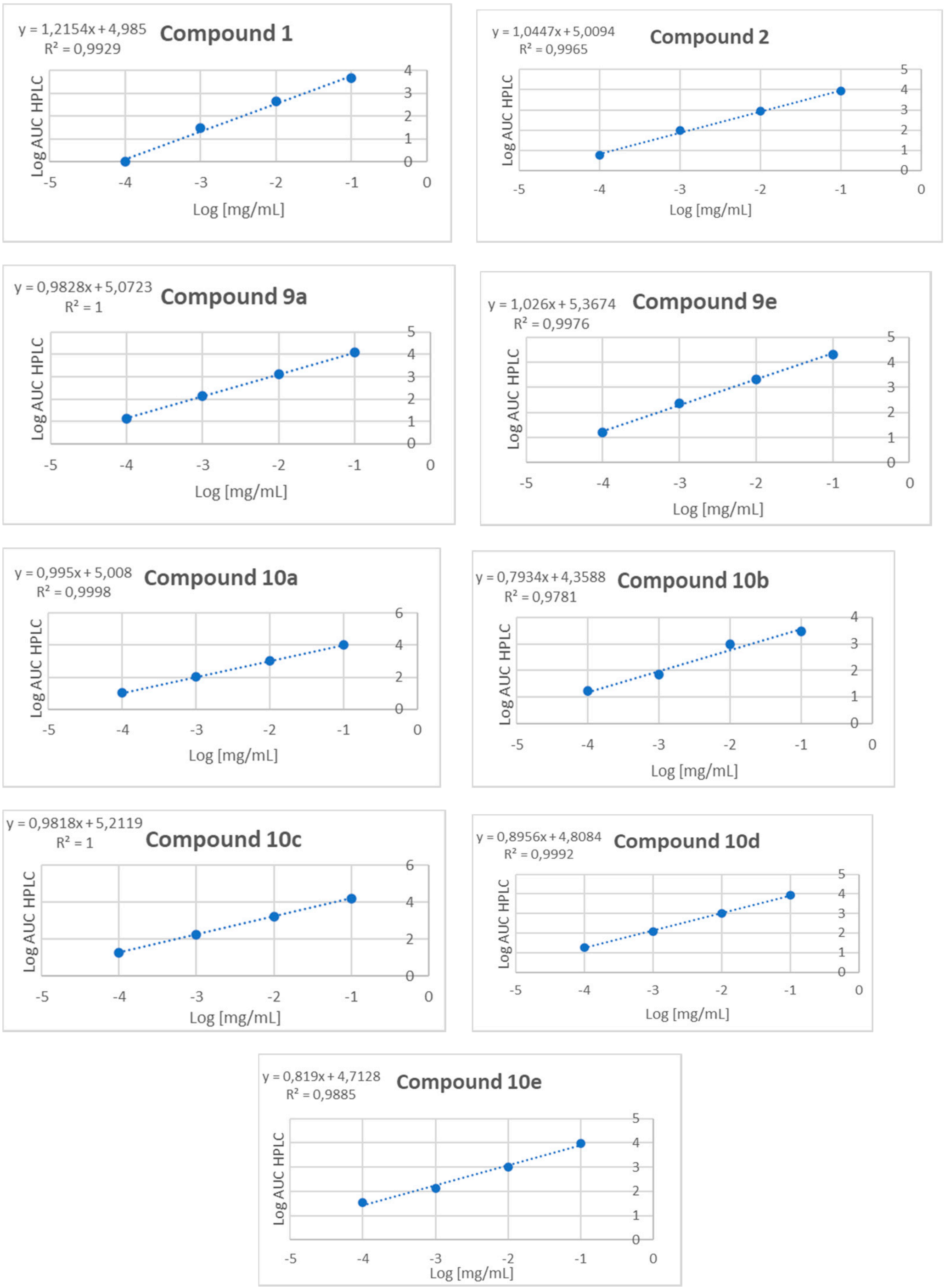

Figure S1. Calibration curves of all active compounds (solubility studies) (**1**, **2**, **9a**, **9e**, **10a-e**)

PART B. ^{1}H and ^{13}C NMR spectra.

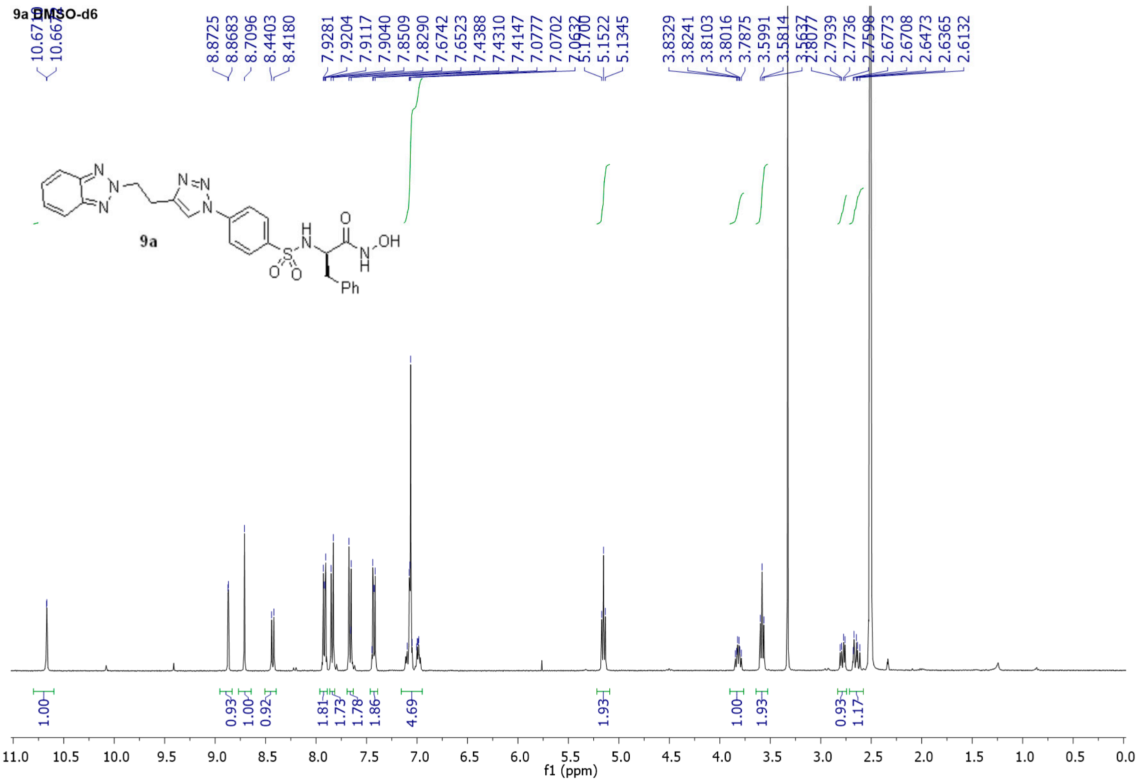

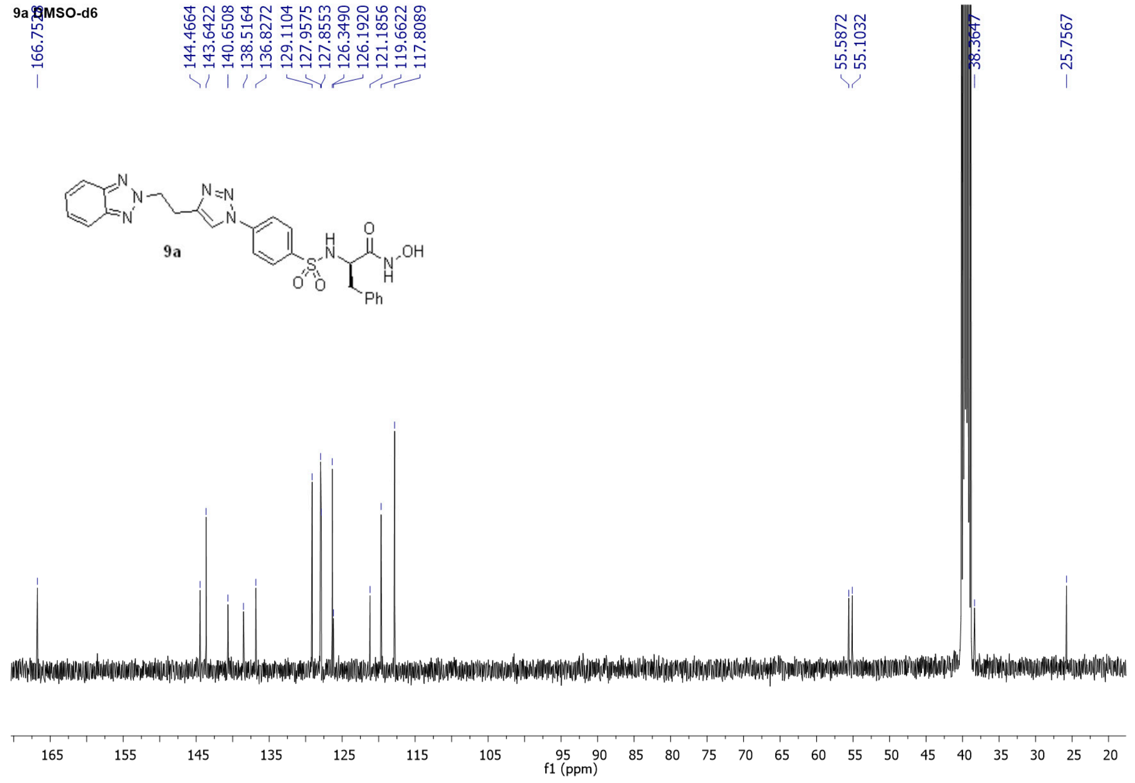

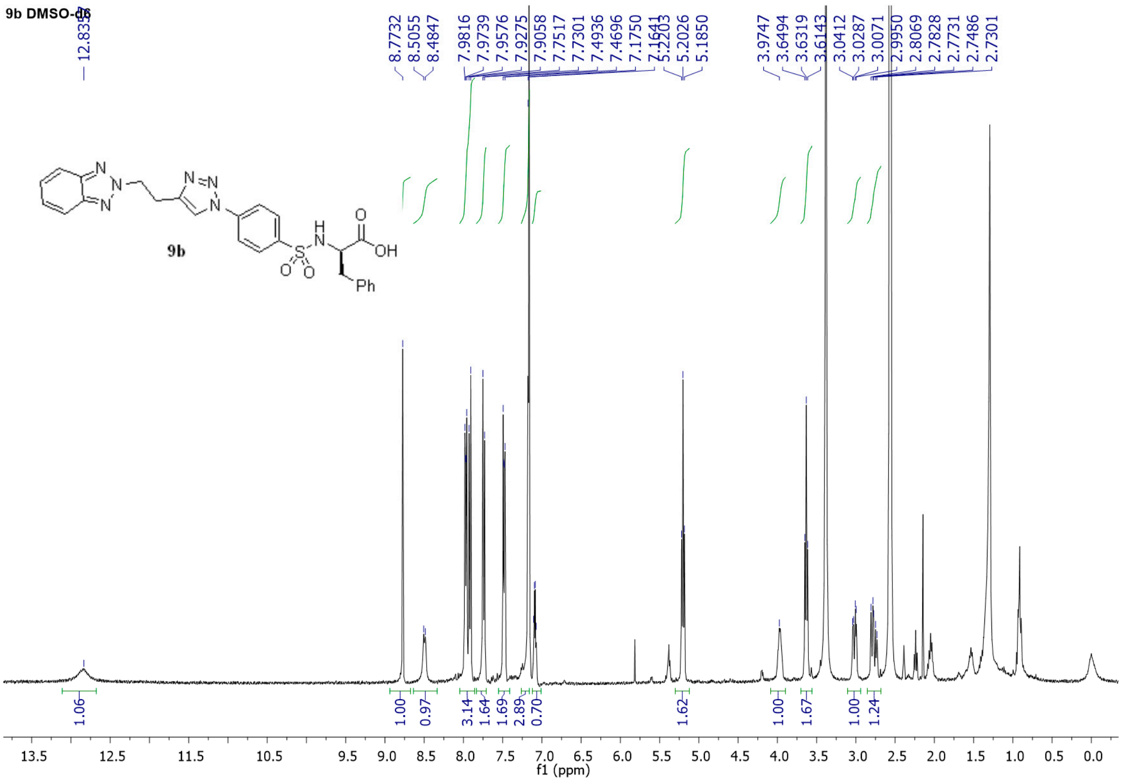
9b DMSO-d6
12.8359
8.7732
8.5055
8.4847
7.9816
7.9739
7.9576
7.9275
7.9058
7.7517
7.7301
7.4936
7.4696
7.1750
7.1641
5.2203
5.2026
5.1850
3.9747
3.6494
3.6319
3.6143
3.0412
3.0287
3.0071
2.9950
2.8069
2.7828
2.7731
2.7486
2.7301
9b
Ph
OH
1.06
1.00
0.97
3.14
1.64
1.69
2.89
0.70
1.62
1.00
1.67
1.00
1.24
13.5
12.5
11.5
10.5
9.5
9.0
8.5
8.0
7.5
7.0
6.5
6.0
5.5
5.0
4.5
4.0
3.5
3.0
2.5
2.0
1.5
1.0
0.5
0.0
f1 (ppm)

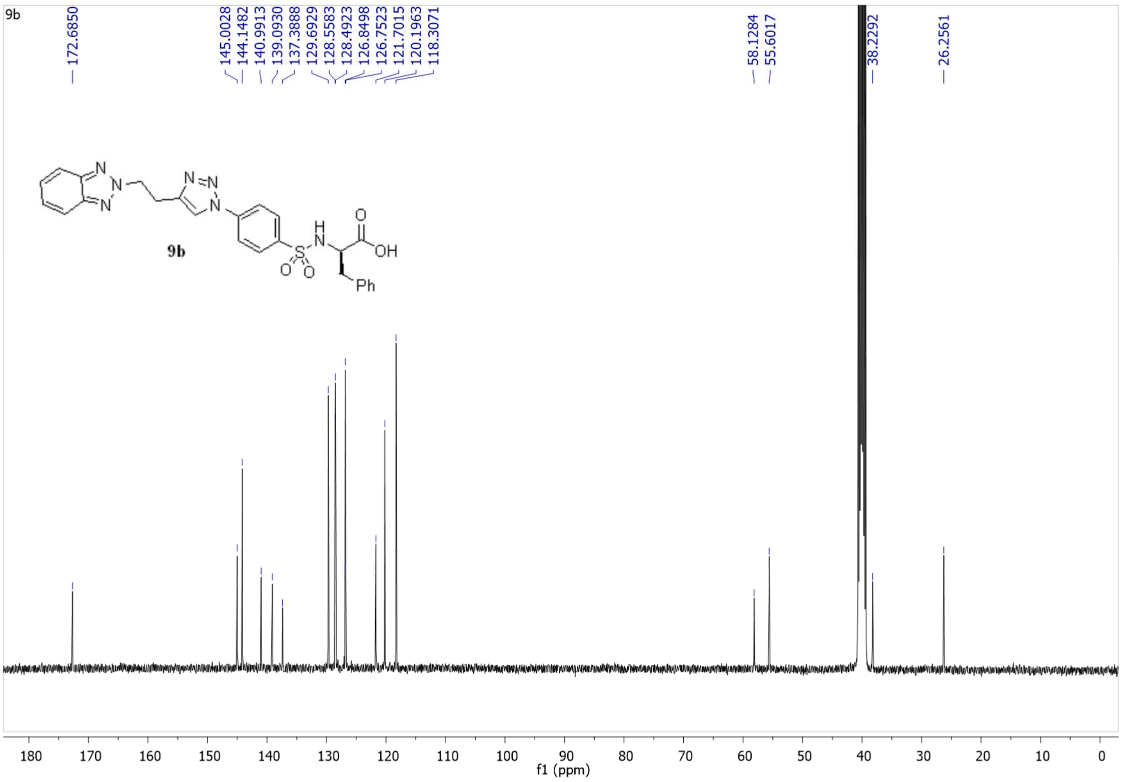
9b
172.6850
145.0028
144.1482
140.9913
139.0930
137.3888
129.6929
128.5583
128.4923
126.8498
126.7523
121.7015
120.1963
118.3071
58.1284
55.6017
38.2292
26.2561
Ph
OH
180
170
160
150
140
130
120
110
100
90
80
70
60
50
40
30
20
10
0
f1 (ppm)

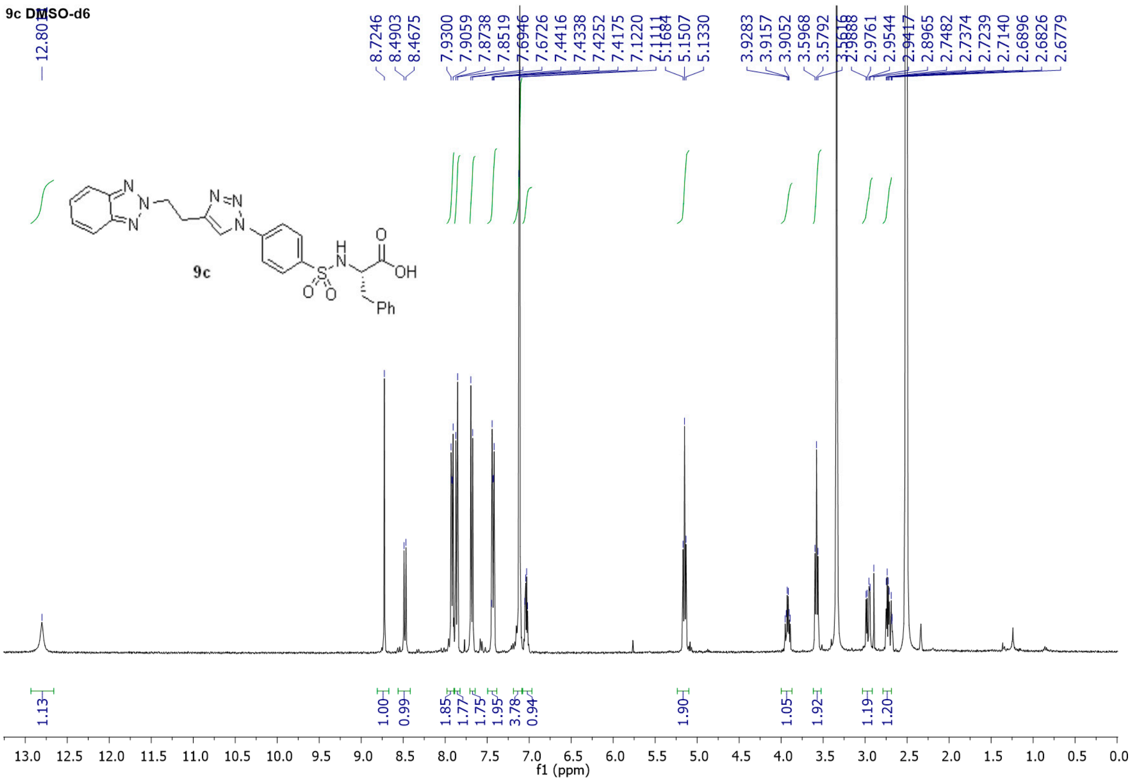
9c DMSO-d6
8.7246
8.4903
8.4675
7.9300
7.9059
7.8738
7.8519
7.6946
7.6726
7.4416
7.4338
7.4252
7.4175
7.1220
7.1111
5.1684
5.1507
5.1330
3.9283
3.9157
3.9052
3.5968
3.5792
2.9761
2.9544
2.9417
2.8965
2.7482
2.7374
2.7239
2.7140
2.6896
2.6826
2.6779
9c
Ph
OH
1.13
1.00
0.99
1.85
1.77
1.75
1.95
3.78
0.94
1.90
1.05
1.92
1.19
1.20
f1 (ppm)

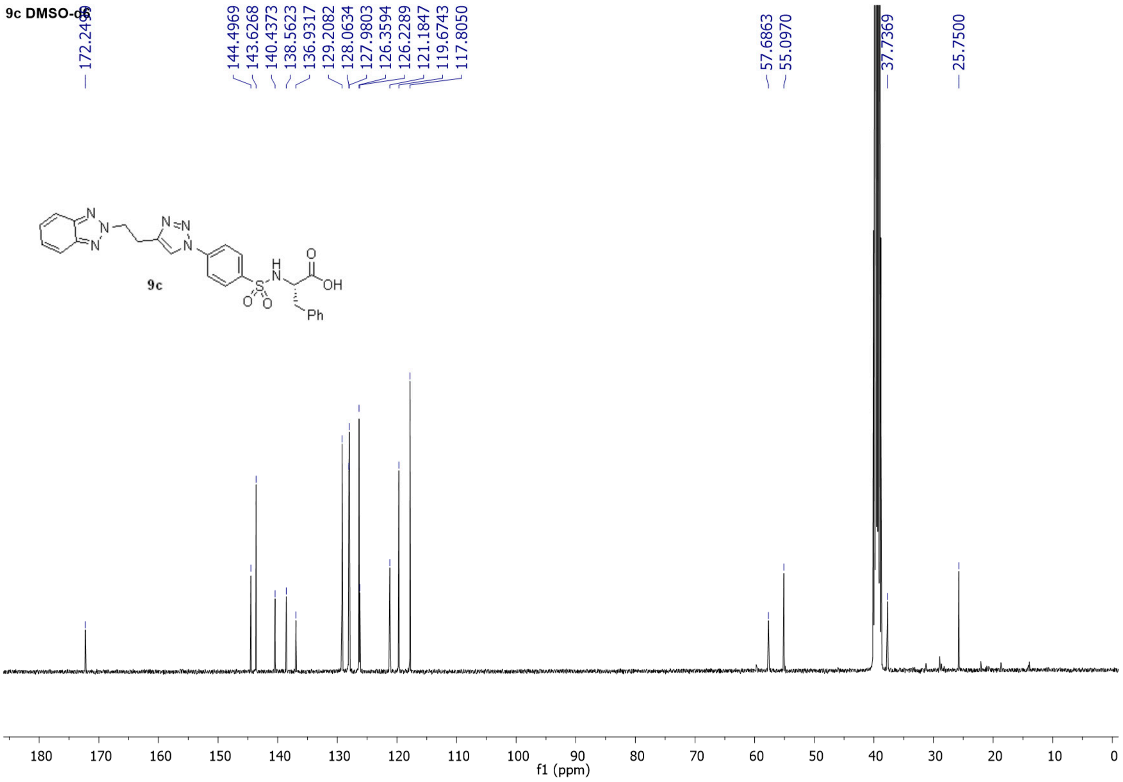
9c DMSO-d6
144.4969
143.6268
140.4373
138.5623
136.9317
129.2082
128.0634
127.9803
126.3594
126.2289
121.1847
119.6743
117.8050
57.6863
55.0970
37.7369
25.7500
9c
Ph
OH
f1 (ppm)

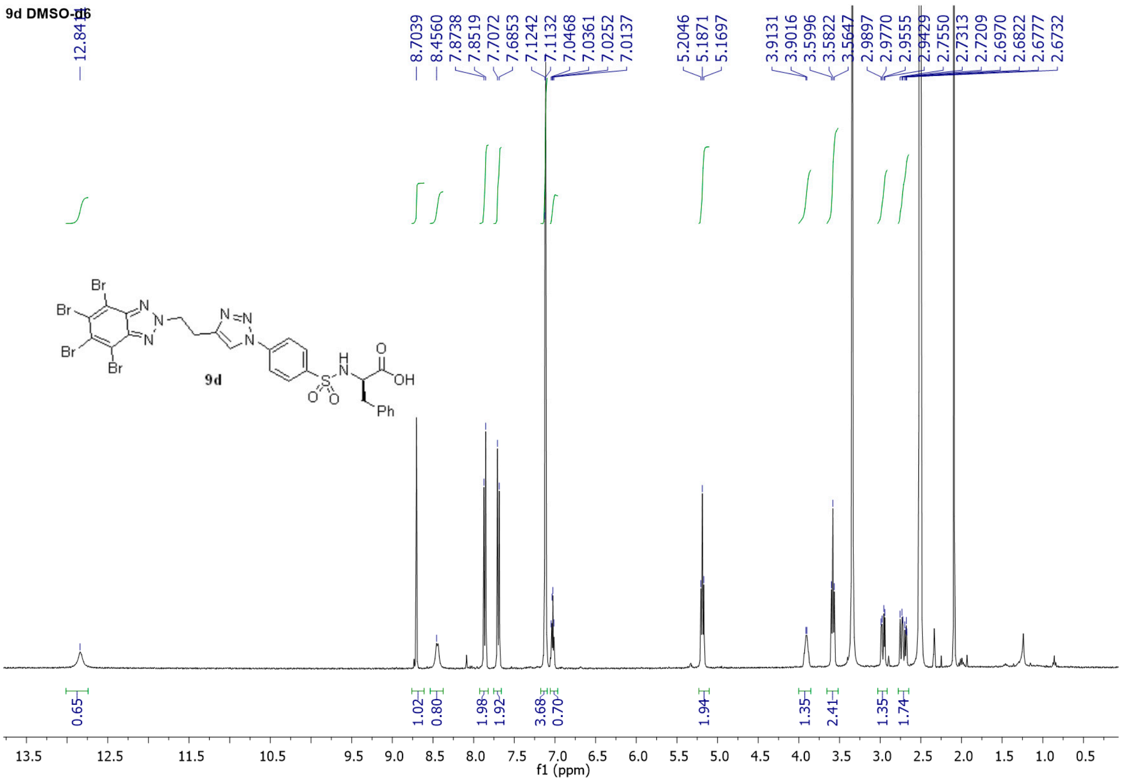
9d DMSO-d6
8.7039
8.4560
7.8738
7.8519
7.7072
7.6853
7.1242
7.1132
7.0468
7.0361
7.0252
7.0137
5.2046
5.1871
5.1697
3.9131
3.9016
3.5996
3.5822
2.9897
2.9770
2.9555
2.9429
2.7550
2.7313
2.7209
2.6970
2.6822
2.6777
2.6732
Br
Br
Br
Br
N
N
N
N
N
N
N
H
S
O
O
O
OH
Ph
9d
0.65
1.02
0.80
1.98
1.92
3.68
0.70
1.94
1.35
2.41
1.35
1.74
13.5
12.5
11.5
10.5
9.5
9.0
8.5
8.0
7.5
7.0
6.5
6.0
5.5
5.0
4.5
4.0
3.5
3.0
2.5
2.0
1.5
1.0
0.5
f1 (ppm)

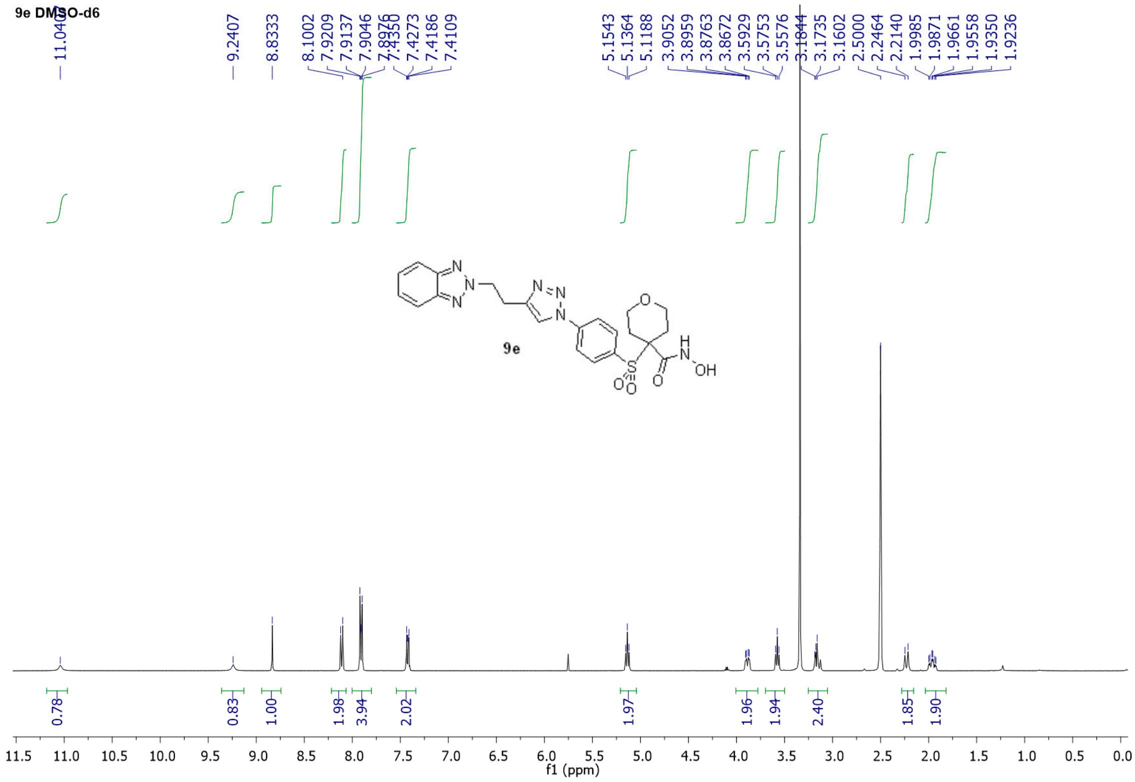
9e DMSO-d6
9.2407
8.8333
8.1002
7.9209
7.9137
7.9046
7.4273
7.4186
7.4109
5.1543
5.1364
5.1188
3.9052
3.8959
3.8763
3.8672
3.5929
3.5753
3.5576
3.1735
3.1602
2.5000
2.2464
2.2140
1.9985
1.9871
1.9661
1.9558
1.9350
1.9236
N
N
N
N
N
N
O
S
O
O
O
H
N
OH
9e
0.78
0.83
1.00
1.98
3.94
2.02
1.97
1.96
1.94
2.40
1.85
1.90
11.5
11.0
10.5
10.0
9.5
9.0
8.5
8.0
7.5
7.0
6.5
6.0
5.5
5.0
4.5
4.0
3.5
3.0
2.5
2.0
1.5
1.0
0.5
0.0
f1 (ppm)

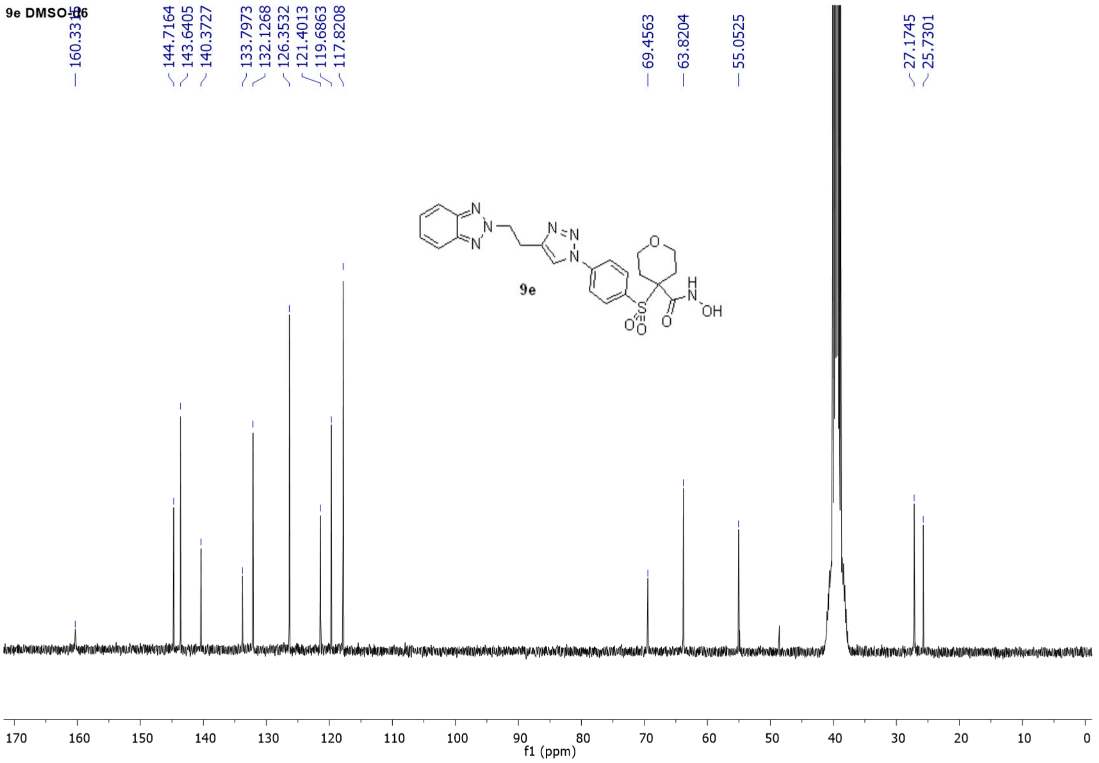
9e DMSO-d6
160.3310
144.7164
143.6405
140.3727
133.7973
132.1268
126.3532
121.4013
119.6863
117.8208
69.4563
63.8204
55.0525
27.1745
25.7301
9e
f1 (ppm)

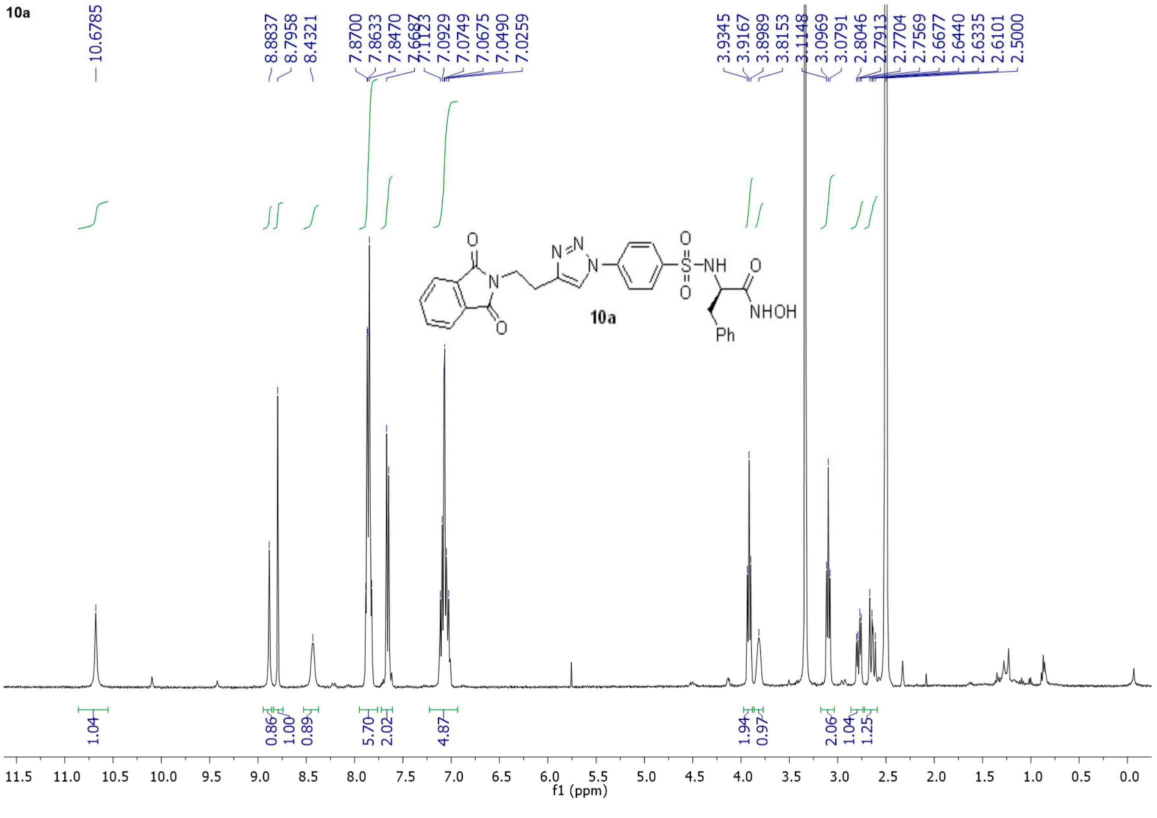
10a
10.6785
8.8837
8.7958
8.4321
7.8700
7.8633
7.8470
7.6687
7.1123
7.0929
7.0749
7.0675
7.0490
7.0259
3.9345
3.9167
3.8989
3.8153
3.1148
3.0969
3.0791
2.8046
2.7913
2.7704
2.7569
2.6677
2.6440
2.6335
2.6101
2.5000
10a
NHOH
Ph
1.04
0.86
1.00
0.89
5.70
2.02
4.87
1.94
0.97
2.06
1.04
1.25
f1 (ppm)

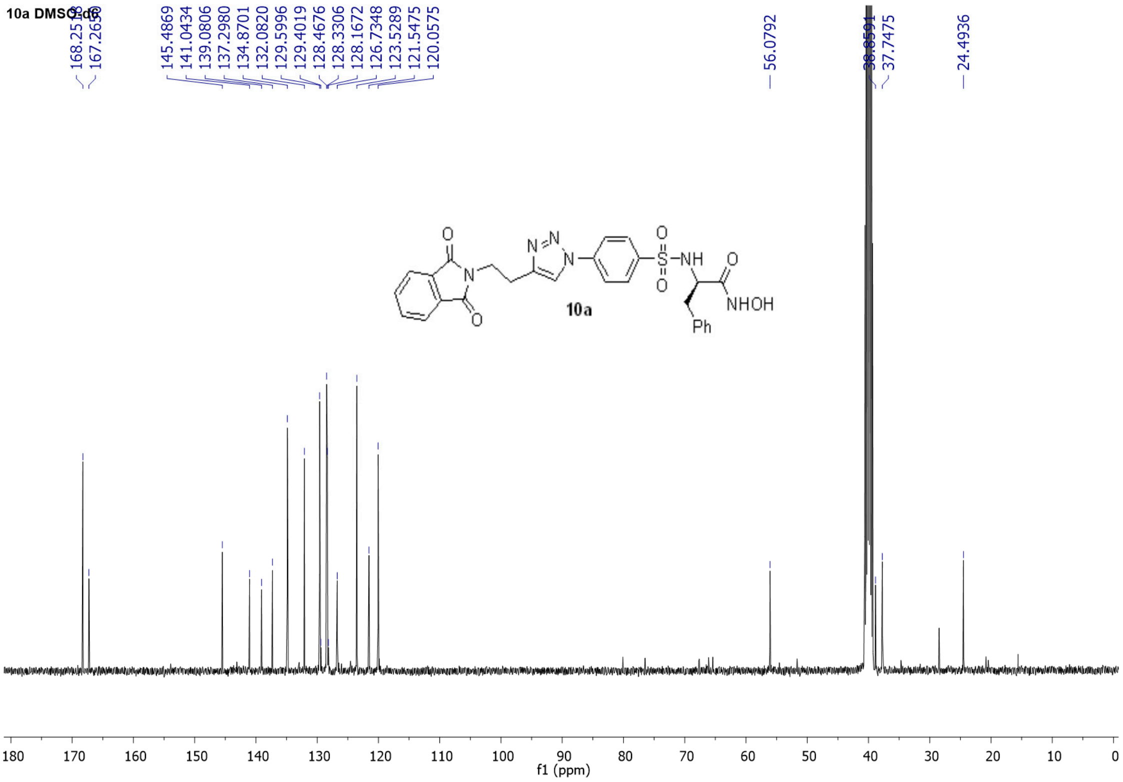
10a DMSO-d6
168.2518
167.2650
145.4869
141.0434
139.0806
137.2980
134.8701
132.0820
129.5996
129.4019
128.4676
128.3306
128.1672
126.7348
123.5289
121.5475
120.0575
56.0792
38.8591
37.7475
24.4936
10a
NHOH
Ph
f1 (ppm)

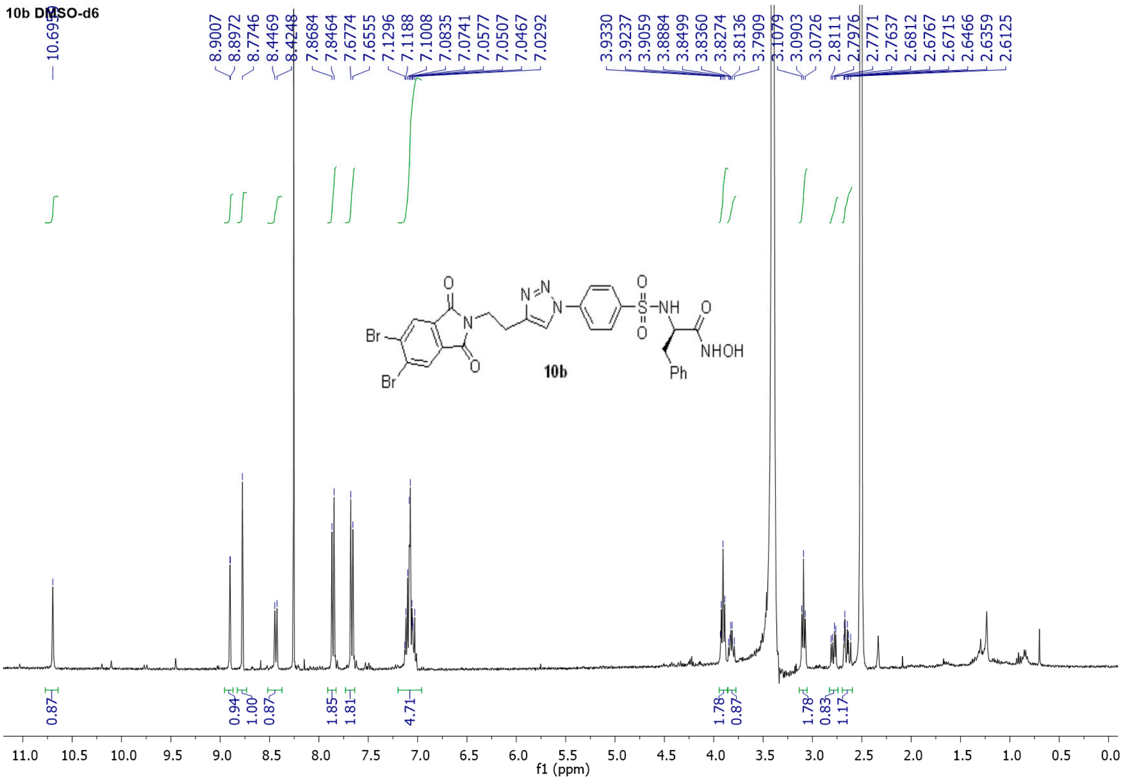
10b DMSO-d6
10.6958
8.9007
8.8972
8.7746
8.4469
8.4248
7.8684
7.8464
7.6774
7.6555
7.1296
7.1188
7.1008
7.0835
7.0741
7.0577
7.0507
7.0467
7.0292
3.9330
3.9237
3.9059
3.8884
3.8499
3.8360
3.8274
3.8136
3.7909
3.1079
3.0903
3.0726
2.8111
2.7976
2.7771
2.7637
2.6812
2.6767
2.6715
2.6466
2.6359
2.6125
10b
Br
Br
NHOH
Ph
0.87
0.94
1.00
0.87
1.85
1.81
4.71
1.78
0.87
1.78
0.83
1.17
f1 (ppm)

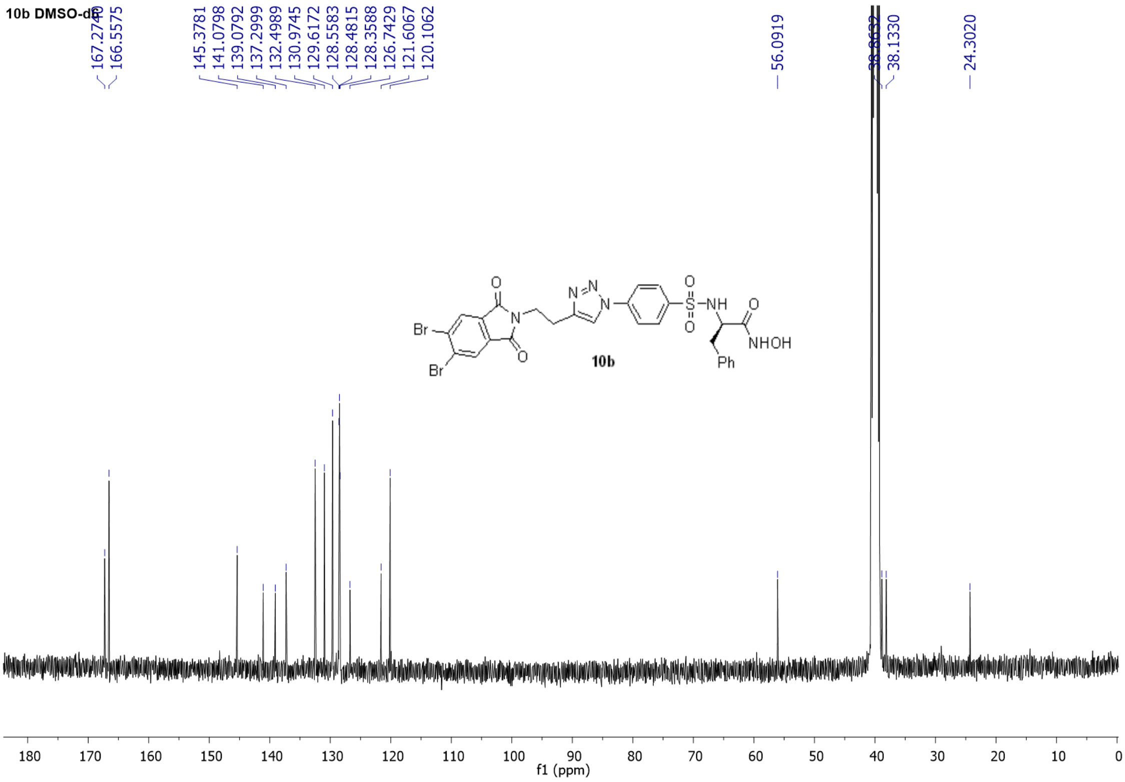
10b DMSO-d6
166.5575
145.3781
141.0798
139.0792
137.2999
132.4989
130.9745
129.6172
128.5583
128.4815
128.3588
126.7429
121.6067
120.1062
56.0919
38.1330
24.3020
Br
Br
NHOH
Ph
10b
f1 (ppm)

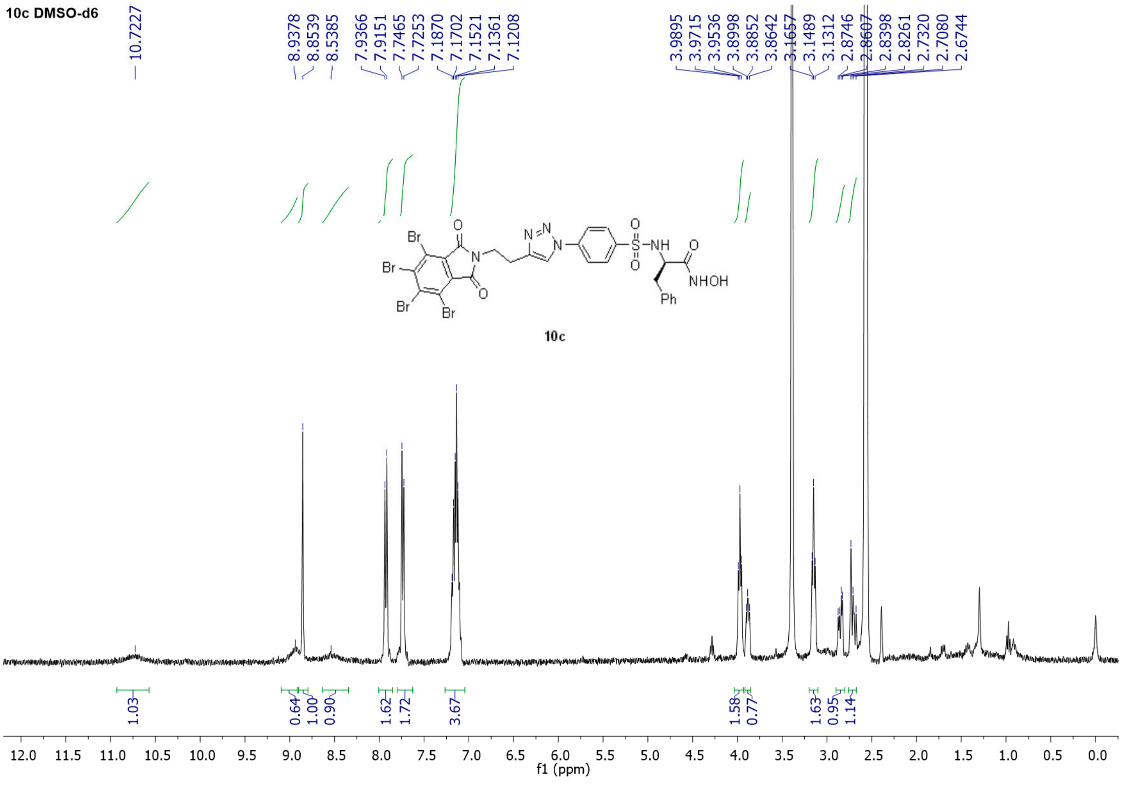
10c DMSO-d6
10.7227
8.9378
8.8539
8.5385
7.9366
7.9151
7.7465
7.7253
7.1870
7.1702
7.1521
7.1361
7.1208
3.9895
3.9715
3.9536
3.8998
3.8852
3.8642
3.1489
3.1312
2.8746
2.8398
2.8261
2.7320
2.7080
2.6744
Br
Br
Br
Br
NHOH
Ph
10c
1.03
0.64
1.00
0.90
1.62
1.72
3.67
1.58
0.77
1.63
0.95
1.14
f1 (ppm)

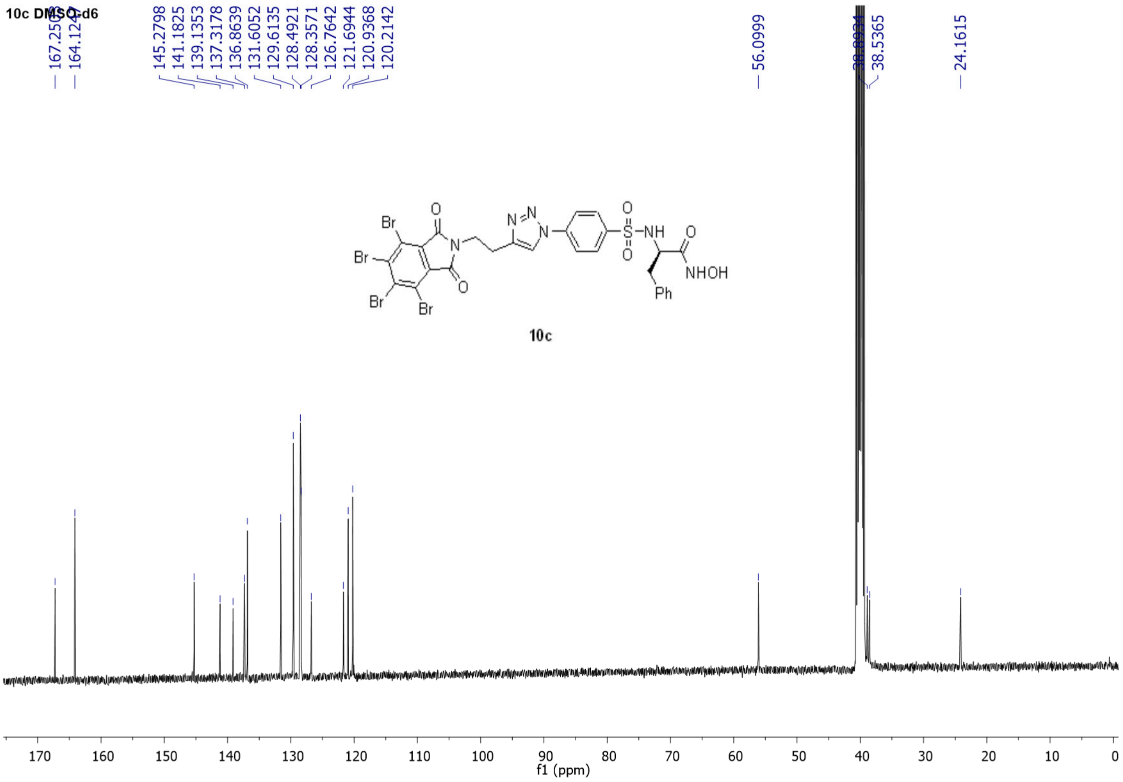
10c DMSO-d6
167.250
164.124
145.2798
141.1825
139.1353
137.3178
136.8639
131.6052
129.6135
128.4921
128.3571
126.7642
121.6944
120.9368
120.2142
56.0999
38.5365
24.1615
Br
O
N
N
N
O
S
NH
NHOH
Ph
10c
f1 (ppm)

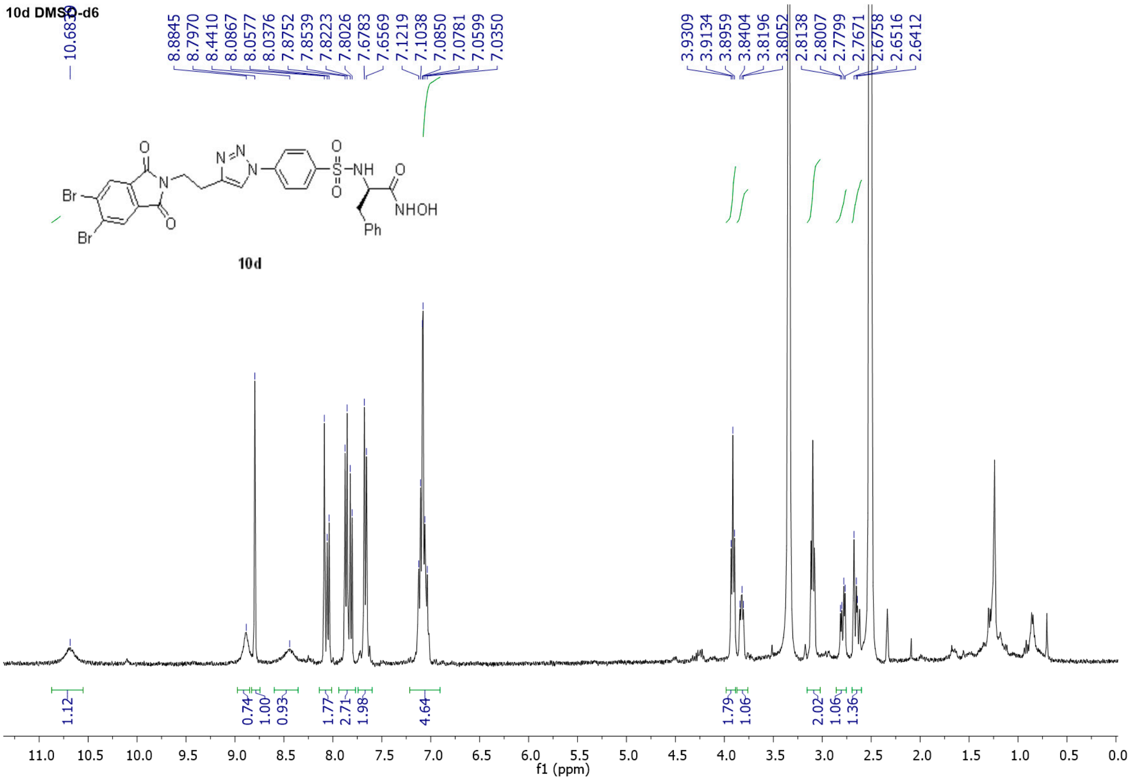
10d DMSO-d6
8.8845
8.7970
8.4410
8.0867
8.0577
8.0376
7.8752
7.8539
7.8223
7.8026
7.6783
7.6569
7.1219
7.1038
7.0850
7.0781
7.0599
7.0350
3.9309
3.9134
3.8959
3.8404
3.8196
3.8052
2.8138
2.8007
2.7799
2.7671
2.6758
2.6516
2.6412
Br
O
N
NH
NHOH
Ph
10d
1.12
0.74
1.00
0.93
1.77
2.71
1.98
4.64
1.79
1.06
2.02
1.06
1.36
f1 (ppm)

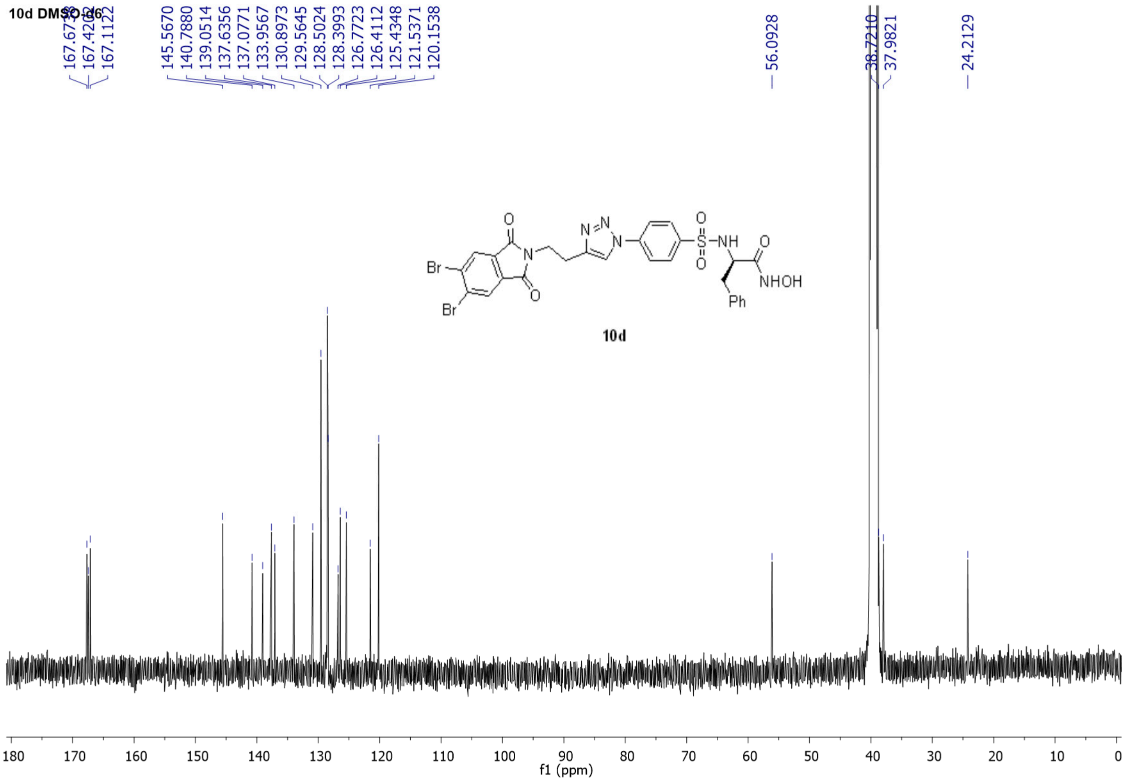
10d DMSO-d6
167.6730
167.4260
167.1122
145.5670
140.7880
139.0514
137.6356
137.0771
133.9567
130.8973
129.5645
128.5024
128.3993
126.7723
126.4112
125.4348
121.5371
120.1538
56.0928
38.7210
37.9821
24.2129
Br
Br
NH
NHOH
Ph
10d
f1 (ppm)

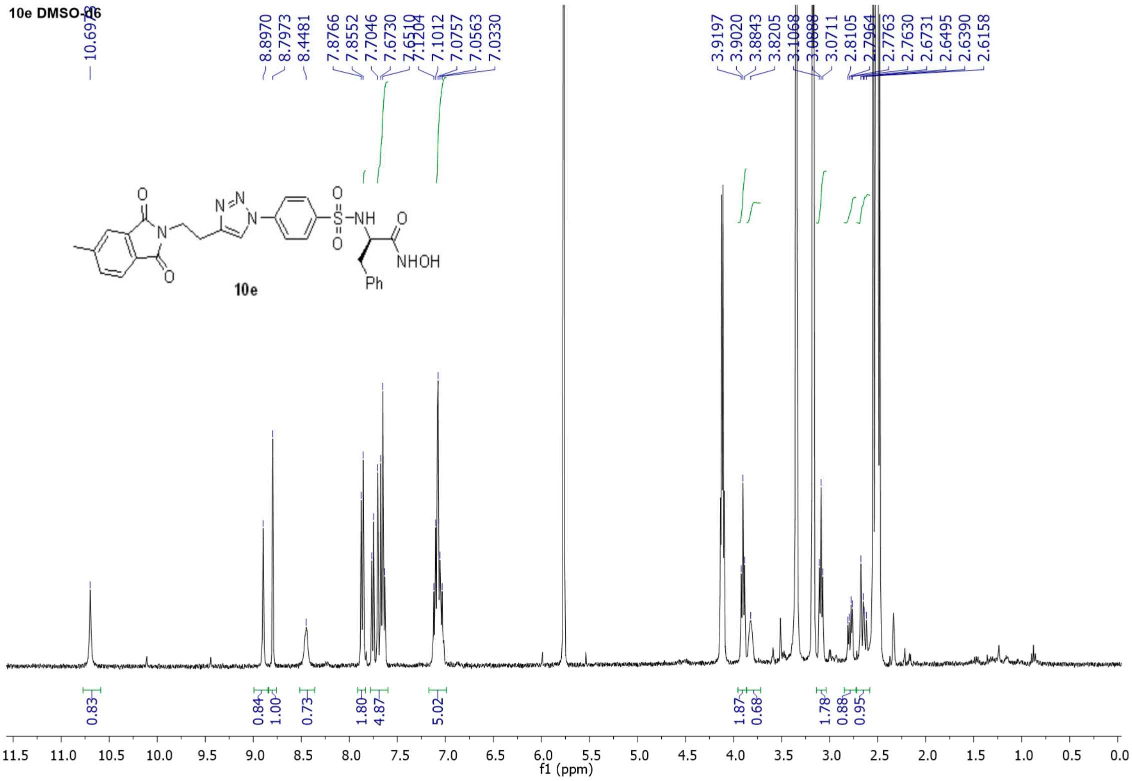
10e DMSO-d6
10.6970
8.8970
8.7973
8.4481
7.8766
7.8552
7.7046
7.6730
7.6510
7.1204
7.1012
7.0757
7.0563
7.0330
3.9197
3.9020
3.8843
3.8205
3.1068
3.0888
3.0711
2.8105
2.7964
2.7763
2.7630
2.6731
2.6495
2.6390
2.6158
NH
NHOH
Ph
10e
0.83
0.84
1.00
0.73
1.80
4.87
5.02
1.87
0.68
1.78
0.88
0.95
f1 (ppm)

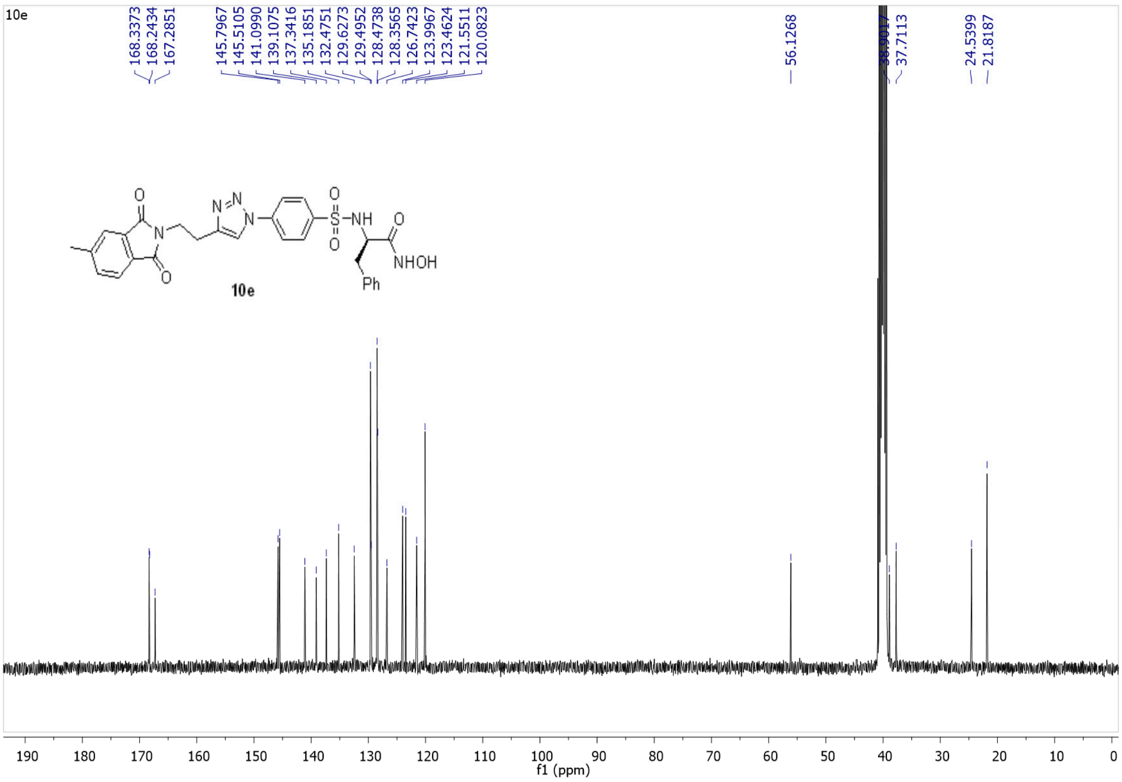

Figure S2. ^{1}H and ^{13}C NMR spectra for final compounds.

PART C. Modeling

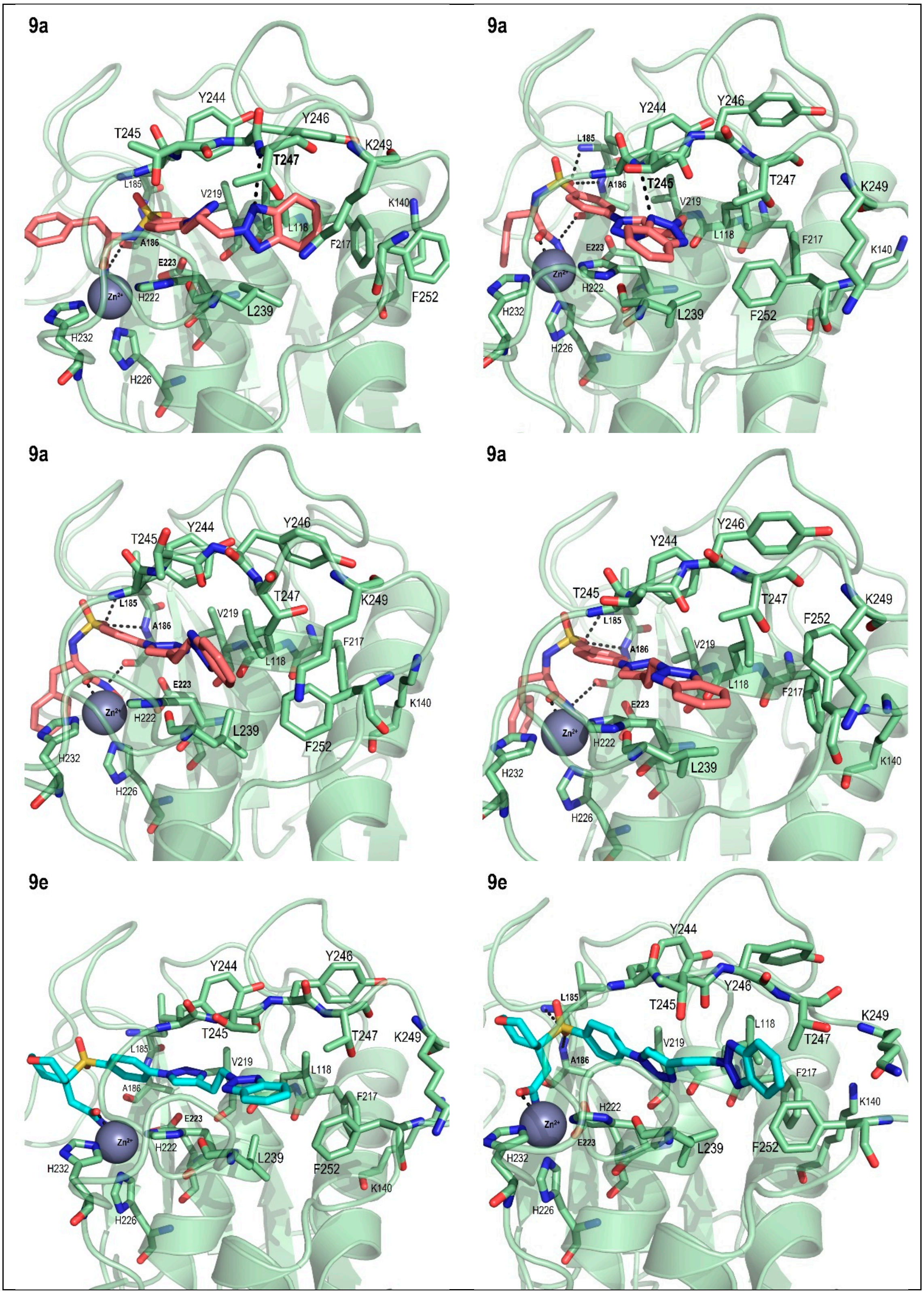

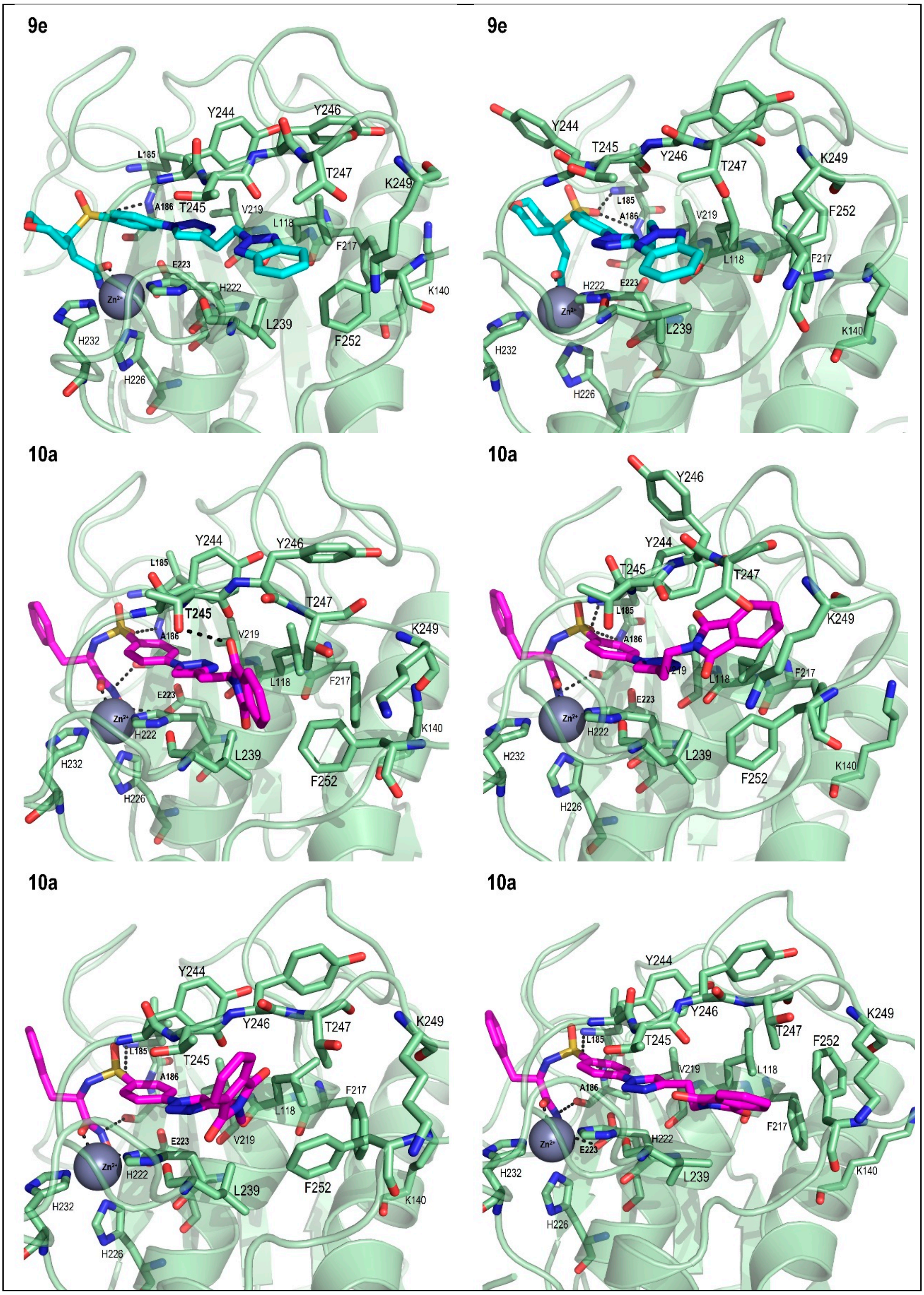

9e
9e
10a
10a
10a
10a
Y244
Y246
T245
T247
K249
L185
A186
V219
L118
F217
E223
H222
Zn2+
H232
H226
L239
F252
K140

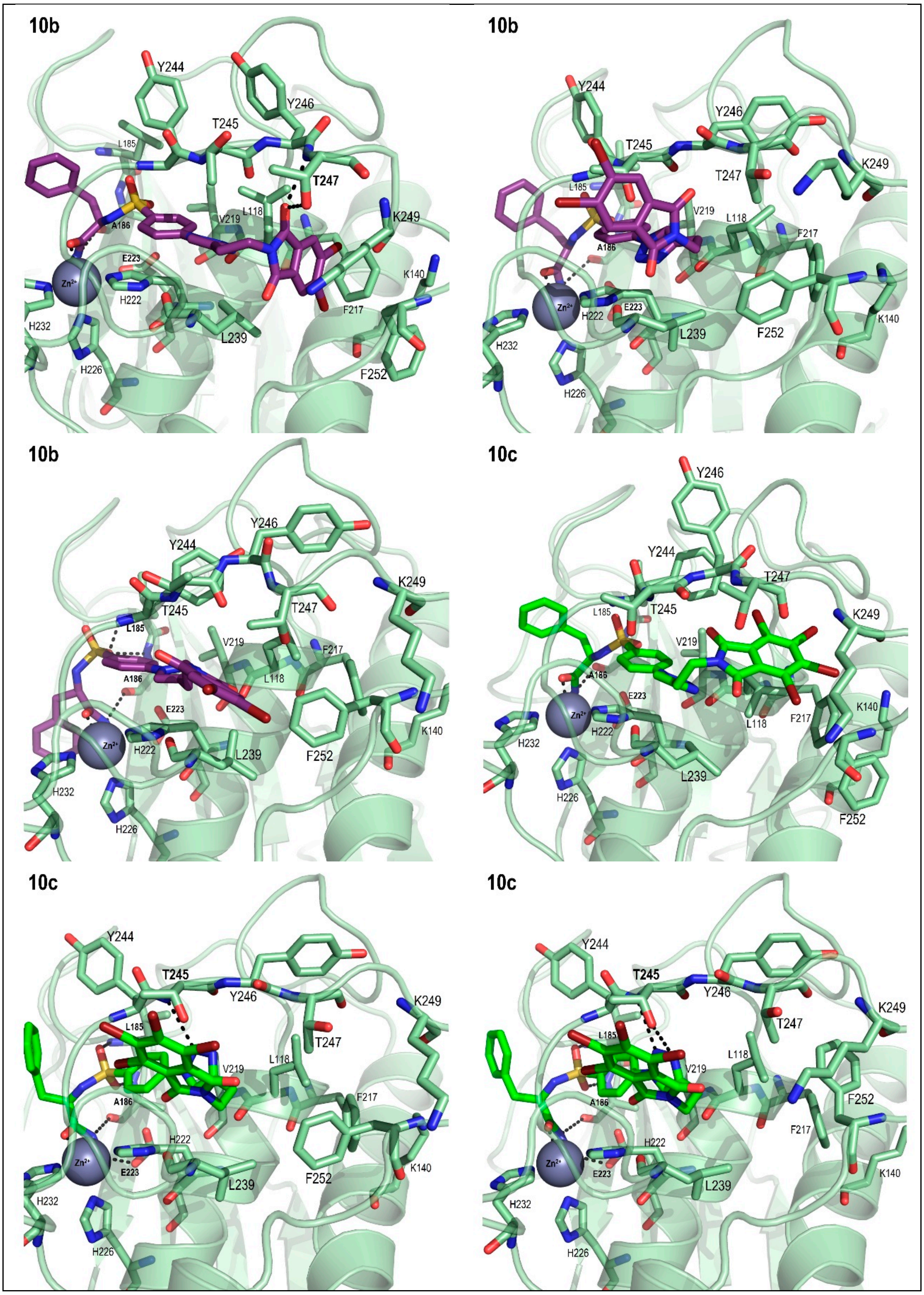
10b
Y244
Y246
T245
L185
T247
A186
L118
V219
K249
E223
Zn2+
H222
K140
H232
F217
L239
H226
F252
10b
Y244
Y246
T245
K249
T247
L185
V219
L118
A186
F217
Zn2+
H222
E223
K140
L239
F252
H232
H226
10b
Y246
Y244
K249
T245
L185
T247
V219
F217
A186
L118
E223
Zn2+
H222
L239
K140
F252
H232
H226
10c
Y246
Y244
T247
L185
T245
K249
V219
A186
E223
K140
Zn2+
H222
L118
F217
H232
L239
H226
F252
10c
Y244
T245
Y246
K249
L185
T247
L118
V219
A186
F217
H222
Zn2+
E223
L239
F252
K140
H232
H226
10c
Y244
T245
Y246
K249
T247
L185
L118
V219
A186
F252
F217
H222
Zn2+
E223
L239
K140
H232
H226

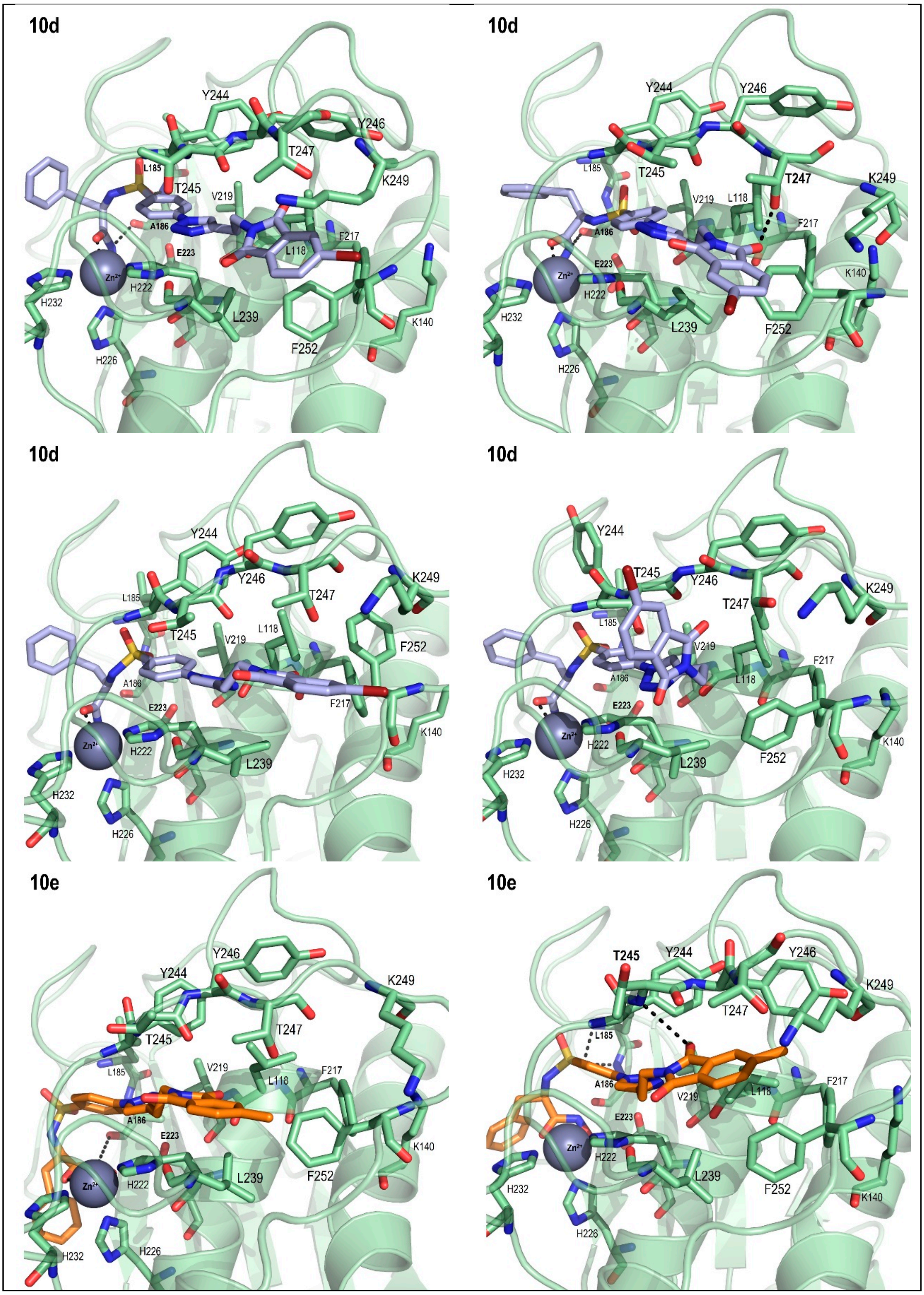
10d
Y244
Y246
T247
L185
T245
K249
V219
A186
F217
E223
L118
Zn2+
H222
H232
L239
K140
F252
H226
10d
Y244
Y246
L185
T245
K249
T247
V219
L118
A186
F217
E223
K140
H222
H232
L239
F252
H226
10d
Y244
Y246
K249
T247
L185
T245
V219
L118
F252
A186
F217
E223
K140
H222
L239
H232
H226
10d
Y244
T245
Y246
K249
T247
L185
V219
A186
L118
F217
E223
H222
K140
L239
F252
H232
H226
10e
Y246
Y244
K249
T247
T245
V219
L185
F217
L118
A186
E223
K140
L239
F252
H222
H232
H226
10e
T245
Y244
Y246
K249
T247
L185
A186
F217
V219
L118
E223
H222
L239
F252
H232
K140
H226

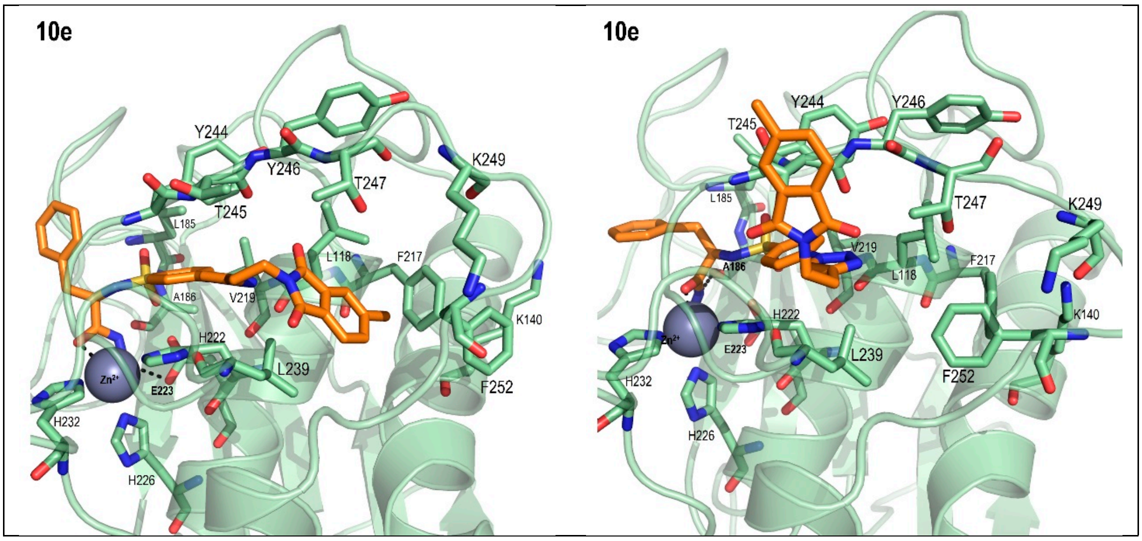

Figure S3. PyMOL stick and cartoon representation of the interactions established with the Ω-loop and S1′ obtained in the additional binding modes of the series of compounds when docked to the different crystal structures of MMP-13. Hydrogen bonds are shown in dashed lines, the catalytic zinc ion is shown as a grey sphere. For the sake of clarity, no hydrogens are shown.

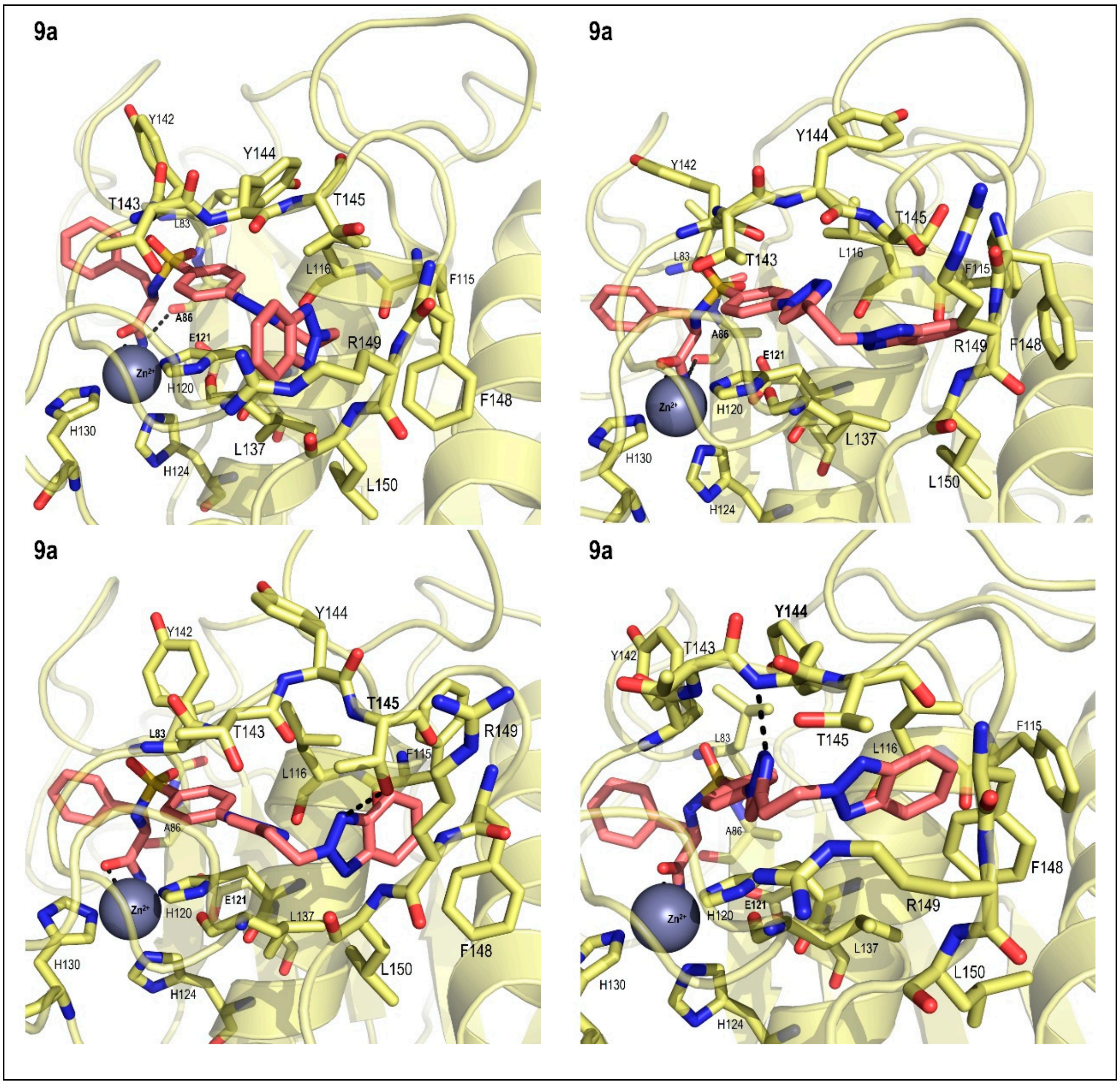

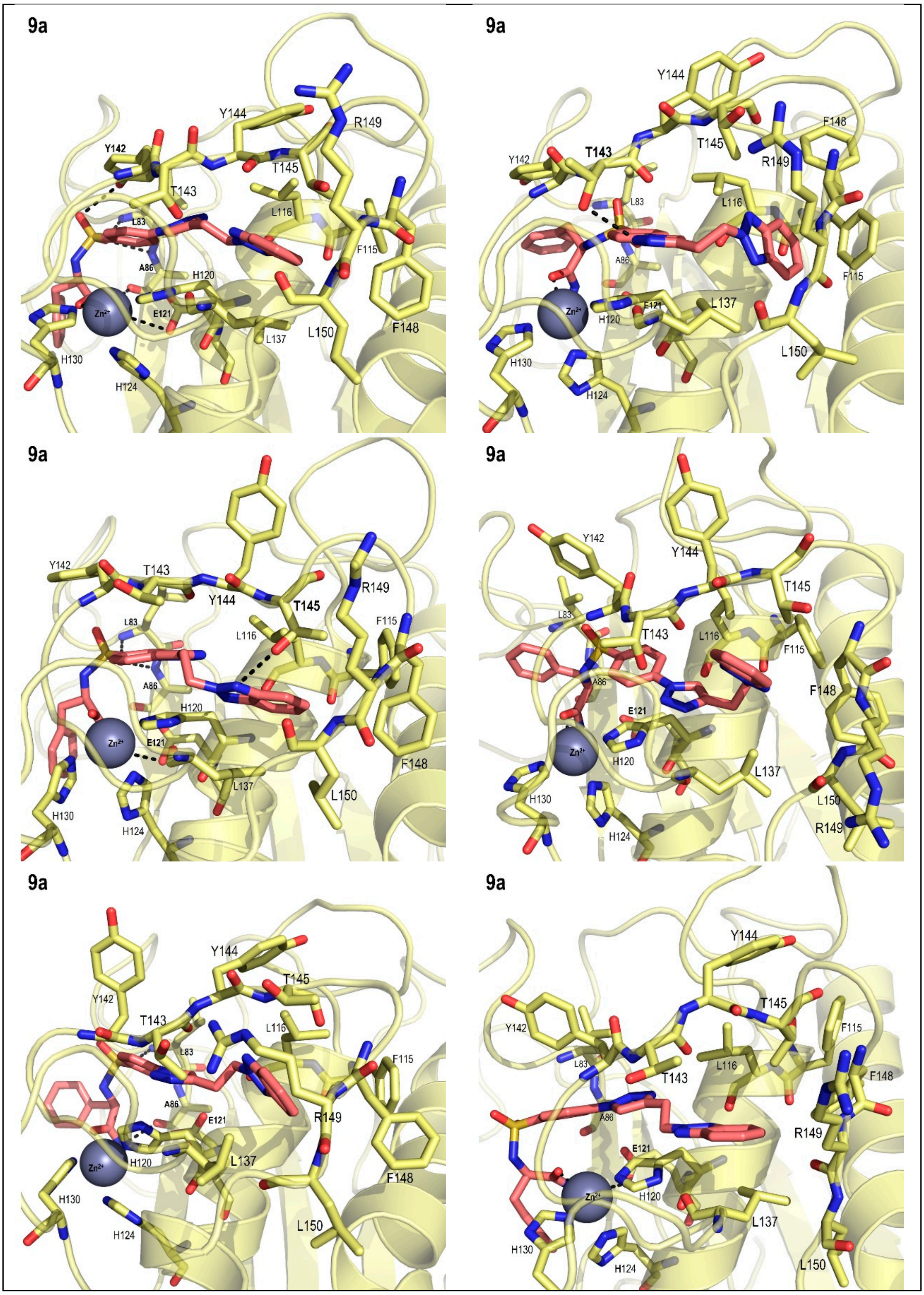
9a
Y144
R149
Y142
T145
T143
L116
L83
F115
A86
H120
Zn2+
E121
L150
F148
L137
H130
H124
9a
Y144
F148
T145
Y142
T143
R149
L83
L116
A86
F115
Zn2+
H120
E121
L137
L150
H130
H124
9a
Y142
T143
Y144
T145
R149
L83
L116
F115
A86
H120
Zn2+
E121
F148
L137
L150
H130
H124
9a
Y142
Y144
T145
L83
T143
L116
F115
A86
F148
E121
Zn2+
H120
L137
H130
L150
R149
H124
9a
Y144
T145
Y142
T143
L116
L83
F115
A86
E121
R149
H120
Zn2+
L137
F148
H130
H124
L150
9a
Y144
T145
Y142
F115
L83
T143
L116
F148
A86
R149
E121
Zn2+
H120
L137
H130
H124
L150

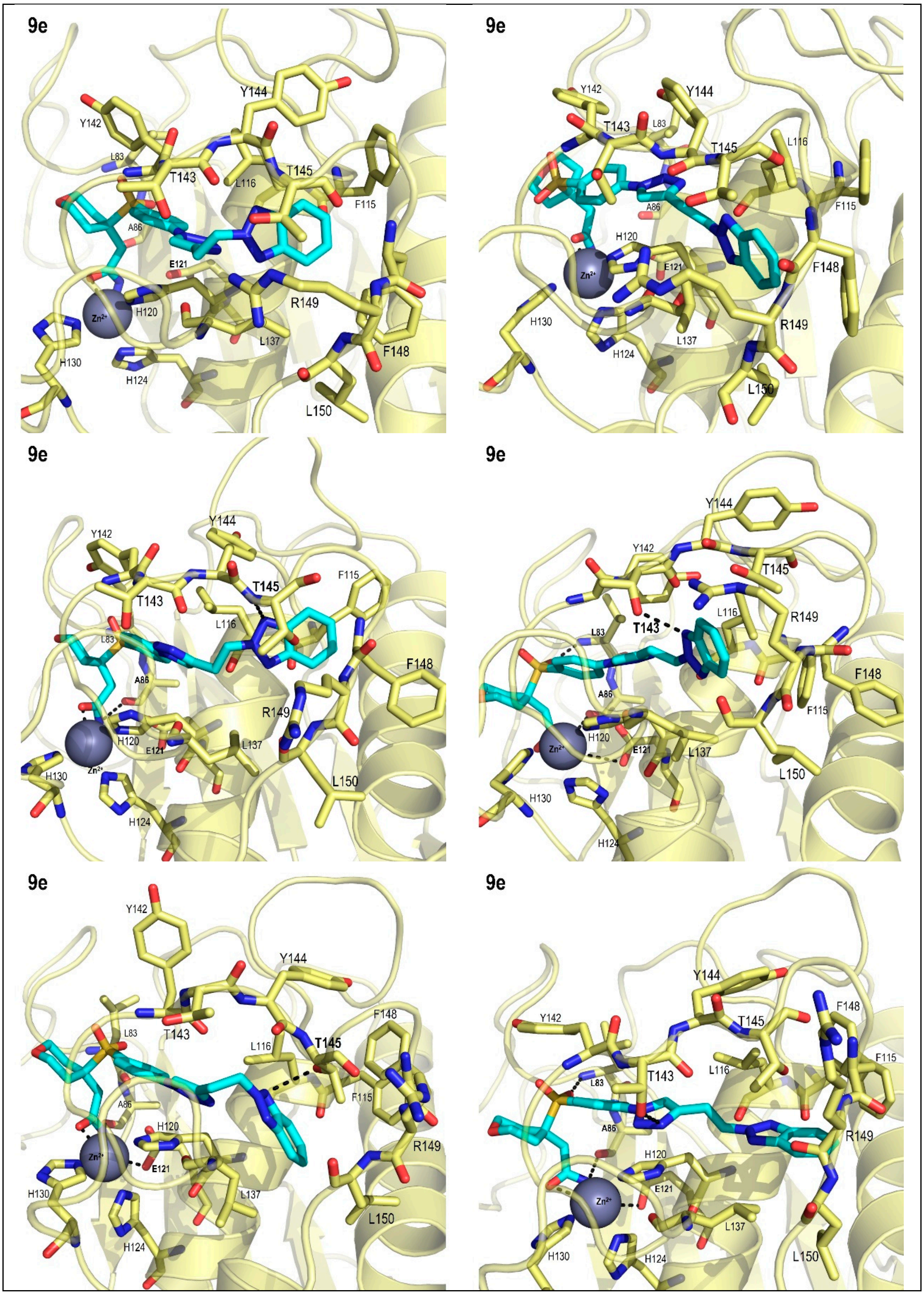
9e
Y144
Y142
L83
T143
L116
T145
F115
A86
E121
Zn2+
H120
R149
L137
H130
H124
F148
L150
9e
Y142
Y144
T143
L83
T145
L116
A86
F115
H120
E121
Zn2+
F148
H130
R149
L137
H124
L150
9e
Y144
Y142
T145
F115
T143
L116
L83
F148
A86
R149
H120
E121
L137
H130
Zn2+
L150
H124
9e
Y144
Y142
T145
L116
R149
L83
T143
F148
A86
F115
H120
Zn2+
E121
L137
L150
H130
H124
9e
Y142
Y144
L83
T143
F148
L116
T145
F115
A86
H120
R149
Zn2+
E121
H130
L137
L150
H124
9e
Y144
F148
Y142
T145
F115
L83
T143
L116
A86
R149
H120
E121
Zn2+
L137
L150
H130
H124

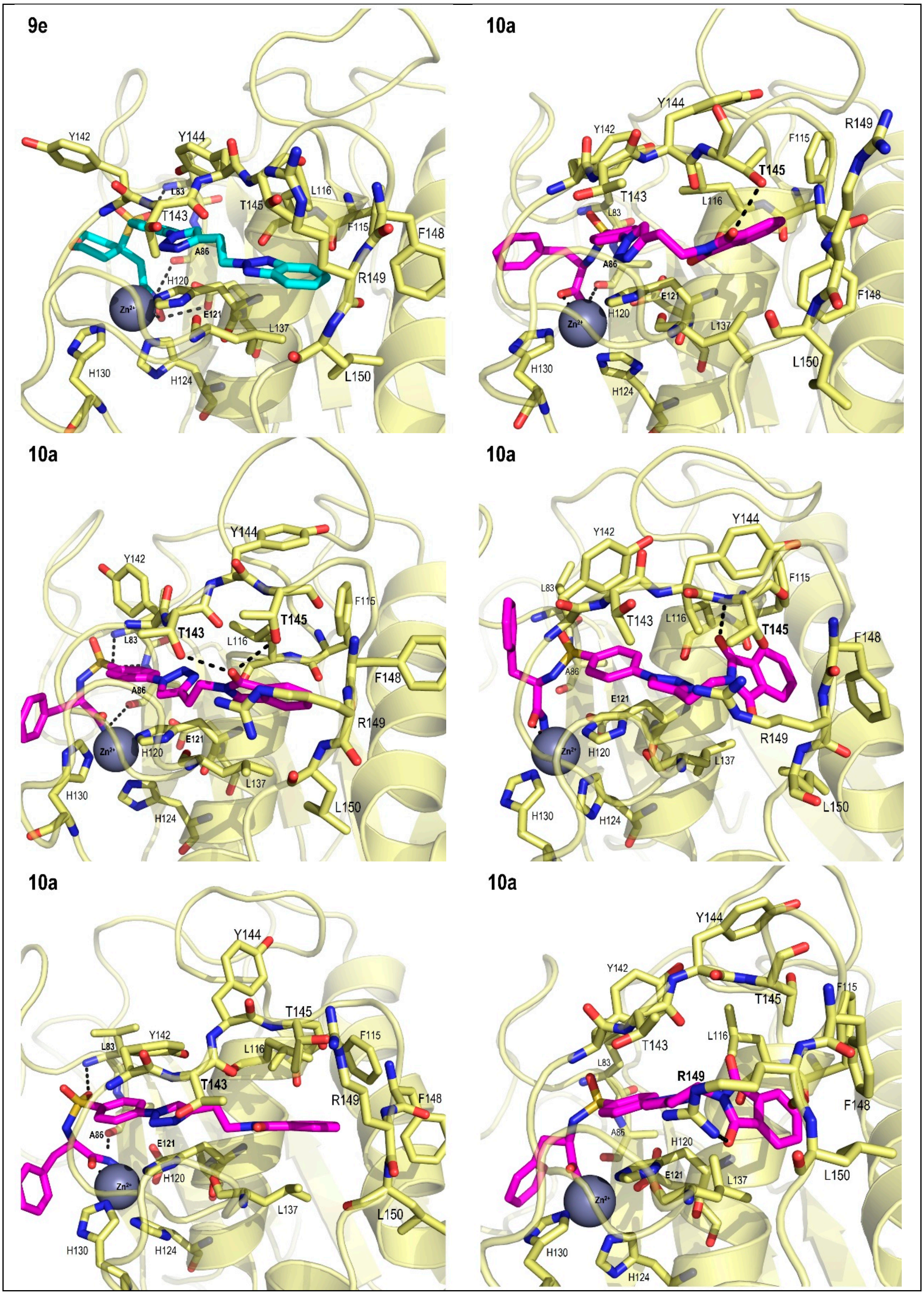
9e
Y142
Y144
L83
T143
T145
L116
F115
F148
A86
H120
R149
Zn²⁺
E121
L137
H130
H124
L150
10a
Y144
Y142
R149
F115
T145
T143
L83
L116
A86
E121
H120
L137
F148
H130
L150
H124
10a
Y144
Y142
F115
T143
T145
L83
L116
F148
A86
R149
Zn²⁺
H120
E121
L137
H130
L150
H124
10a
Y144
Y142
F115
L83
T143
L116
T145
F148
A86
E121
R149
Zn²⁺
H120
L137
H130
H124
L150
10a
Y144
T145
Y142
F115
L83
L116
T143
R149
F148
A86
E121
Zn²⁺
H120
L137
L150
H130
H124
10a
Y144
Y142
F115
T145
L116
T143
L83
R149
F148
A86
H120
L150
E121
L137
Zn²⁺
H130
H124

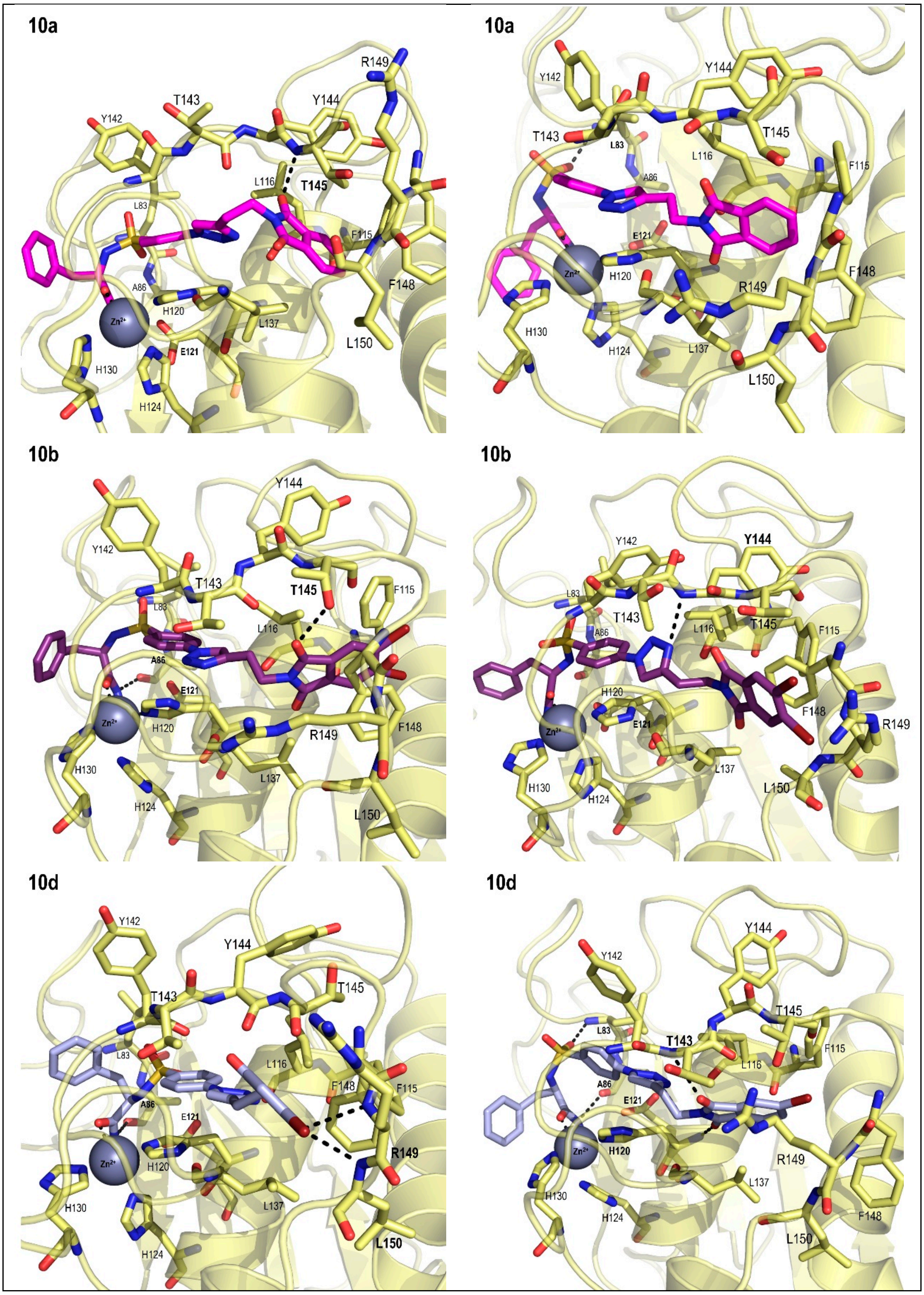
10a
R149
T143
Y144
Y142
L116
T145
L83
F115
A86
F148
H120
L137
L150
Zn2+
E121
H130
H124
10a
Y144
Y142
T143
L83
T145
L116
A86
F115
E121
Zn2+
H120
F148
R149
H130
H124
L137
L150
10b
Y144
Y142
T143
T145
F115
L83
L116
A86
E121
Zn2+
H120
R149
F148
H130
L137
H124
L150
10b
Y142
Y144
L83
T143
L116
T145
A86
F115
H120
Zn2+
E121
F148
R149
L137
H130
H124
L150
10d
Y142
Y144
T143
T145
L83
L116
F148
F115
A86
E121
R149
Zn2+
H120
H130
L137
H124
L150
10d
Y144
Y142
T145
L83
T143
F115
L116
A86
E121
Zn2+
H120
R149
L137
H130
H124
F148
L150

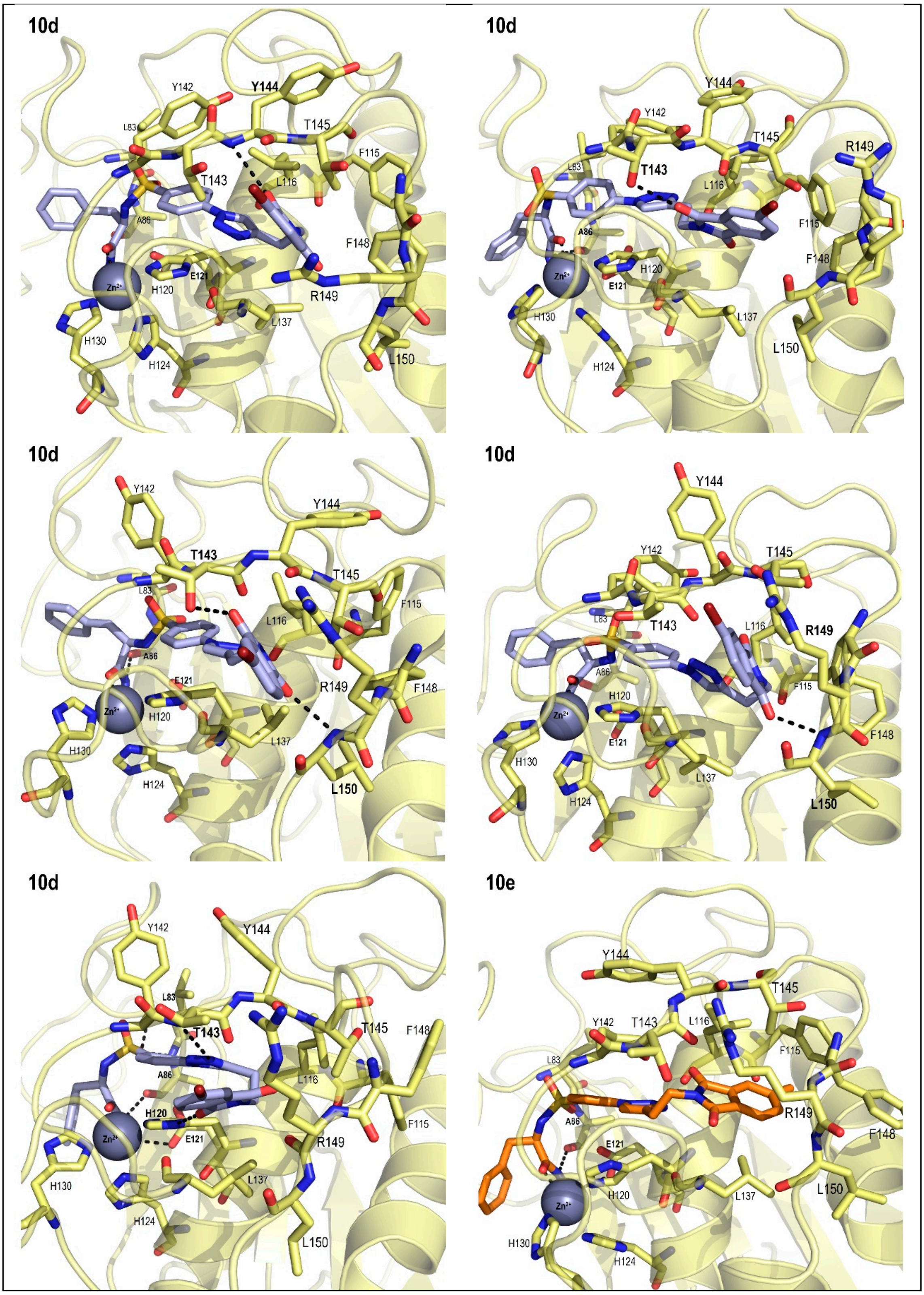
10d
Y142
Y144
L83
T145
F115
T143
L116
A86
F148
E121
Zn2+
H120
R149
L137
H130
H124
L150
10d
Y144
Y142
T145
R149
L83
T143
L116
F115
A86
F148
H120
Zn2+
E121
L137
H130
L150
H124
10d
Y142
Y144
T143
T145
L83
F115
L116
A86
E121
R149
F148
Zn2+
H120
L137
H130
L150
H124
10d
Y144
Y142
T145
L83
T143
L116
R149
A86
F115
H120
Zn2+
E121
F148
H130
L137
H124
L150
10d
Y142
Y144
L83
T143
T145
F148
A86
L116
H120
Zn2+
E121
F115
R149
L137
H130
H124
L150
10e
Y144
T145
Y142
T143
L116
F115
L83
R149
A86
E121
F148
L150
Zn2+
H120
L137
H130
H124

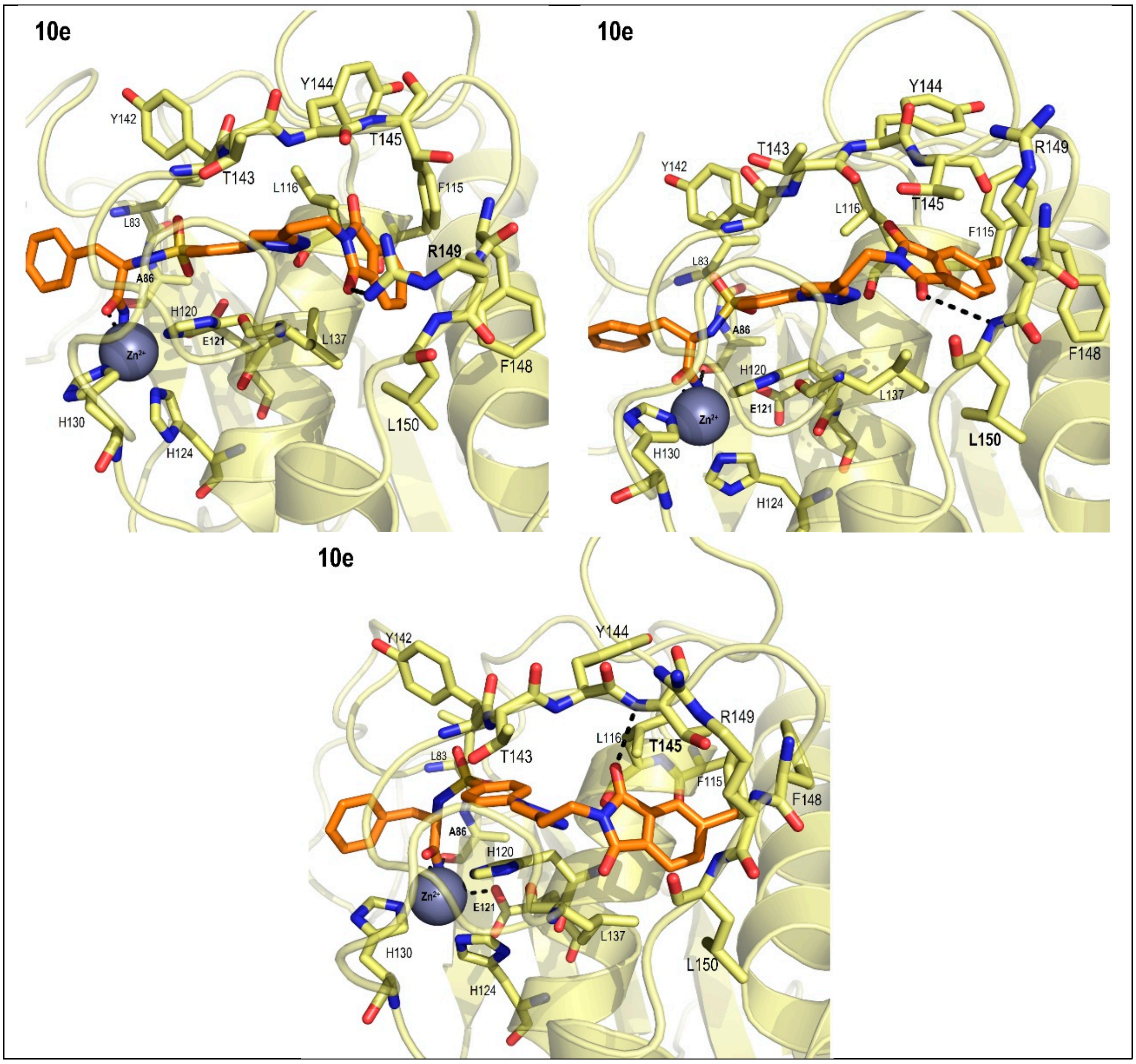

Figure S4. PyMOL stick and cartoon representation of the interactions established with the Ω-loop and S1’ pocket obtained in the additional binding modes of the series of compounds when docked to the different NMR models of MMP-2. Hydrogen bonds are shown in dashed lines, the catalytic zinc ion is shown as a grey sphere. For the sake of clarity, hydrogens are not shown.

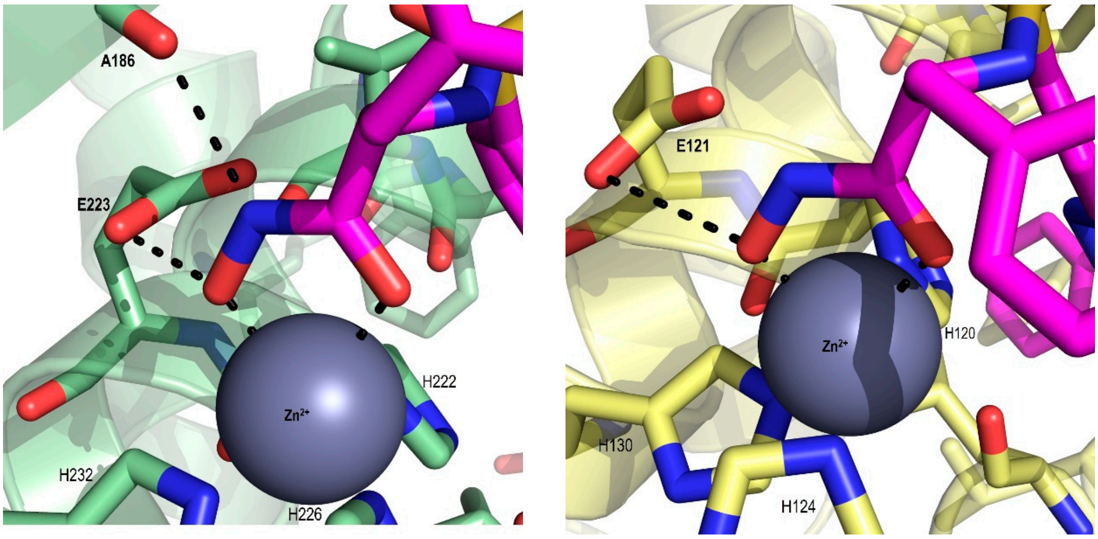

Figure S5. PyMOL stick and cartoon representation of the bidentate interaction established by the hydroxamate and the catalytic Zinc ion in both MMP-13 (left) and MMP-2 (right). Hydrogen bonds are shown in dashed lines, the catalytic zinc ion is shown as grey sphere. For the sake of clarity, no hydrogens are shown.

PART D. NMR studies

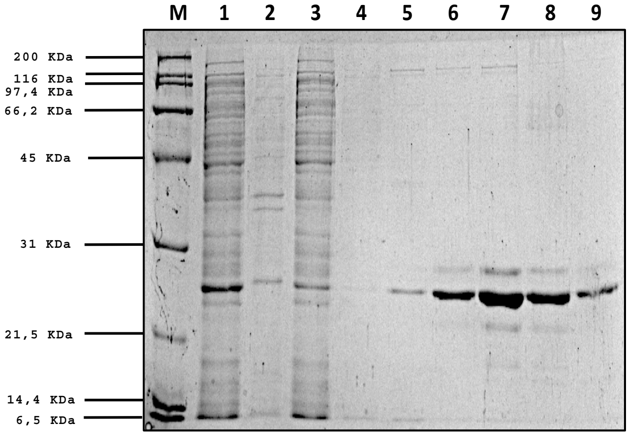

Figure S6. SDS-PAGE analysis of the recombinantly expressed catalytic domain of MMP-13 purified by metal affinity chromatography. M corresponds to the molecular weight markers (kDa). Lane 1: supernatant of total bacterial protein; lane 2: precipitate of total bacterial protein; lane 3: flow-through; lane 4: wash with purification buffer; lane 5: wash with purification buffer containing 10 mM imidazole; lane 6: wash with purification buffer containing 30 mM imidazole; lane 7: elution with purification buffer containing 250 mM imidazole; lane 8: elution with purification buffer containing 500 mM imidazole; lane 9: elution with purification buffer containing 1 M imidazole.

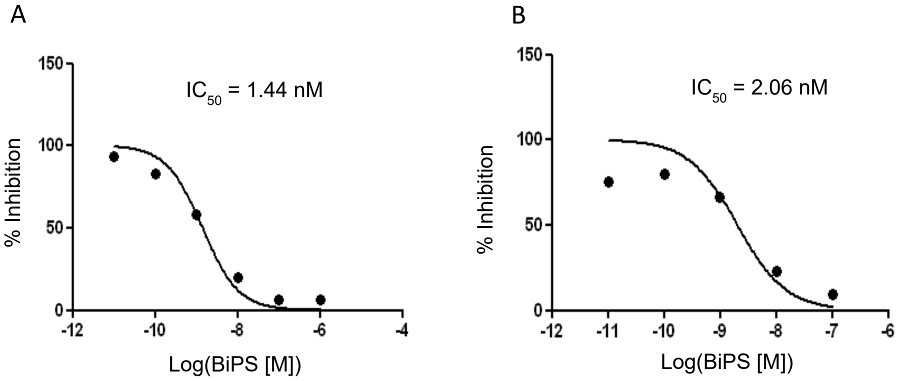

Figure S7. Dose response curve used to determine IC_{50} of BiPS for A) recombinantly expressed catalytic domain of MMP-13, and B) commercially available MMP-13 active protein.

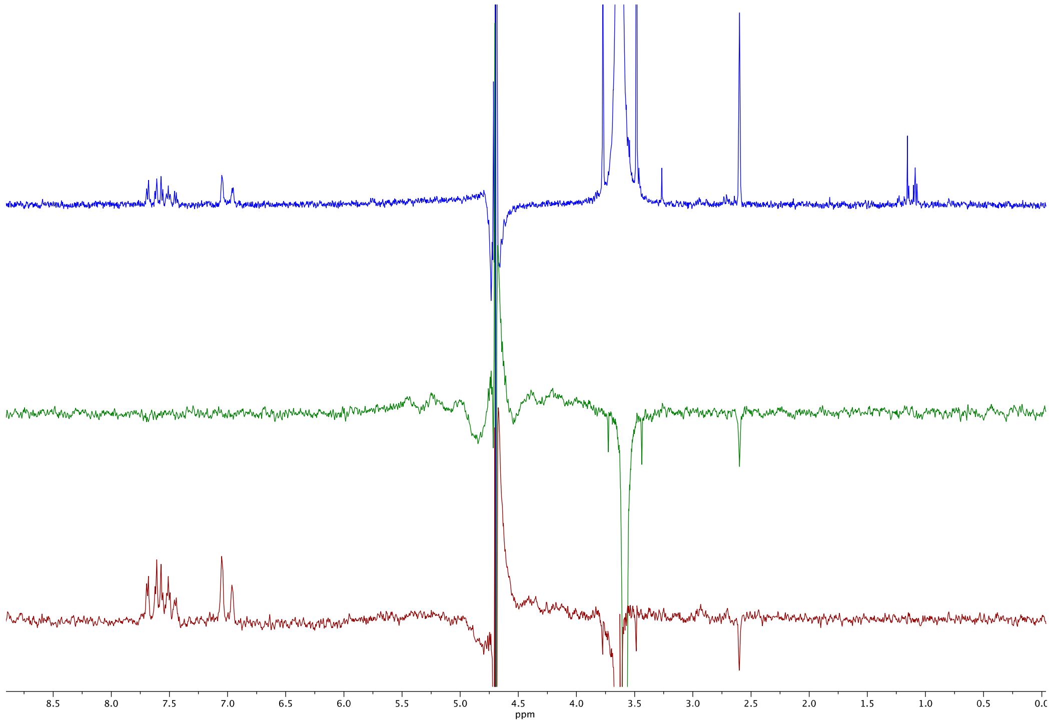

Figure S8. NMR studies for the interaction between the MMP-13 catalytic domain and BiPS. Blue: 1D ^{1}H spectrum of BiPS and the MMP-13 catalytic domain; green: waterLOGSY experiment of BiPS; red: waterLOGSY experiment of BiPS in the presence of the MMP-13 catalytic domain.

PART E. IC_{50} calculations

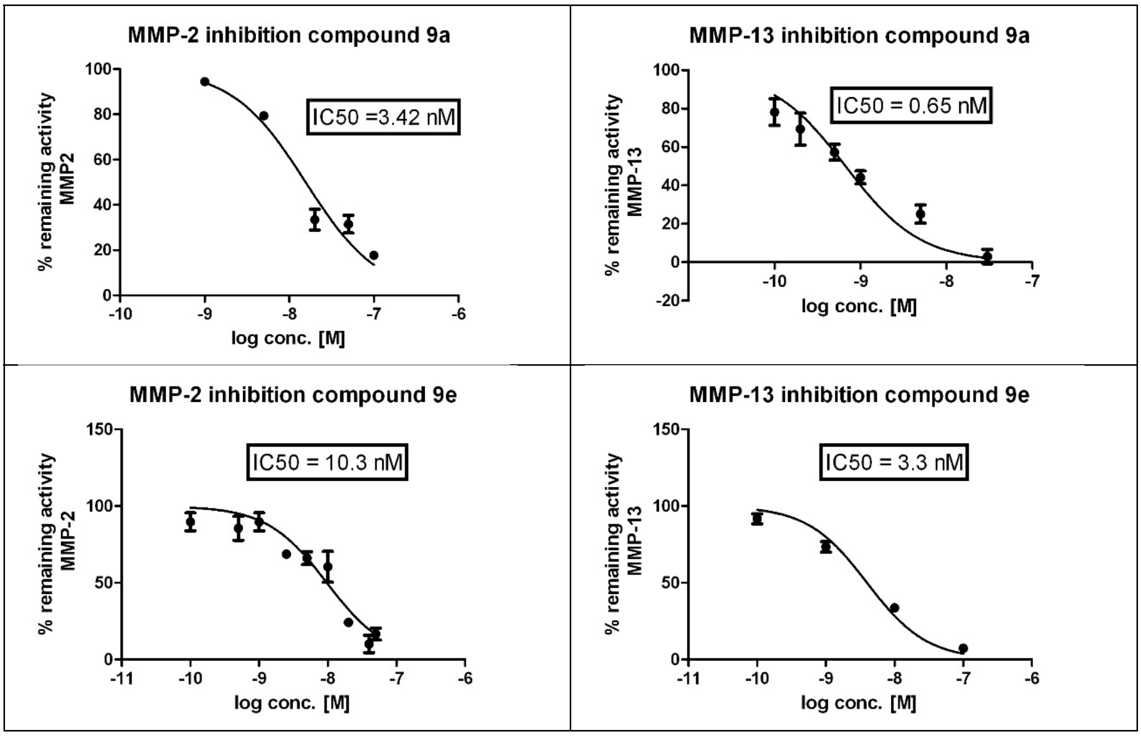

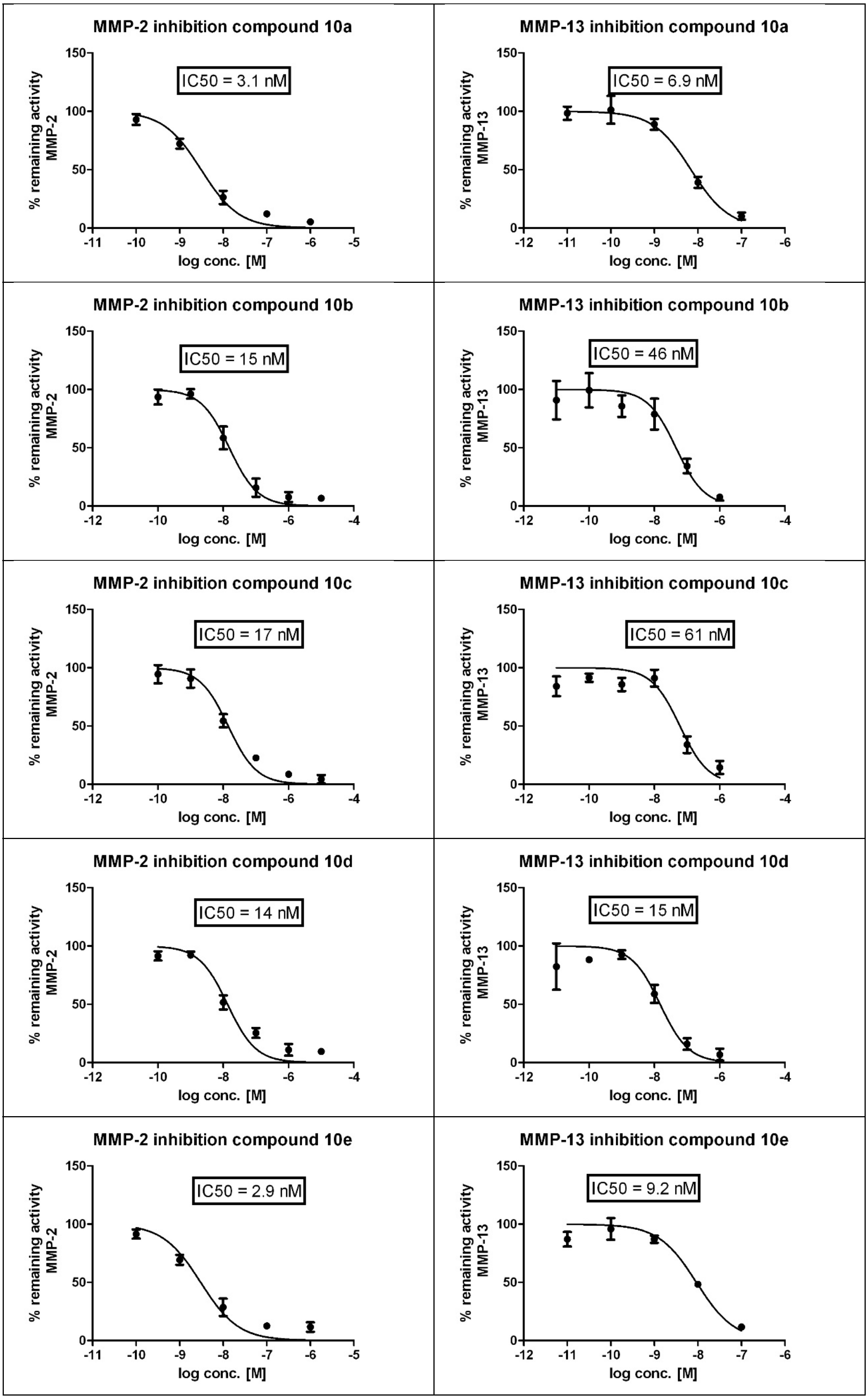

Figure S9. IC_{50} calculations in MMP-13 and MMP-2 for active compounds.

PART F. Purity by HPLC for the final compounds.

HPLC for Compound 9a

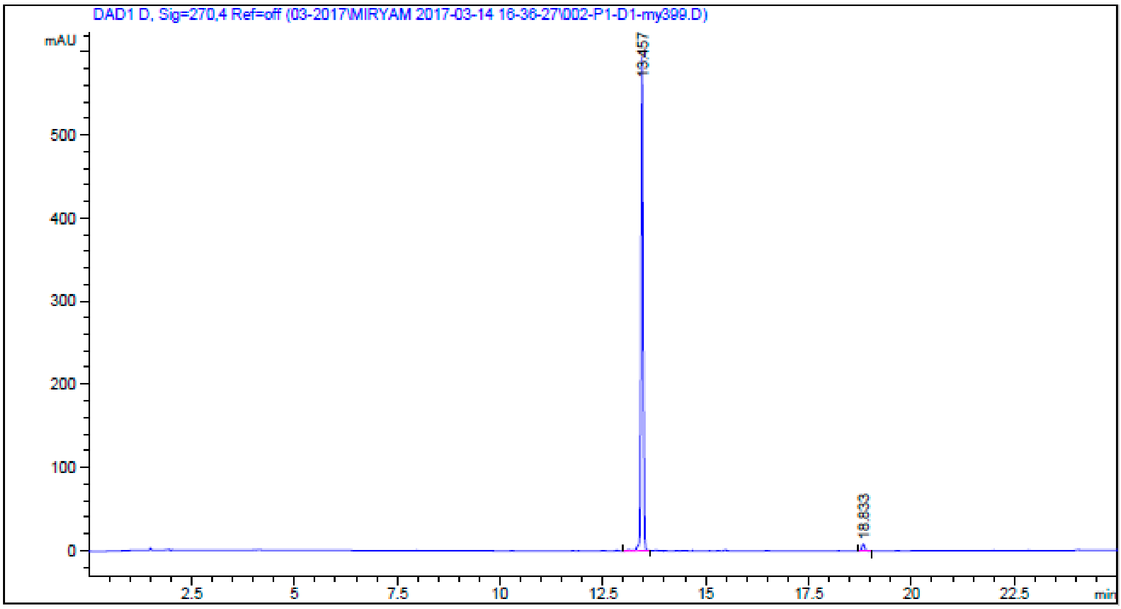

Signal 1: DAD1 D, Sig=270,4 Ref=off

Peak #	RetTime [min]	Type	Width [min]	Area [mAU*s]	Height [mAU]	Area %
1	13.457	VB R	0.0583	2259.47827	595.23047	98.2746
2	18.833	BB	0.0752	39.66923	8.38323	1.7254

Totals : 2299.14750 603.61370

HPLC for Compound 9b

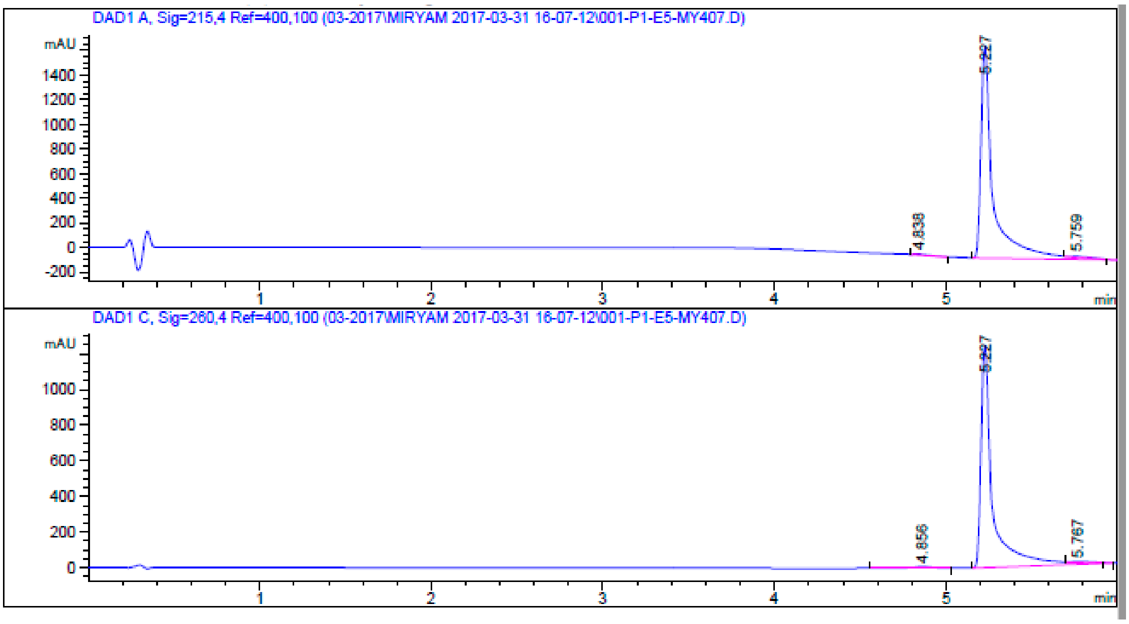

Signal 1: DAD1 A, Sig=215,4 Ref=400,100

Peak #	RetTime [min]	Type	Width [min]	Area [mAU*s]	Height [mAU]	Area %
1	4.838	BB	0.0878	36.47041	5.92225	0.4159
2	5.227	BV R	0.0707	8639.71191	1716.91211	98.5205

Peak #	RetTime [min]	Type	Width [min]	Area [mAU*s]	Height [mAU]	Area %
3	5.759	VBAE	0.1074	93.27305	11.65226	1.0636

Totals : 8769.45538 1734.48662

Signal 2: DAD1 C, Sig=260,4 Ref=400,100

Peak #	RetTime [min]	Type	Width [min]	Area [mAU*s]	Height [mAU]	Area %
1	4.856	BB	0.1171	74.85628	8.46912	1.2333
2	5.227	BV R	0.0675	5943.94629	1249.23083	97.9313
3	5.767	VBAE	0.1091	50.70398	6.35159	0.8354

Totals : 6069.50654 1264.05155

HPLC for Compound 9c

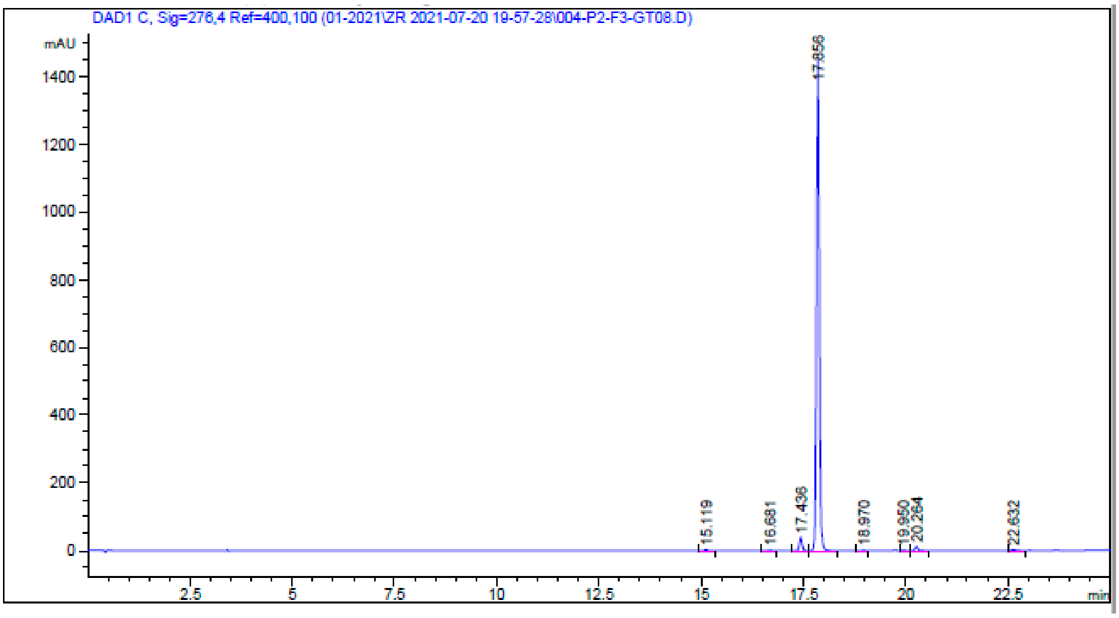

Peak #	RetTime [min]	Type	Width [min]	Area [mAU*s]	Height [mAU]	Area %
1	15.119	BB	0.0775	16.11996	3.16158	0.2147
2	16.681	BB	0.0930	12.50229	1.99875	0.1665
3	17.436	BB	0.0756	196.85049	39.85465	2.6220
4	17.856	BB	0.0757	7194.41211	1454.10107	95.8280
5	18.970	BV	0.0806	6.86097	1.27891	0.0914
6	19.950	BB	0.0798	6.80947	1.32931	0.0907
7	20.264	BV R	0.0800	56.38695	10.60598	0.7511
8	22.632	BB	0.0943	17.68854	2.85532	0.2356
Totals :				7507.63078	1515.18557	

HPLC for Compound 9d

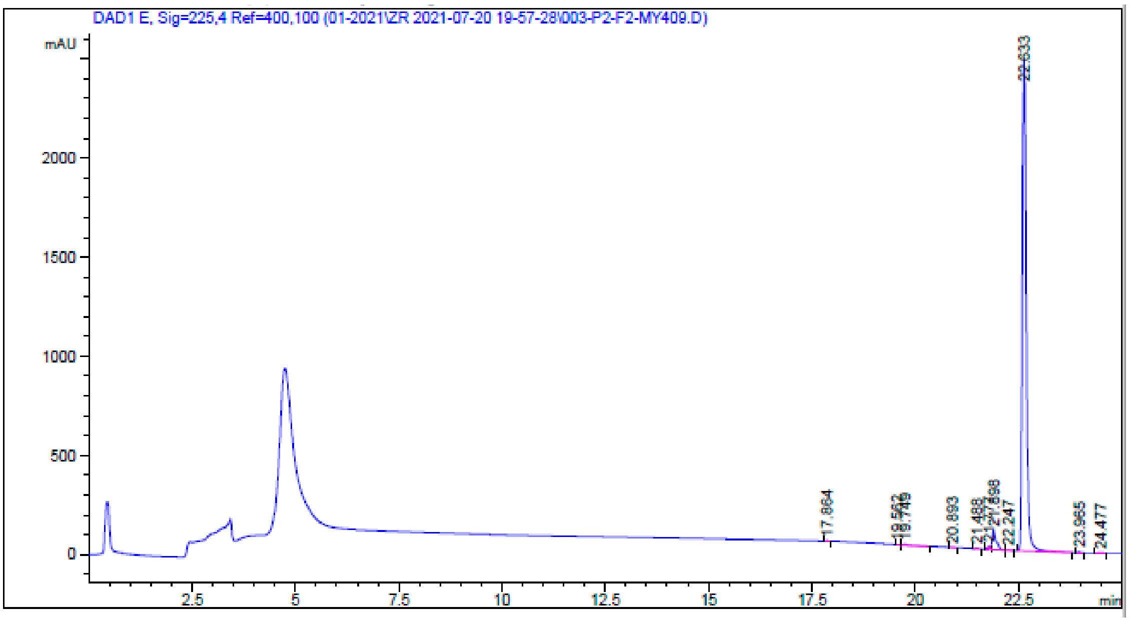

(Blank with DMSO)

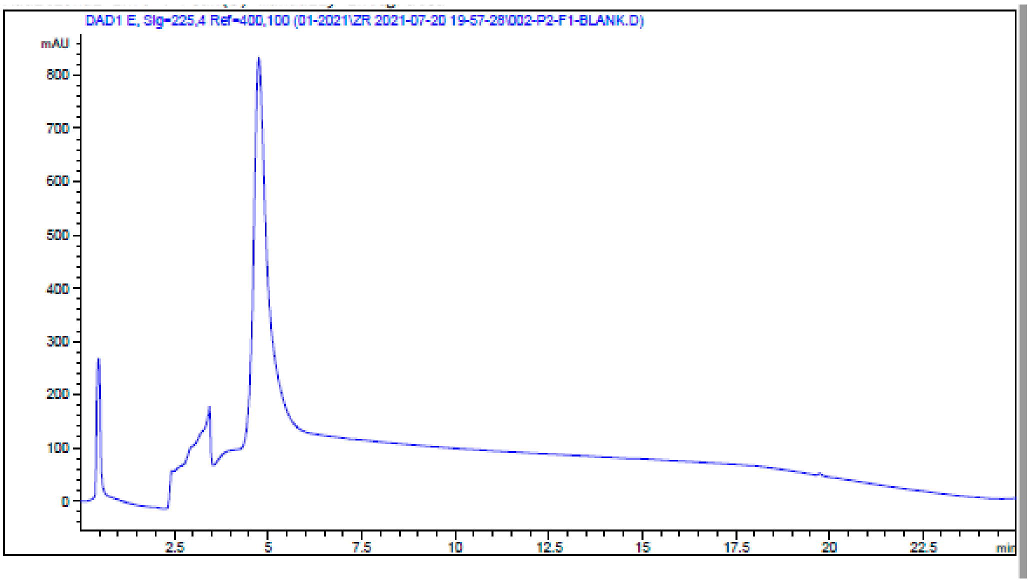

Peak #	RetTime [min]	Type	Width [min]	Area [mAU*s]	Height [mAU]	Area %
1	17.864	BV	0.0895	8.26097	1.34708	0.0477
2	19.562	VV	0.0946	11.94984	1.81893	0.0690
3	19.749	VB	0.1401	47.16131	4.65719	0.2722
4	20.893	BB	0.0779	6.38866	1.28624	0.0369
5	21.488	BB	0.0820	10.55691	1.98744	0.0609
6	21.777	BV E	0.0660	48.15607	11.23072	0.2780
7	21.898	VB R	0.1009	682.95239	96.13583	3.9425
8	22.247	BB	0.0951	7.37572	1.14606	0.0426
9	22.633	BB	0.1033	1.64512e4	2482.61694	94.9675
10	23.965	BB	0.0835	20.65994	3.79639	0.1193
11	24.477	BB	0.1354	28.32399	3.46351	0.1635
Totals :				1.73230e4	2609.48632	

HPLC for Compound 9e

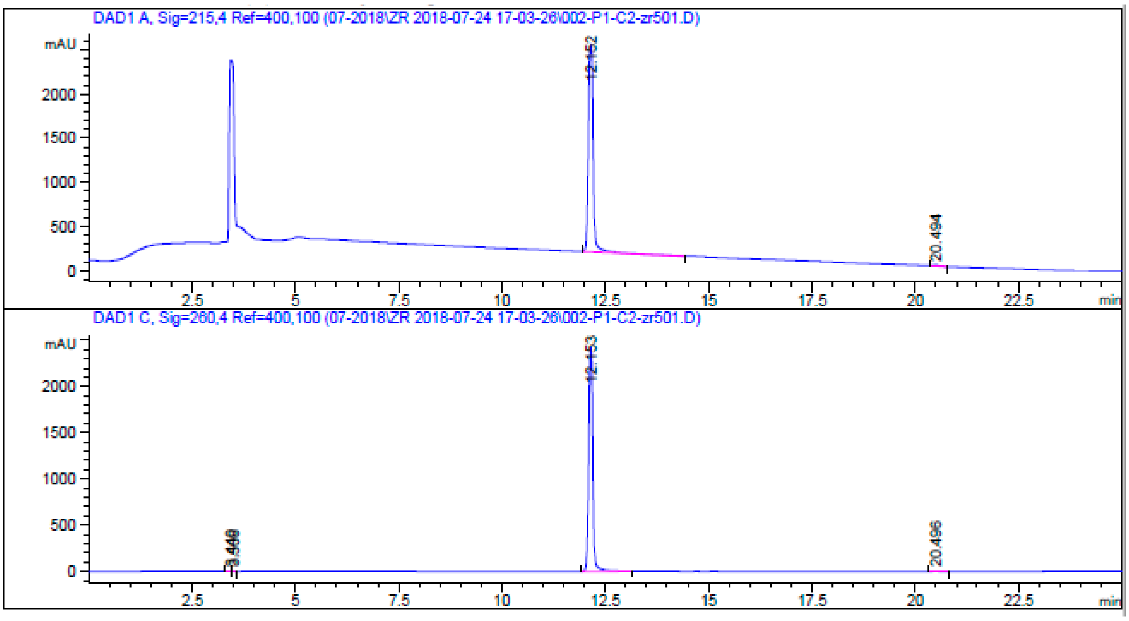

Peak #	RetTime [min]	Type	Width [min]	Area [mAU*s]	Height [mAU]	Area %
1	12.152	BB	0.1277	1.87126e4	2329.23779	99.6838
2	20.494	BB	0.0871	59.35725	10.31442	0.3162

Totals : 1.87719e4 2339.55221

Signal 2: DAD1 C, Sig=260,4 Ref=400,100

Peak #	RetTime [min]	Type	Width [min]	Area [mAU*s]	Height [mAU]	Area %
1	3.440	BV	0.0616	6.71400	1.57931	0.0415
2	3.508	VB	0.0700	7.67466	1.78961	0.0475
3	12.153	BB	0.1034	1.61354e4	2430.89648	99.7939
4	20.496	BB	0.0902	18.93077	3.14770	0.1171

Totals : 1.61687e4 2437.41310

HPLC for Compound 10a

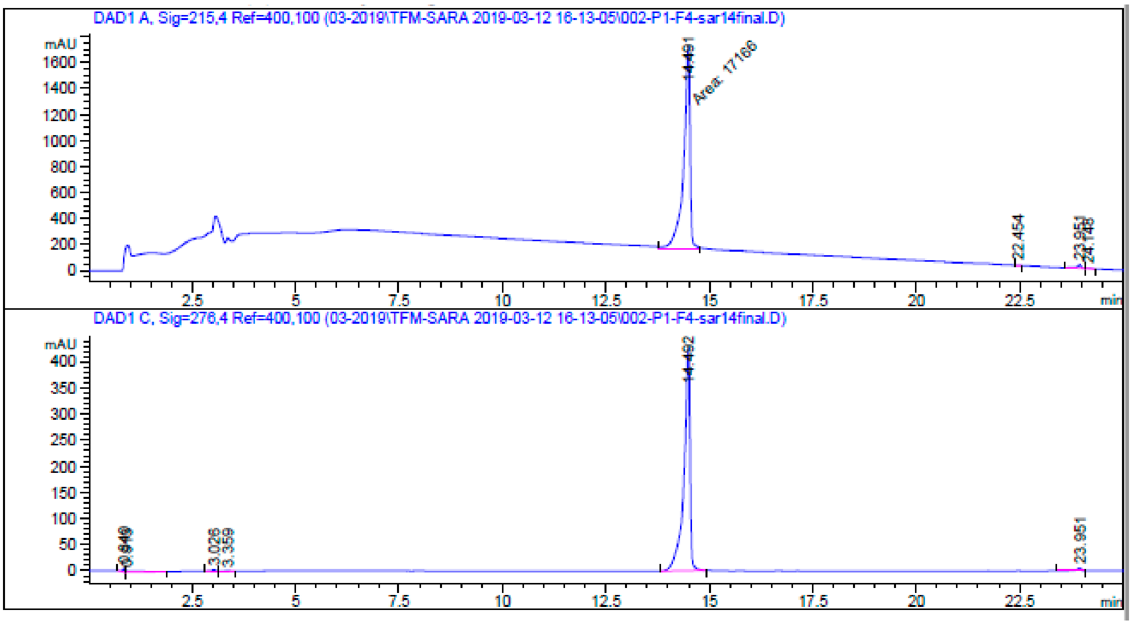

Peak #	RetTime [min]	Type	Width [min]	Area [mAU*s]	Height [mAU]	Area %
1	14.491	MM	0.1828	1.71660e4	1565.27454	98.6214
2	22.454	BB	0.0959	12.01511	2.00604	0.0690
3	23.951	BB	0.1030	195.08801	26.77514	1.1208
4	24.148	BBA	0.1125	32.85143	4.14433	0.1887
Totals :				1.74059e4	1598.20005	

Signal 2: DAD1 C, Sig=276,4 Ref=400,100

Peak #	RetTime [min]	Type	Width [min]	Area [mAU*s]	Height [mAU]	Area %
1	0.840	BB	0.0490	17.75322	5.31471	0.3945
2	0.913	BB	0.2234	44.63164	2.46326	0.9917
3	3.026	BB	0.0876	20.10593	3.18593	0.4467
4	3.359	BB	0.1560	13.92740	1.35393	0.3094
5	14.492	BB	0.1387	4352.80371	427.95197	96.7139
6	23.951	BB	0.1139	51.48116	6.26349	1.1438
Totals :				4500.70307	446.53328	

HPLC for Compound 10b

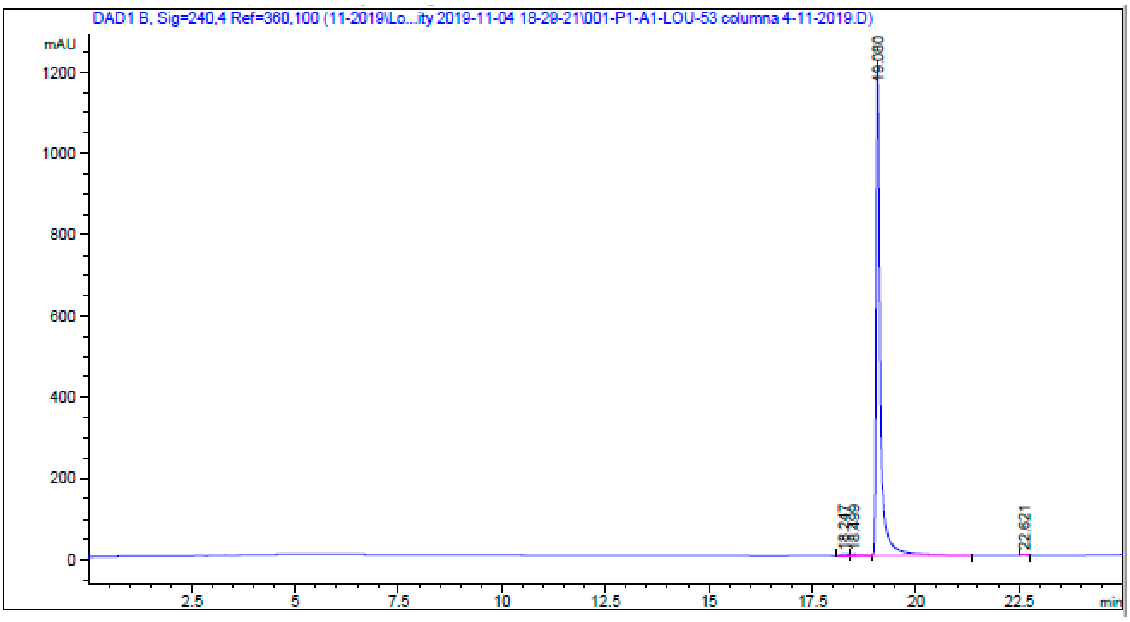

Peak #	RetTime [min]	Type	Width [min]	Area [mAU*s]	Height [mAU]	Area %
1	18.247	BV E	0.2020	41.00013	2.99033	0.4693
2	18.499	VV E	0.2619	51.83469	2.50011	0.5933
3	19.080	VB R	0.1026	8635.04395	1219.69983	98.8448
4	22.621	BB	0.0820	8.08163	1.52106	0.0925
Totals :				8735.96039	1226.71132	

HPLC for Compound 10c

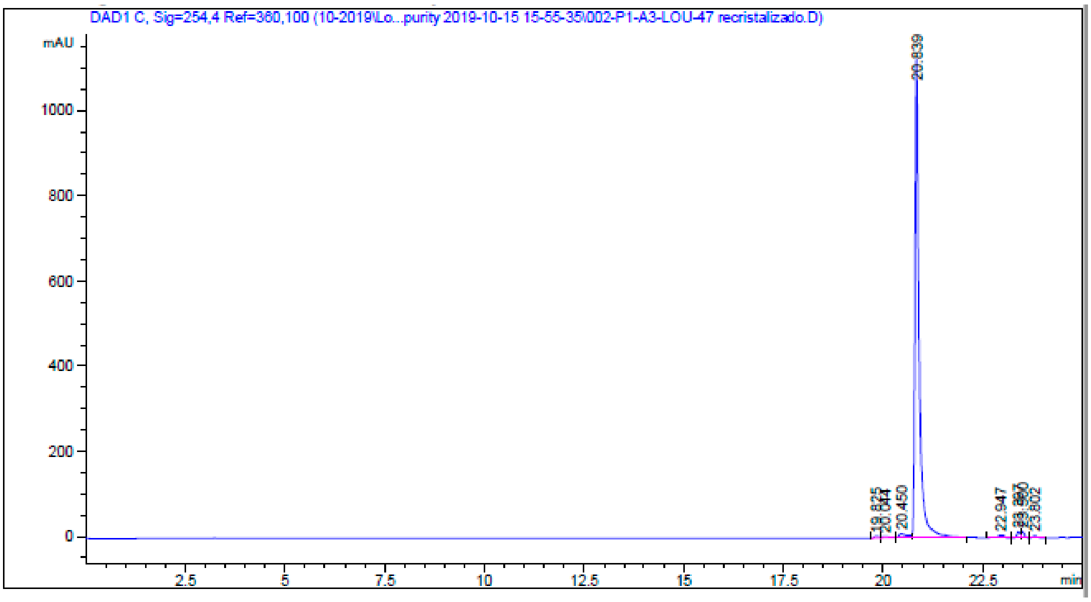

Peak #	RetTime [min]	Type	Width [min]	Area [mAU*s]	Height [mAU]	Area %
1	19.825	BV	0.1249	33.94621	4.00282	0.3982
2	20.044	VB	0.1706	27.32478	2.14544	0.3206

Peak #	RetTime [min]	Type	Width [min]	Area [mAU*s]	Height [mAU]	Area %
3	20.450	BV E	0.2018	118.82546	8.56540	1.3940
4	20.839	VB R	0.1041	8101.08252	1124.42224	95.0401
5	22.947	VB R	0.1428	61.60908	5.67054	0.7228
6	23.397	BV	0.0793	72.70608	13.84328	0.8530
7	23.500	VV	0.0816	79.34451	14.56338	0.9309
8	23.802	VB	0.0961	29.01904	4.56726	0.3404
Totals :				8523.85768	1177.78035	

HPLC for Compound 10d

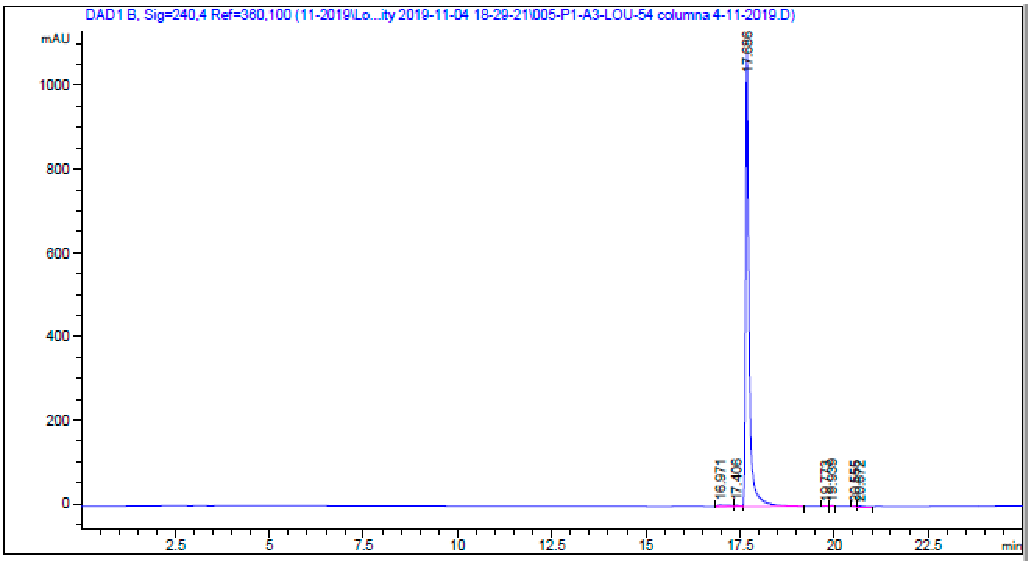

Peak #	RetTime [min]	Type	Width [min]	Area [mAU*s]	Height [mAU]	Area %
1	16.971	BV E	0.2757	92.51758	4.42853	1.2917
2	17.406	VV E	0.1366	31.11412	3.11244	0.4344
3	17.686	VB R	0.0934	7000.71289	1083.49072	97.7423

Peak #	RetTime [min]	Type	Width [min]	Area [mAU*s]	Height [mAU]	Area %
4	19.773	BV	0.0801	5.61738	1.08983	0.0784
5	19.939	VB	0.0852	12.26579	2.26520	0.1713
6	20.555	BV	0.0863	9.44953	1.66328	0.1319
7	20.672	VB	0.0956	10.74185	1.61454	0.1500

HPLC for Compound 10e

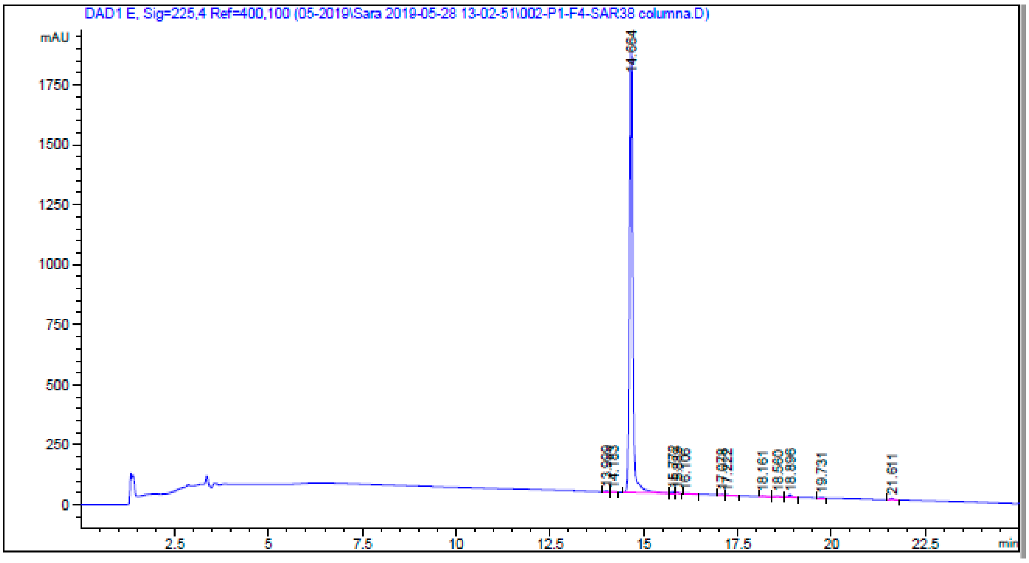

Peak #	RetTime [min]	Type	Width [min]	Area [mAU*s]	Height [mAU]	Area %
1	13.999	BV	0.0754	17.90581	3.64128	0.1674
2	14.183	VB	0.0914	6.80771	1.14525	0.0636

Peak #	RetTime [min]	Type	Width [min]	Area [mAU*s]	Height [mAU]	Area %
3	14.664	BV R	0.0864	1.04494e4	1836.38232	97.6760
4	15.772	BV	0.0795	32.74617	6.21522	0.3061
5	15.889	VB	0.0746	24.74622	4.92883	0.2313
6	16.106	BB	0.1176	12.60213	1.47627	0.1178
7	17.078	BV	0.0856	11.97303	2.12864	0.1119
8	17.222	VB	0.1257	10.50355	1.15931	0.0982
9	18.161	BB	0.0884	9.88424	1.63735	0.0924
10	18.560	BV	0.0952	20.95824	3.34188	0.1959
11	18.896	VB	0.0857	51.57423	8.88674	0.4821
12	19.731	BB	0.0817	14.04190	2.65661	0.1313
13	21.611	BB	0.0853	34.88150	6.23371	0.3261
Totals :				1.06980e4	1879.83341	

Figure S10. HPLC results for final compounds.
